# Mitochondrial protein import stress augments α-synuclein aggregation and neural damage

**DOI:** 10.26508/lsa.202603770

**Published:** 2026-11-10

**Authors:** Liam P Coyne, Arnav Rana, Xiaowen Wang, Joseph Mauro, Sanaea Bhagwagar, Yumiko Umino, Eduardo C Solessio, Frank Middleton, Xin Jie Chen

**Affiliations:** 1 Department of Biochemistry and Molecular Biology, State University of New York Upstate Medical University , Syracuse, NY, USA; 2 Center for Vision Research, Department of Ophthalmology, State University of New York Upstate Medical University , Syracuse, NY, USA; 3 Department of Neuroscience and Physiology, State University of New York Upstate Medical University , Syracuse, NY, USA

## Abstract

Genetic and environmental factors are known to converge on mitochondria to cause Parkinson’s disease. The underlying mechanism is incompletely understood. In addition to bioenergetic defects, nonbioenergetic pathways have been largely unexplored. Validating the nonbioenergetic mitochondrial stressors as independent pathogenic factors is challenging because most mouse models of mitochondrial stress are confounded by bioenergetic defects. Here, we show that expression of Ant1^P.A114P,A123D^, previously shown to cause mitochondrial protein import clogging in cultured human cells, does not affect the overall bioenergetic capacity, yet it induces proteostatic stress responses in the cytosol that suggests accumulation of unimported precursor proteins. In a Parkinson’s disease model expressing α-synuclein (A53T), Ant1^P.A114P,A123D^ increased the size of α-synuclein aggregates, increased aggregation of mitochondrial preproteins, heightened neuroinflammation, and worsened motor coordination defects. Importantly, we found no evidence of bioenergetic defects in either α-synuclein (A53T) or α-synuclein (A53T) + Ant1^P.A114P,A123D^ mice. These data suggest that Ant1^P.A114P,A123D^ likely induces mitochondrial protein import stress, and contributes to neural damage through cytosolic proteostatic stress and altered aggregation dynamics of neuropathogenic proteins independent of bioenergetics.

## Abstract (precis)

Mitochondrial protein import stress induces proteostatic stress in the cytosol, which alters α-synuclein aggregation dynamics and augments motor dysfunction independent of bioenergetic defects.

## Introduction

Mitochondrial defects and cytosolic protein aggregation in neurons are comanifested across neurodegenerative diseases such as Parkinson’s disease (PD), Lewy body dementia, Alzheimer’s disease, amyotrophic lateral sclerosis (ALS), and Huntington’s disease (Gispert et al, 2009; Schon & Przedborski, 2011; Swerdlow, 2011; Pickrell et al, 2015; Spano et al, 2015). These two hallmarks are virtually ubiquitous despite significant variations in genetic and phenotypic makeup among diseases. Although both hallmarks can individually drive neurodegeneration, whether and how they interact during initiation and/or progression of these complex diseases is poorly understood. The effects of pathogenic cytosolic protein species on mitochondrial function have been extensively investigated (Kawamata & Manfredi, 2017). By comparison, whether and how mitochondrial damage affects cytosolic proteostasis and pathological protein aggregation is vastly understudied.

Recent studies have shown that a wide range of mitochondrial stressors can affect cell fitness and viability by reducing the import of mitochondrial preproteins, causing their toxic accumulation and aggregation in the cytosol (Wang & Chen, 2015; Wrobel et al, 2015; Liu et al, 2019; Nowicka et al, 2021; Schlagowski et al, 2021; Groh et al, 2023). This cell stress mechanism was termed mitochondrial precursor overaccumulation stress (mPOS) (Wang & Chen, 2015; Coyne & Chen, 2026). In the context of neurodegeneration, this raises the intriguing possibility that mitochondrial dysfunction could contribute to cytosolic protein aggregation, either by directly causing proteotoxicity or by amplifying preexisting proteostatic stress in the cytosol driven by misfolded pathogenic proteins (e.g., α-synuclein [α-syn] in PD) (Coyne & Chen, 2018). The critical barrier to testing this idea in vivo has been the lack of an adequate animal model of nonlethal mitochondrial protein import stress, especially one that isolates the effect of protein import defects in the absence of confounding bioenergetic defects. Such a model is essential for testing the pathogenicity of protein import stress and mPOS.

Modeling mitochondrial protein import stress in animals is particularly challenging because virtually all mitochondrial functions, with some being essential for cell survival, depend on efficient import through a limited number of protein translocase channels. All but 13 of the 1,000–1,500 mitochondrial proteins are encoded in the nucleus, synthesized by cytosolic ribosomes, and then imported into the organelle. The import machinery is intricate, relying on numerous chaperones both inside and outside mitochondria, as well as multiple pore-forming protein complexes in the inner and outer mitochondrial membranes (Neupert & Herrmann, 2007; Endo & Yamano, 2009; Wiedemann & Pfanner, 2017). The sole entry point for >90% of mitochondrial proteins is the translocase of the outer membrane (TOM) complex. We previously engineered clinically relevant mutant variants of the yeast mitochondrial ADP/ATP carrier protein Aac2 and its human homolog ANT1 (or SLC25A4). We found that the mutant proteins can partially clog the mitochondrial protein import pathway and obstruct general protein import (Coyne et al, 2023). The best-characterized clogger mutant protein was the yeast Aac2p^A128P,A137D^ that has been shown to cause a severe cell growth defect by preferentially clogging the TOM complex. Specifically, Aac2p^A128P,A137D^ accumulated at the TOM complex in cells and was unable to efficiently traverse the outer membrane in the *in organello* protein import assay. In human cells, ANT1^A114P,A123D^, the equivalent of the yeast Aac2p^A128P,A137D^, also accumulated at the TOM complex and caused global retention of mitochondrial proteins in the cytosol. In a mouse model expressing *Slc25a4*^*P.A114P,A123D*^ (“clogger” mice), we similarly found that unimported mitochondrial proteins accumulate in the cytosol of skeletal muscle. We found that the clogger protein is highly unstable in vivo, and is present at ∼0.1% of the WT level. As such, mitochondrial respiration is only mildly affected in the skeletal muscle of these mice. This correlates with a mild mitochondrial myopathy in aged animals. Interestingly, ∼4% of the clogger mice become paralyzed around 12 mo of age, suggesting a direct role of protein import clogging in neurodegeneration. However, the vast majority (∼96%) of clogger mice have a normal lifespan and do not develop overt neurological symptoms. Overall, our prior observations suggest that mitochondrial protein import is only mildly clogged in this model, presenting an opportunity to study the (patho)physiological effects of a mild reduction in mitochondrial protein import efficiency.

In the present study, we used the clogger mice to study the specific effects of Ant1^P.A114P,A123D^ expression on stress response and neurological function. Most importantly, we then used mouse genetics to test whether and how protein import stress might enhance neural damage in a mouse model of PD that involves the cytosolic misfolding and aggregation of a mutant form of α-syn (A53T) that is a dominant cause of familial PD.

## Results

### Ant1^P.A114P,A123D^ does not affect mitochondrial bioenergetics, motor coordination, or cognitive function

**Figure 1.**
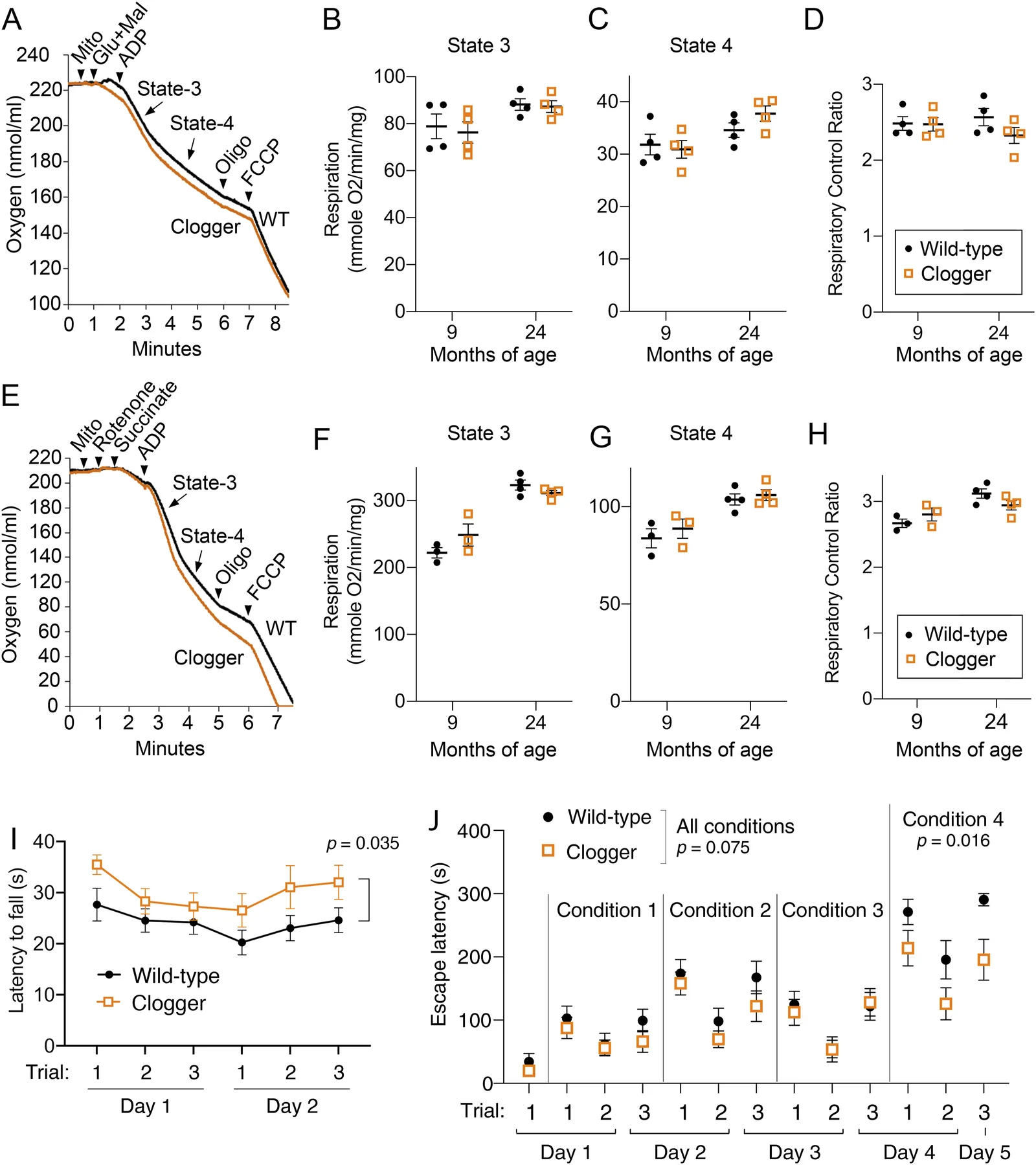
Mild mitochondrial protein import clogging with Ant1^P.A114P,A123D^ does not affect mitochondrial bioenergetics but moderately improves motor coordination. **(A, B, C, D)** Respirometry of purified brain mitochondria with complex I stimulated by glutamate (glu) and malate (mal). N = 4 mice/genotype/age group. Two measurements were taken per mouse, the average of which is plotted in (B, C, D). Data from different ages were analyzed separately because of a suspected batch effect. Independent repeated-measures ANOVA within each age group (with measurement order as the within-subjects variable and genotype as the between-subjects variable) confirmed no effect of measurement order or genotype for any measure plotted in (B, C, D) or (F, G, H). FCCP, trifluoromethoxy carbonylcyanide phenylhydrazone; Oligo, oligomycin. State 3 respiration is the maximal respiratory rate after addition of ADP. State 4 respiration is the respiratory rate after depletion of ADP. The respiratory control ratio is State 3 divided by State 4 respiratory rates. Decreased respiratory control ratio would be interpreted as increased mitochondrial damage. Horizontal bars indicate average values ± SEM. **(E, F, G, H)** Respirometry of purified brain mitochondria with complex II stimulated by succinate and complex I inhibited by rotenone. N = 3 mice/genotype at 9 mo of age and 4 mice/genotype at 24 mo of age. Data were collected and analyzed as in (A, B, C, D). **(I)** Ant1^P.A114P,A123D^ (clogger) mice perform better than WT on the accelerating rotarod at 19–22 mo of age (n > 16 mice per genotype; n > 5 per sex per genotype). Average values are plotted ± SEM. Data were analyzed with a two-way repeated-measures ANOVA probing for an effect of sex and genotype, with Geisser–Greenhouse correction. Sexes were pooled for data presentation as there was no detectable main effect on sex (*P* = 0.22) or sex interaction effects. Depicted is the main effect of genotype. **(J)** Ant1^P.A114P,A123D^ (clogger) mice have moderately enhanced executive function, as judged by speed in removing obstacles from a 5 × 5 cm doorway to escape from a stressful environment. Mice were 20–23.5 mo of age; n = 13 WT (5 females, 8 males), 15 cloggers (9 females, 6 males). Different “Conditions” are different obstacles. Average values are plotted ± SEM. Data were analyzed by repeated-measures ANOVA with Geisser–Greenhouse correction.

**Figure S1.**
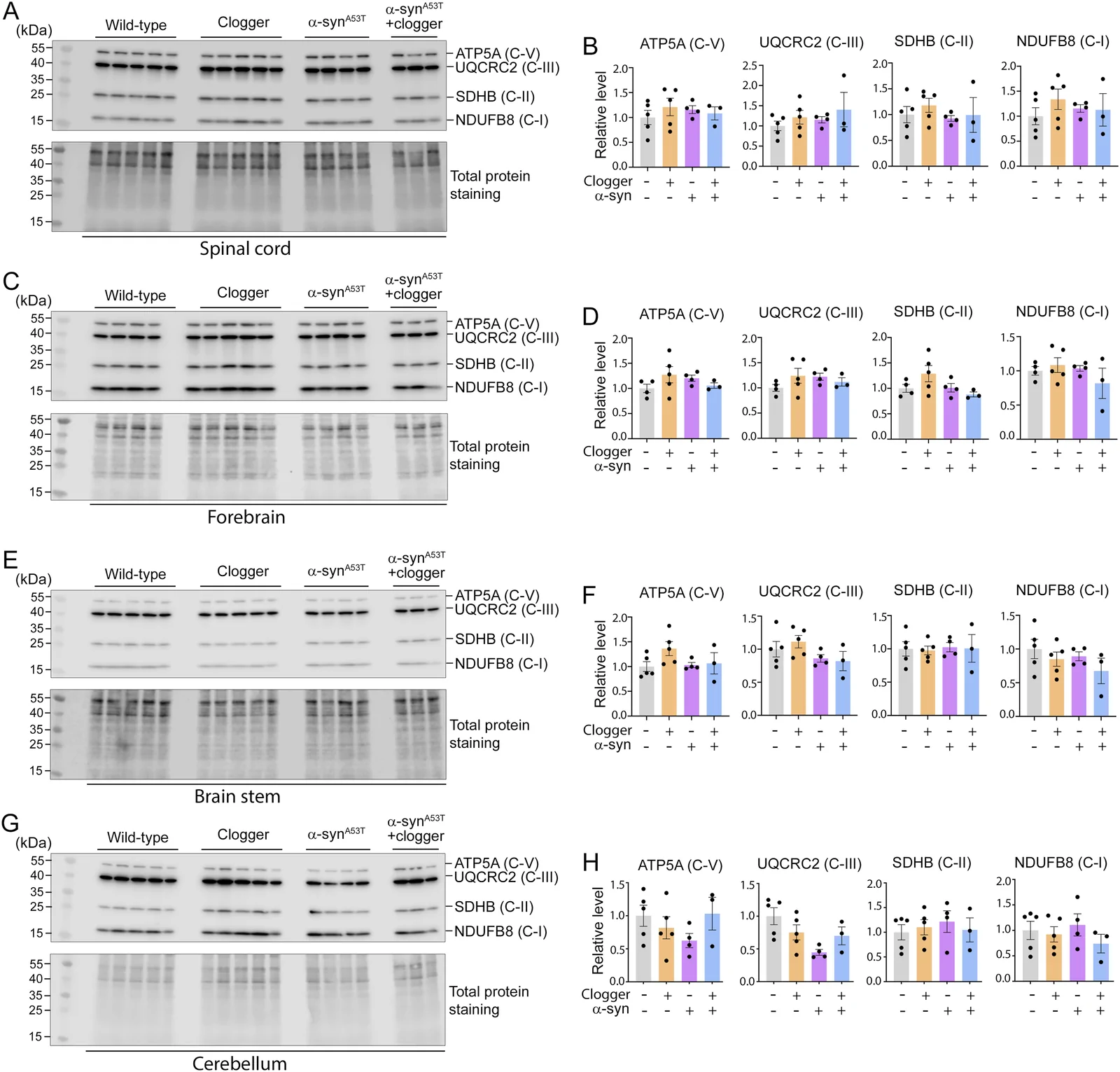
Western blot analysis showing the integrity of the OXPHOS pathway, using a cocktail of antibodies against the indicated mitochondrial proteins. Related to Fig 1. C-I, C-II, C-III, and C-V, respiratory complexes I, II, III, and V, respectively. **(A, C, E, G)** Crude mitochondria isolated spinal cord, forebrain, brainstem, and cerebellum of 9-mo-old mice, respectively. **(B, D, F, H)** Quantification of (A), (C), (E), and (G), respectively. The *P*-values are >0.05 between the four genotypes in all the three regions of CNS, as determined by a *t* test. Each lane/data point represents an individual mouse.

The human ANT1^P.A114P^ protein is pathogenic, causing autosomal dominant progressive external ophthalmoplegia (Kaukonen et al, 2000). In addition to progressive external ophthalmoplegia and skeletal muscle phenotypes, ANT1-induced autosomal dominant progressive external ophthalmoplegia also associates with neurological and psychiatric phenotypes such as sensorineural hearing loss, cognitive impairment, dementia, and bipolar and schizoaffective disorders (Kaukonen et al, 1999; Napoli et al, 2001; Simoncini et al, 2017). ANT1^P.A123D^ is associated with myopathy and cardiomyopathy (Palmieri et al, 2005). The Ant1^P.A114P,A123D^/+ “clogger” mice were generated to facilitate phenotypic scoring and evaluate the in vivo effects of partial protein import clogging. The Ant1^P.A114P,A123D^ clogger is highly unstable and is subject to proteolysis from the IMS side. Although Ant1^P.A114P,A123D^ accumulates to just 0.1% of the WT level, two mutations together resulted in the partial clogging of the TOM complex that can directly be scored by biochemical assays in the skeletal muscle (Coyne et al, 2023). To evaluate mitochondrial bioenergetics in the central nervous system (CNS) of nonparalytic clogger mice, we measured oxygen consumption from purified brain mitochondria. We found that brain mitochondria from clogger mice at the age of 9 and 24 mo do not have reduced maximal respiratory rate (state 3) when stimulating complex I (Fig 1A and B). ADP-depleted respiration (state 4) and the respiratory control ratio (state 3/state 4) were also unchanged (Fig 1C and D). These observations suggest that mitochondrial oxygen consumption is well coupled with ATP synthesis and the maximal complex I–based respiratory capacity is not reduced in clogger brain mitochondria. Mitochondrial respiration was also unaffected when complex I is inhibited with rotenone and complex II–based respiration is stimulated with succinate (Fig 1E–H). Thus, mitochondrial respiration is unaffected or minimally affected in the brain of clogger mice. Consistent with this, we assessed the steady-state levels of respiratory chain proteins by immunoblot and found that representative subunits of complexes I, II, III, and V were unaffected in different CNS tissues from the clogger mice when compared to the WT (Fig S1A–H). Taken together, these data suggest that the bioenergetic function of mitochondria is generally preserved in the CNS of clogger mice.

**Figure S2.**
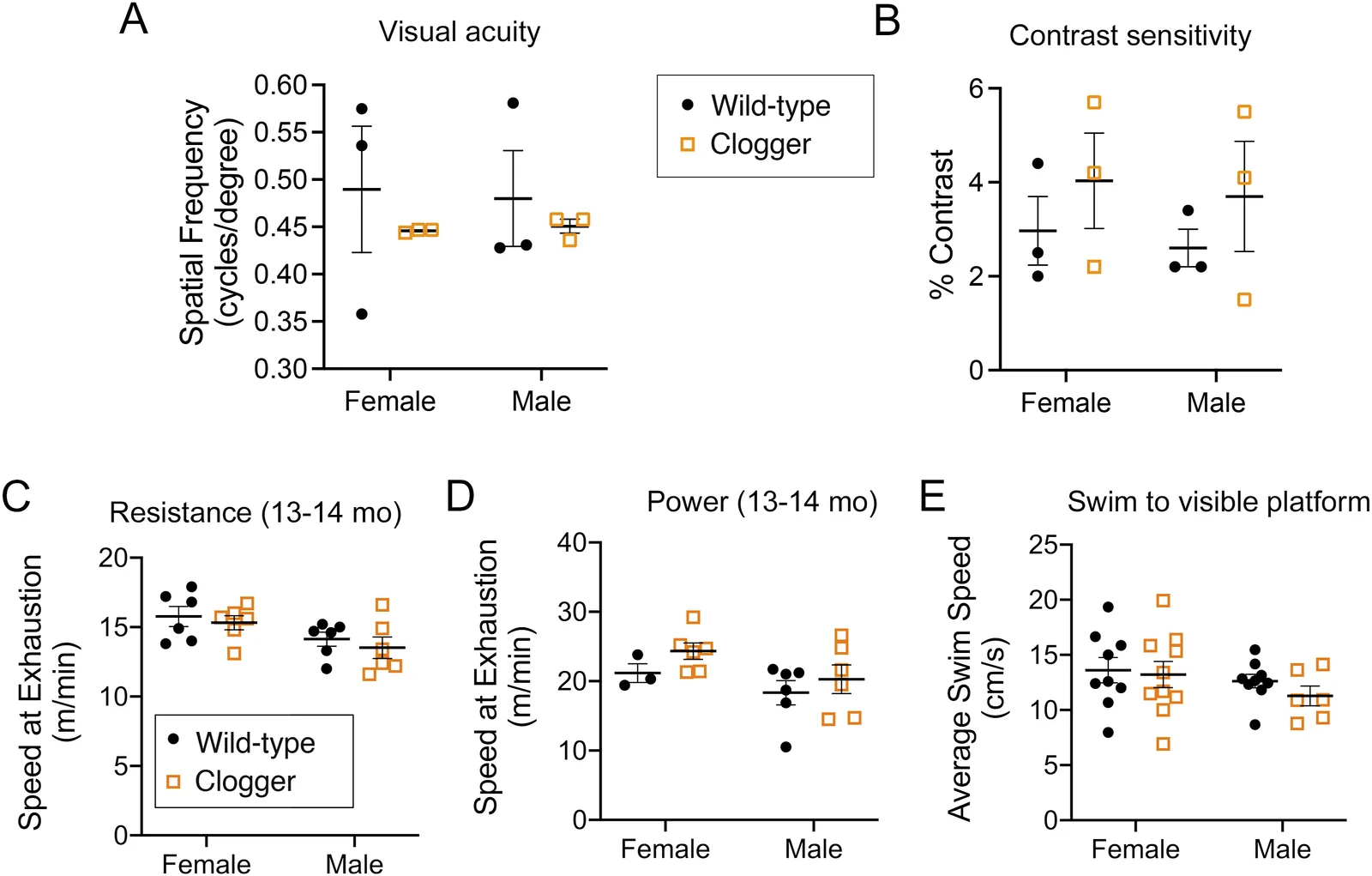
Visual and skeletal muscle functions are not impaired in middle-aged clogger mice. Related to Fig 1. **(A)** Visual acuity of clogger mice is intact at 17–18 mo of age, as suggested by optomotor response testing (n = 3/sex/genotype). **(B)** Visual contrast sensitivity of clogger mice is intact at 17–18 mo of age, as suggested by optomotor response testing (n = 3/sex/genotype). **(C)** Clogger mice do not show exercise intolerance at 13–14 mo of age, as suggested by similar time to exhaustion when forced to run on a slowly accelerating treadmill (“resistance test”) (n = 6/sex/genotype). **(D)** Clogger mice do not show exercise intolerance at 13–14 mo of age, as suggested by similar time to exhaustion when forced to run on a quickly accelerating treadmill (“power test”) (n = 3–6/sex/genotype). **(E)** Clogger mice swim to a visible escape platform at similar average speeds as WT mice, suggesting preserved muscle function. These mice were 19–22 mo of age (n = 6–10/sex/genotype). Horizontal bars indicate average values ± SEM.

To explore the effects of Ant1^P.A114P,A123D^ expression on neurological function, we performed an array of behavioral assays on nonparalytic clogger mice. To ensure validity of the assays given the effects of human ANT1 mutations on oculomotor control and skeletal muscle, we first assessed basic vision and motor function. Using optomotor response testing, we confirmed that visual acuity and contrast sensitivity are preserved in clogger mice at 17–18 mo of age (Fig S2A and B, n = 3/sex/genotype). Although 30-mo-old clogger mice clearly exhibit skeletal muscle atrophy and weakness (Coyne et al, 2023), this effect appears to be age-dependent, as mice at the age of ∼13 mo have preserved muscle function, as suggested by treadmill exhaustion testing (Fig S2C and D, n = 3–6/sex/genotype) and swim speed (Fig S2E, n = 6–10/sex/genotype). We also tested ∼20-mo-old mice on an accelerating rotarod to measure motor coordination and balance. Surprisingly, we found that the clogger mice performed slightly better than WT mice (Fig 1I, n> 5/sex/genotype). Male and female sexes were pooled for analysis as no significant sex effect was found on two-way repeated-measures ANOVA testing (*P* = 0.22). Improved rotarod testing has previously been observed in another mild mouse model of mitochondrial disease (Emmerzaal et al, 2020).

**Figure S3.**
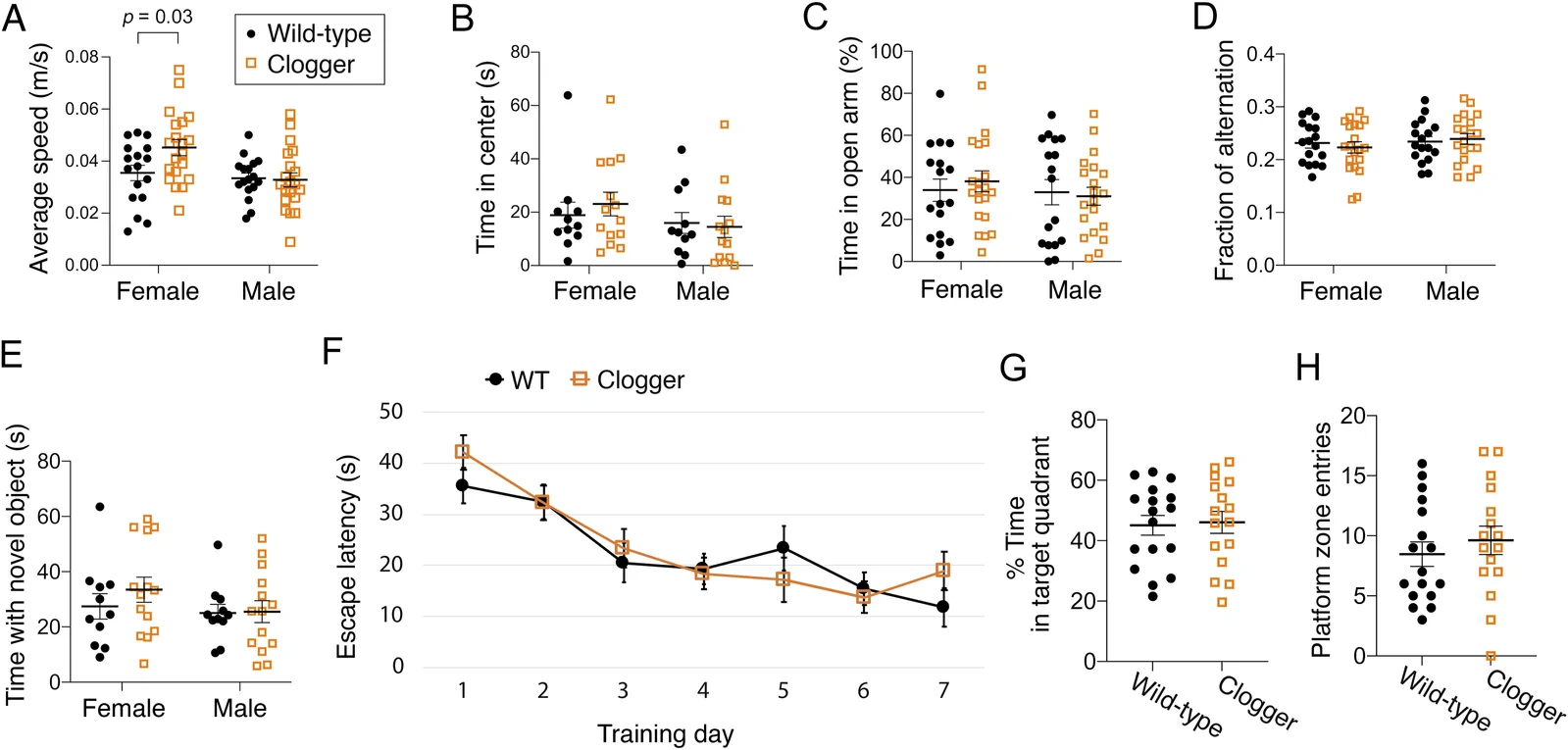
Clogger mice demonstrate sex-dependent increases in locomotor activity, but no changes in anxiety-like behavior or memory. Related to Fig 1. **(A)** Female clogger mice have increased spontaneous locomotor activity. Two independent aged mouse cohorts (14–17 mo of age) were monitored in the open field apparatus, and average speed throughout the 10 min was plotted. Data were analyzed with a two-way ANOVA with Sidak’s multiple comparisons test. Each data point is a biological replicate (n = 17–20/sex/genotype). **(B)** Clogger mice show unchanged levels of anxiety-like behavior, as indicated by the amount of time mice spent in the center versus the edges of the open field apparatus. Each data point is a biological replicate (n = 11–14/sex/genotype). **(C)** Clogger mice show unchanged levels of anxiety-like behavior, as indicated by the amount of time mice spent in the open versus closed arms in the EPM. Each data point is a biological replicate (n = 17–21/sex/genotype). **(D)** Clogger mice do not have impaired spatial working memory, as indicated by Y-maze testing. Each data point is a biological replicate (n = 17–20/sex/genotype). Fraction of Alternation = (# of complete triads)/(# arm entries – 2). A triad is when a mouse enters all three arms consecutively without reentering a previously visited arm in between. **(E)** Clogger mice do not have impaired long-term object memory, as indicated by the amount of time mice spent interacting with a novel object. Each data point is a biological replicate (n = 11–14/sex/genotype). **(F)** Clogger mice do not have spatial learning defects, as indicated by Morris water maze testing. Plotted is the average latency to escape from water to a hidden platform during seven training days. Each data point is a biological replicate (4 trials/day, n = 6–10/sex/genotype). **(G)** Clogger mice do not have impaired long-term memory, as indicated by the probe trial after 7 d of training in the Morris water maze. The target quadrant of the swimming pool contained the hidden platform during training but was removed for the probe trial. Each data point is a biological replicate (n = 6–10/sex/genotype). **(H)** Clogger mice do not have impaired long-term memory, as indicated by the probe trial after 7 d of training in the Morris water maze. Plotted is the number of times the mice swam into the “platform zone,” that is, the area where the hidden platform was during training days. Horizontal bars indicate average values ± SEM. Each data point is a biological replicate. Mice in this figure were 14–17 mo of age.

To assess general locomotor activity, we performed an open field test. When left undisturbed in a brightly lit open field for 10 min, female clogger mice move at a faster average speed compared with WT at 14–17 mo of age (Fig S3A), reminiscent of another mouse model of mitochondria-induced progressive external ophthalmoplegia (Tyynismaa et al, 2005). However, increased locomotor activity could not be attributed to increased anxiety-like behavior (Fig S3B and C). To assess cognitive function, we monitored animal behavior in several assays sensitive to short-term and long-term memory in this aged cohort (14–17 mo of age). The Y-maze spontaneous alternation test relies on rodents’ natural tendency to explore new areas. When they repeatedly return to arms of the Y-maze that they most recently visited, this is interpreted as a defect in working spatial memory. Results from this assay suggested that clogger mice have preserved spatial working memory (Fig S3D). The novel object recognition test relies on rodents’ natural tendency to explore new objects after becoming familiarized with other objects over multiple prior training days. The data from this assay suggested that clogger mice have preserved long-term recognition memory (Fig S3E). The Morris water maze tests rodents’ ability to learn and remember the location of a hidden escape platform that they must swim to, which is unpleasant for rodents. Results from this assay suggested that clogger mice have preserved long-term spatial memory and learning (Fig S3F–H). We note that the water maze data are shown with sexes consolidated, as two-way ANOVA did not show an effect from sex (zone entries, *P* = 0.48; percent time in target quadrant, *P* = 0.57). Collectively, these data suggest that learning and both short-term and long-term memory are intact in clogger mice.

Finally, we tested executive function, which refers to higher level processing used to control and coordinate behavior in response to changing task demands. To test this, we used a “puzzle box” assay in which mice are placed in a stressful environment and the amount of time it takes to escape through an obstructed exit doorway is used as a proxy for executive function (Ben Abdallah et al, 2011; O’Connor et al, 2014). We found that for the most difficult obstruction (“Condition 4”), Ant1^P.A114P,A123D^ mice were significantly better at escaping the stressful environment (Fig 1J). Therefore, clogger mice may have improved executive function in a manner that cannot be attributed to increased anxiety-like behavior. Further experiments with increased animal numbers are needed to clarify whether this is sex-dependent. Overall, these behavioral data are critical for establishing that Ant1^P.A114P,A123D^ alone does not cause neurological dysfunction in nonparalytic mice, although we cannot exclude the possibility that the improved motor coordination and executive function in these mice may have confounded some of the assays for cognitive functions.

### Transcriptional remodeling in the CNS of Ant1^P.A114P,A123D^/+ mice

**Figure 2.**
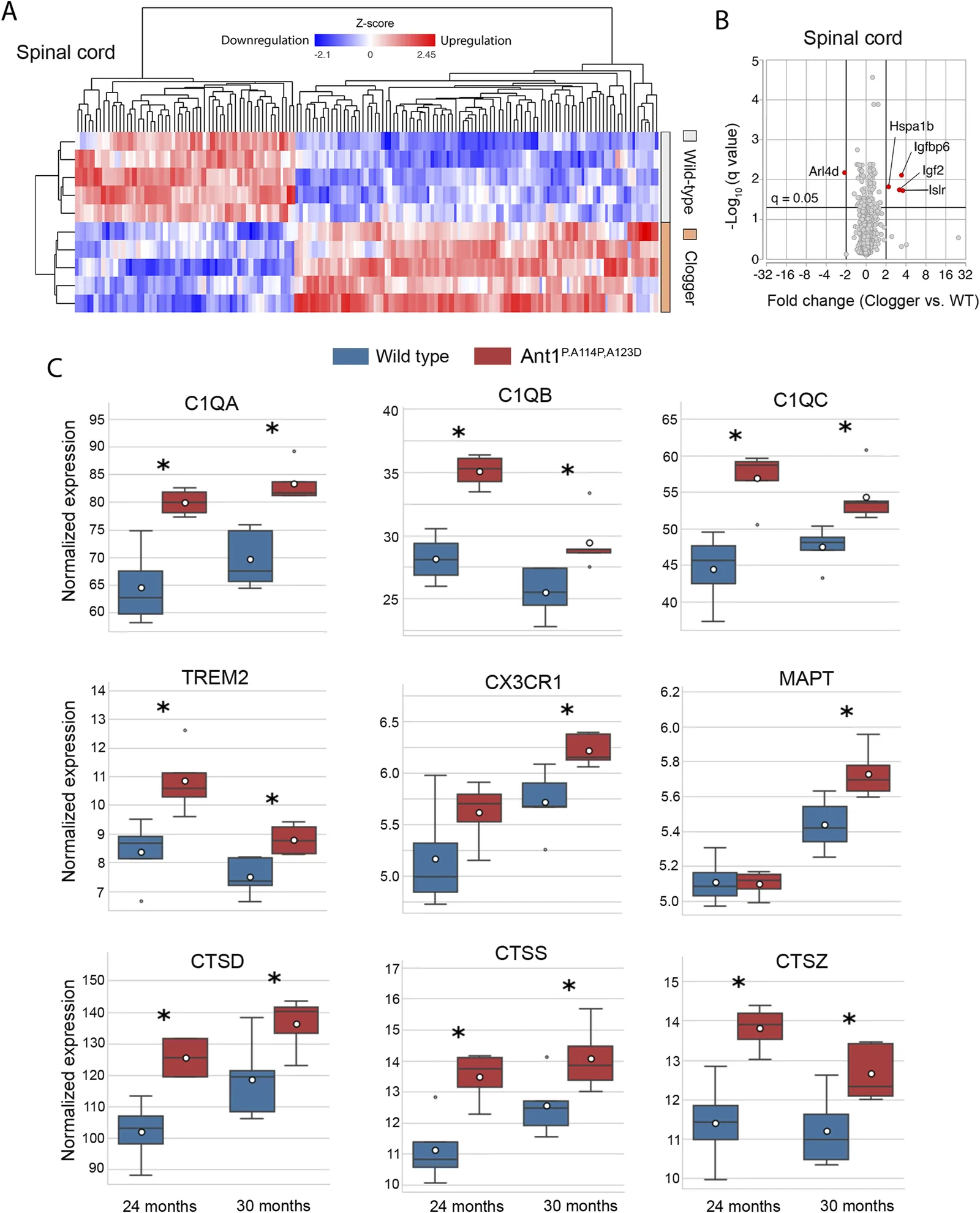
Transcriptomics analysis of neural tissues suggests cytosolic proteostatic stress in the clogger mice. **(A)** Heatmap of all genes that are differentially expressed in the spinal cord of clogger mice at 30 mo of age, compared with WT (n = 5 biological replicates per genotype, *q* < 0.05). **(B)** Volcano plot of transcriptomics from the spinal cord at 30 mo of age. **(C)** Gene set enrichment analysis revealing leading-edge genes activated in the spinal cord of clogger mice compared with the WT at 24 and 30 mo of ages (*P < 0.05).

**Figure S4.**
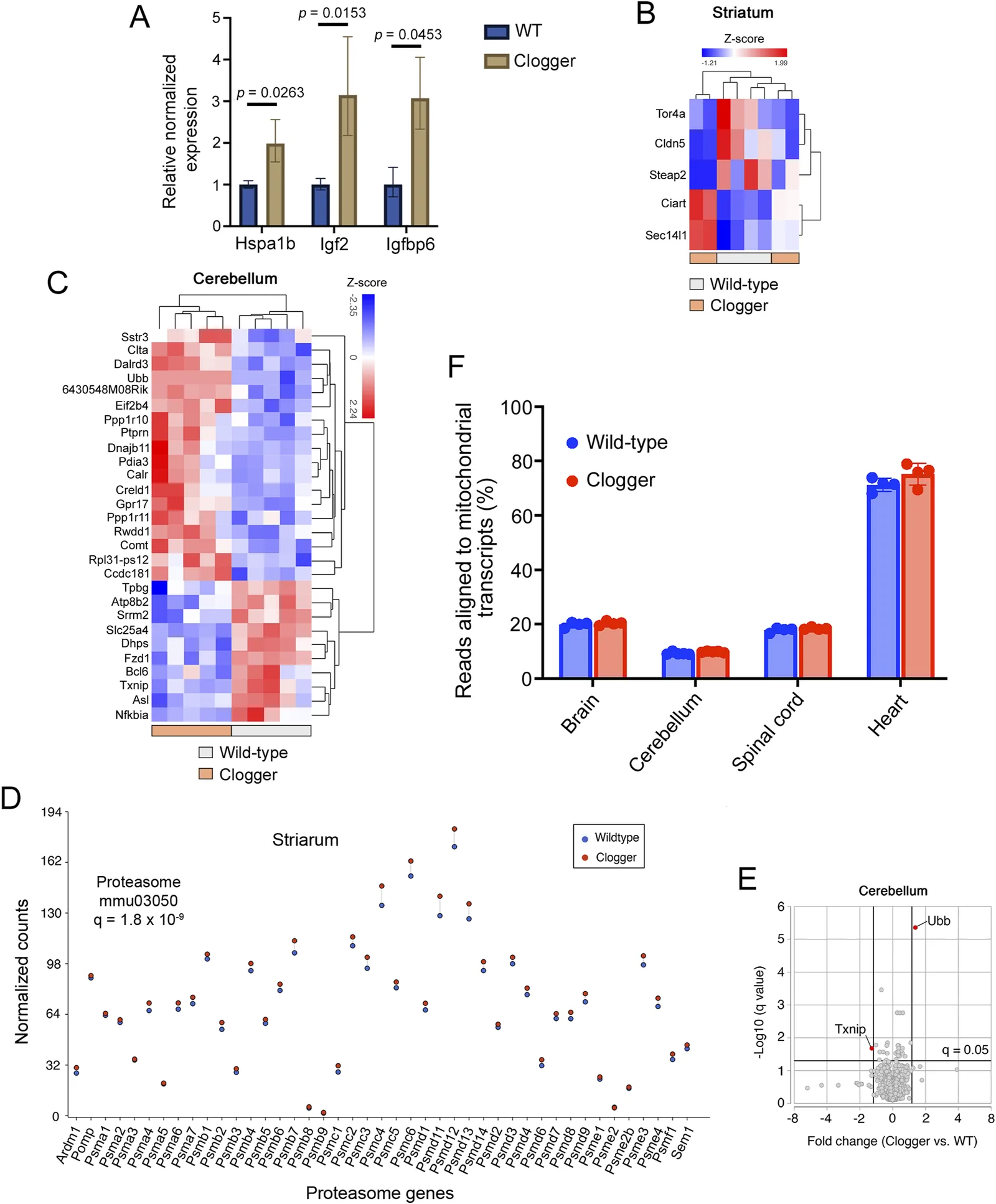
RNA-seq analysis and validation. Related to Fig 2. **(A)** RT–qPCR validating the activation of *Hspa1b*, *Igf2*, and *Igfbp6* (n = 4–5, *t* test). **(B)** Hierarchical clustering heatmap of all genes that are differentially expressed in the striatum of clogger mice, compared with WT (*q* < 0.05). **(C)** Heatmap of all genes that are differentially expressed in the cerebellum of clogger mice, compared with WT (*q* < 0.05). **(D)** Partek Pathway Analysis of transcriptomics data from the striatum at 24 mo of age suggests global proteasome up-regulation (n = 4 biological replicates per genotype). **(E)** Volcano plot of transcriptomics from the cerebellum at 30 mo of age shows significant up-regulation of *Ubb* (n = 5 biological replicates per genotype). **(F)** Comparison of mtDNA-encoded transcripts in the clogger mice compared with the WT (24–30 mo old), as represented as the percentage of total transcripts detected by RNA-seq. Note that the *Slc25a4* (*ANT1*) transcript level is consistently lower in the spinal cord (B) and cerebellum (D) from the clogger mice because of partial instability and/or reduced transcription from the mutant *Ant1*^*P.A114P,A123D*^ allele (our unpublished observation).

To learn whether Ant1^P.A114P,A123D^ induces specific stress responses in the CNS, we first analyzed the spinal cord transcriptome because the spinal cord appears to be preferentially affected in clogger mice (Coyne et al, 2023). We found a robust transcriptional signature of 149 differentially expressed genes (n = 5 biological replicates per genotype, *q* < 0.05) (Fig 2A and Table S1). Among the four most up-regulated genes were *Hspa1b*, *Igf2*, and *Igfbp6* (Fig 2B), which were validated by real-time quantitative reverse transcription PCR (RT–qPCR) (Fig S4A). *Hspa1b* encodes one of the major stress-inducible Hsp70 chaperones, which are important for protein folding and protection against proteostatic stress. Hsp70 is also involved in the stabilization and mitochondrial delivery of mitochondrial preproteins in the cytosol (Young et al, 2003). HSP70 genes have previously been shown to be transcriptionally activated by mitochondrial protein import clogging in human cells (Liu et al, 2019) and yeast (Boos et al, 2019; Coyne et al, 2023). These observations support proteostatic stress (or mPOS) in the spinal cord of *Ant1*^*P.A114P,A123D*^*/+* mice, and suggest that transcriptional up-regulation of HSP70 genes is a conserved response to mitochondrial protein import clogging.

Table S1. RNA-seq data of the spinal cord from *Ant1*^*P.A114P,A123D*^/+ compared with WT mice.

Insulin-like growth factor–binding proteins are secreted proteins that bind insulin-like growth factors to regulate their transportation, localization, and function. IGFBP-6 is unique to this family in that it has a 20- to 100-fold higher affinity for IGF2 compared with IGF1 (Bach, 1999). Thus, co-induction of *Igfbp6* and *Igf2* in clogger mouse spinal cords may be a coordinated stress response. Recent studies demonstrated that IGF2 can protect against neural damage through extracellular disposal of protein aggregates (Mellott et al, 2014; Allodi et al, 2016; Garcia-Huerta et al, 2020).

We extended our RNA-sequencing (RNA-seq) analysis to other regions within the CNS. In contrast to the spinal cord, we observed much more restricted transcriptional changes at the individual gene level in the cerebellum (n = 5 biological replicates per genotype) and striatum (n = 4 biological replicates per genotype), with only 28 and 6 differentially expressed genes, respectively (*q* < 0.05) (Fig S4B and C, Tables S2, and S3). Notably absent were any changes in genes involved in oxidative phosphorylation, consistent with a lack of mitochondrial respiratory defects. Instead, pathway analysis revealed global up-regulation of proteasomal genes in the striatum (Fig S4D), which would be predicted if protein import clogging is causing mPOS in the cytosol (Wang & Chen, 2015; Wrobel et al, 2015). We note that this is a pathway analysis and that differential expression analysis of the individual proteasomal genes was not statistically significant. Consistent with disturbed cytosolic proteostasis, we found that *Ubb* (ubiquitin B) is the most up-regulated gene in the cerebellum (Fig S4E). *UBB* transcription was previously shown to be up-regulated in neurons of PD patients (Kim et al, 2006). Related to the *Ubb* findings, we also noted significant increases in the *Dnajb11* transcript, which encodes an Hsp40 chaperone involved in unfolded protein responses and proteostasis (Table S2). Finally, we point out that there was moderately but significantly increased expression of *Comt* transcript detected in the clogger cerebellum as well, which is a gene involved in the degradation of dopamine and norepinephrine that is commonly targeted by drugs used to treat patients in the early stages of PD. Overall, these transcriptional findings are consistent with disturbed extra-mitochondrial proteostasis by Ant1^P.A114P,A123D^ expression, even though the extent of import clogging appears to be below the threshold for causing detectable OXPHOS defects. Moreover, different regions of the CNS seem to have different stress responses. Consistent with the lack of detectable respiratory deficiency, the total number of mtDNA-encoded transcripts is not significantly changed in the neural tissues of the clogger mice when compared to the WT (Fig S4F).

Table S2. RNA-seq data of the cerebellum from *Ant1*^*P.A114P,A123D*^/+ compared with WT mice.

Table S3. RNA-seq data of the striatum from *Ant1*^*P.A114P,A123D*^/+ compared with WT mice.

**Figure S5.**
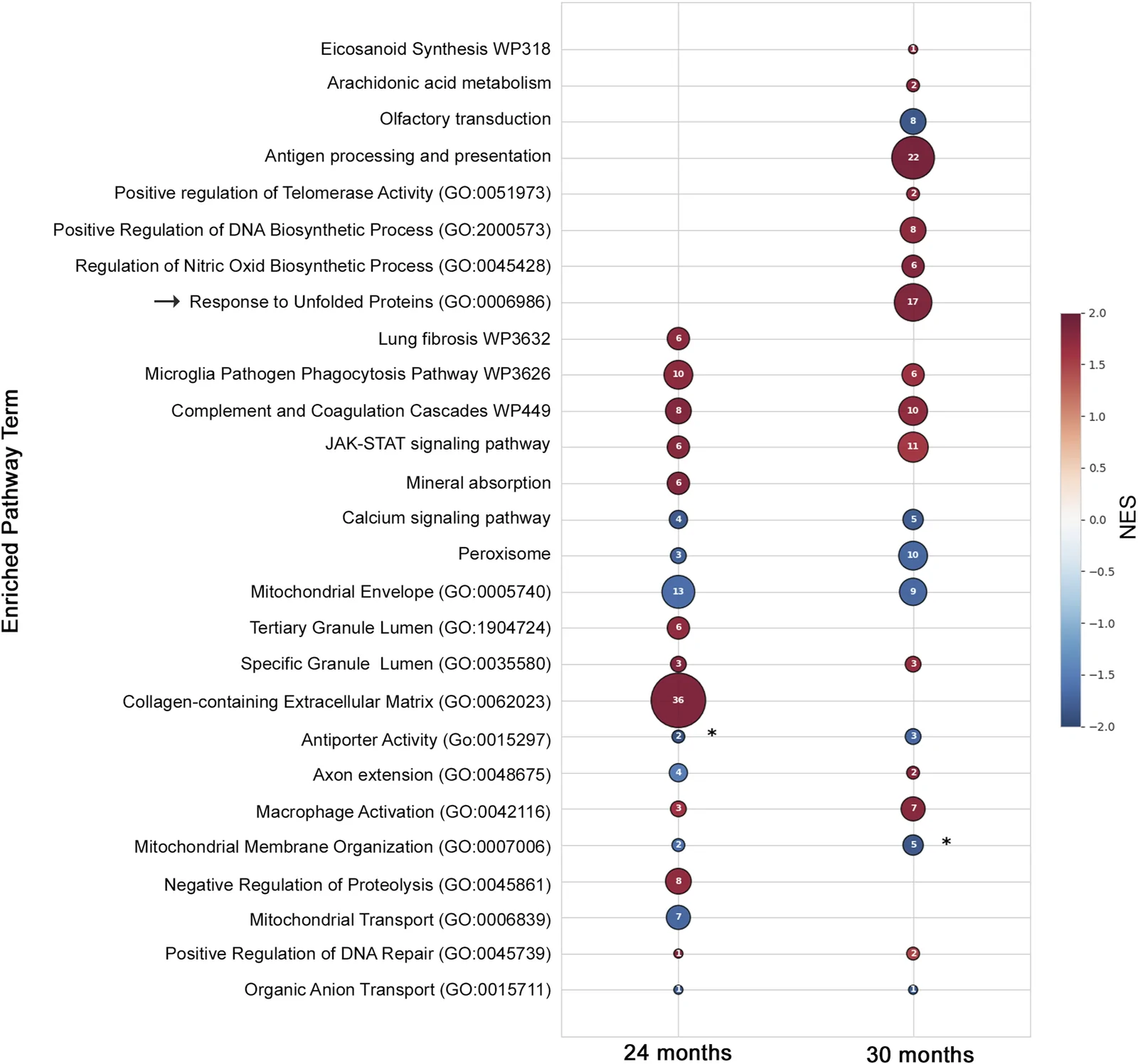
Gene set enrichment analysis of spinal cord RNA-seq data generated from 24- and 30-mo-old clogger versus WT mice. Related to Fig 2. Gene set enrichment analysis examining all the genes in every pathway to identify leading-edge genes that drive the positive or negative effect the most. The size of the leading-edge gene count is indicated in the letters and also the size of each circle. All of these pathways are nominally significant. *, significant pathways after FDR correction (*P* < 0.05).

In addition to probing for single-gene effects, gene set enrichment analysis revealed that sets of transcripts involved in microglia phagocytosis, macrophage activation, JAK-STAT signaling, and complement and coagulation cascades seemed to be activated in the spinal cord of 24- and 30-mo-old of *Ant1*^*P.A114P,A123D*^*/+* mice (Fig S5). This supports heightened inflammation in aged clogger mice. In contrast, genes encoding proteins of mitochondrial envelope and membrane organization are down-regulated, likely as a response to Ant1^P.A114P,A123D^ expression, which reduces protein loading on the mitochondrial protein import machinery. Interestingly, many genes involved in response to unfolded proteins appear to be activated only in 30-mo-old *Ant1*^*P.A114P,A123D*^*/+* mice, suggesting age-dependent aggravation of proteostatic stress.

**Figure S6.**
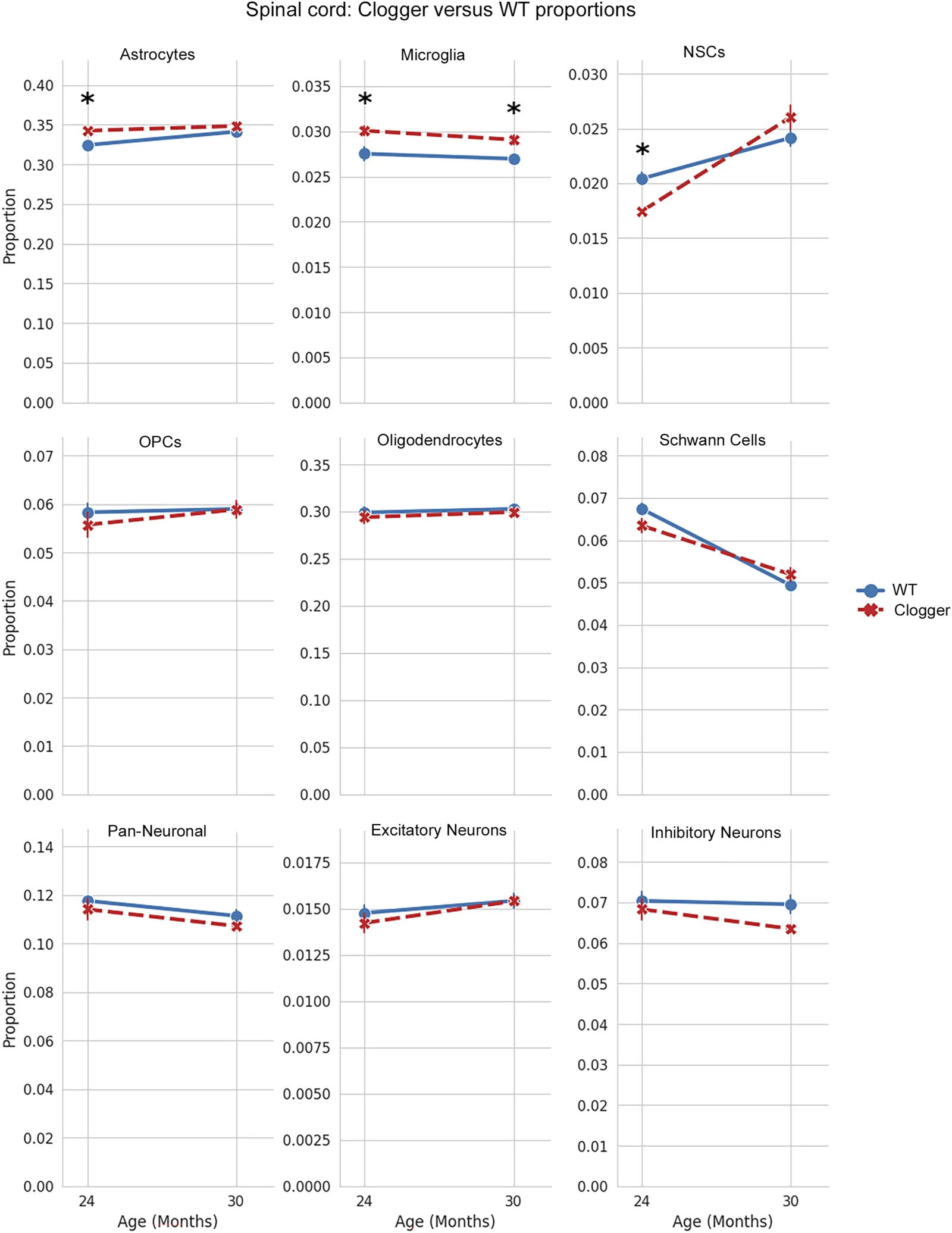
Deconvolution analysis of bulk RNA-seq data showing the proportions of neural cell types. Related to Fig 2. Spinal cord tissues from the clogger and WT mice at different ages are compared. *, *P* < 0.05 (Welch’s *t* test).

To explore whether the changes in microglial- and inflammatory-related transcripts might be due to changes in cell composition, we then deconvoluted the bulk RNA-seq data generated from spinal cord samples and found that there is a significant increase in the proportions of microglia in aged clogger mice (Fig S6). Significant increases of astrocytes were also seen at 24 but not 30 mo of age.

**Figure S7.**
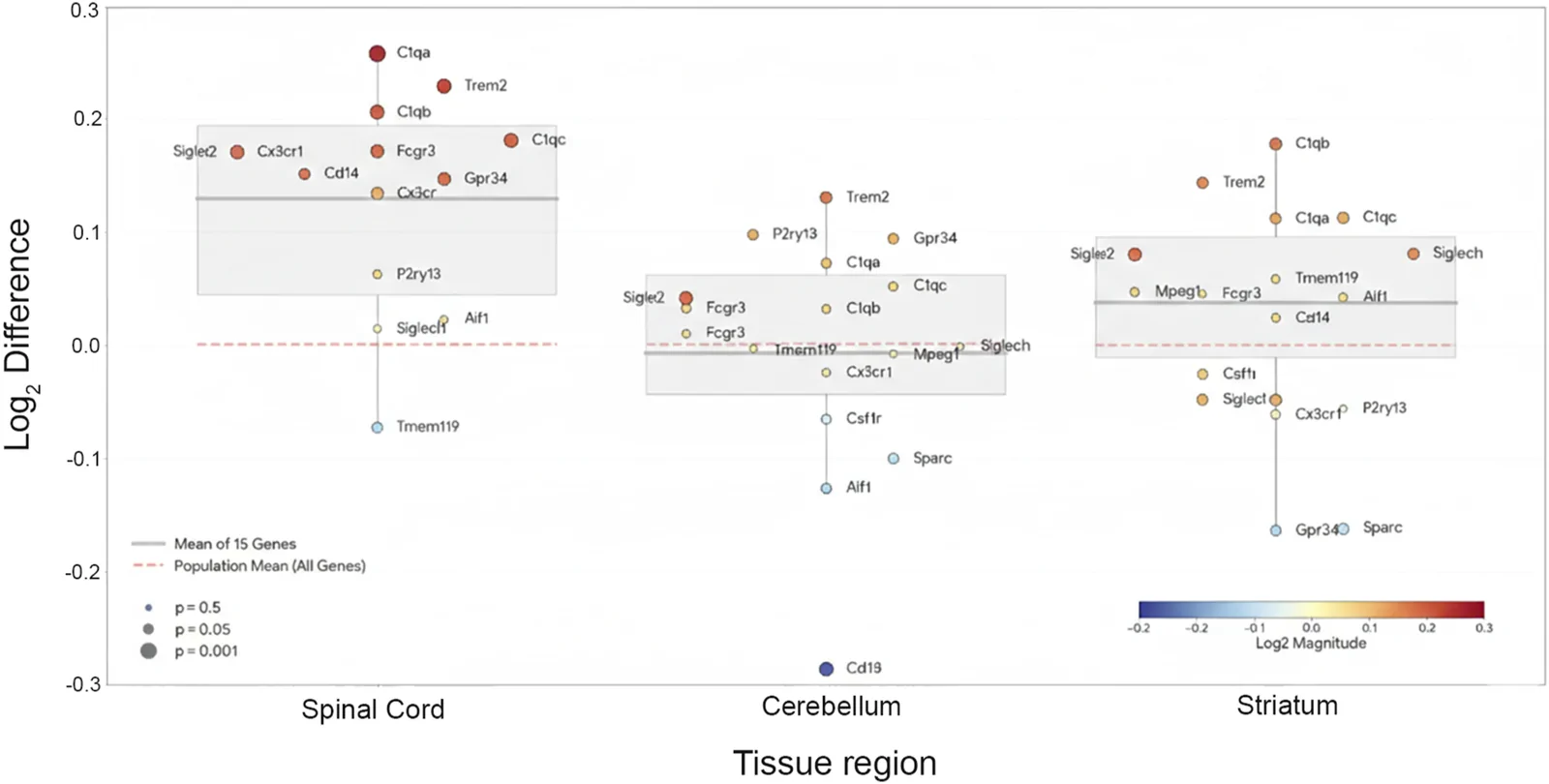
Whisker box plot of microglial gene expression changes in clogger versus WT mice. Note the increase in the spinal cord was highly significant (*P* = 0.00071), whereas the striatum showed a trend in the same direction (*P* = 0.08437). In contrast, the cerebellum did not show any overall change in the expression of microglial genes (*P* = 0.79436).

Building on the deconvolution results, we also examined the bulk transcriptome data of the clogger and WT mice to probe for expression differences in a total of 15/25 microglial-specific genes identified by single-cell analysis (Tasic et al, 2016). Overall, we found evidence of significantly increased microglial genes in the spinal cord, and a trend for increased expression in the striatum (Fig S7, Tables S4, and S5). Interestingly, the most robustly increased microglial genes in the spinal cord and striatum were *C1qa*, *C1qb*, and *C1qc* (which encode the three chains of the complement component C1q), and *Trem2* (Figs S7 and 2C). These genes are known to exhibit highly correlated increases in expression during transitions from homeostatic to activated damage-associated microglial states, particularly in the context of neurodegenerative diseases, such as Alzheimer’s disease (Walker, 2020; Lin et al, 2024). In fact, all four of these proteins are known to aggregate with β-amyloid plaques. Like *Trem2*, *Cx3cr1* and *Mapt* are activated in the spinal cord of aged WT mice, and their expression is further heightened in the aged clogger mice (Fig 2C). Expression levels of these two genes are also associated with Alzheimer’s disease (Cho et al, 2011; Allen et al, 2014; Gonzalez-Prieto et al, 2021; Frosch et al, 2025). The increased expression of these genes in clogger mouse brains suggests that Ant1^P.A114P,A123D^ expression triggers a similar transcriptional signature to β-amyloid and that microglia may not differentiate between these types of proteostatic stressors. Finally, we found that the expression of *Ctsd*, *Ctss*, and *Ctsz*, encoding cathepsins D, S, and Z, is activated in the spinal cord of *Ant1*^*P.A114P,A123D*^*/+* mice, which provides further support for increased phagocytic activities. In summary, the transcriptomics data support neuronal damage in *Ant1*^*P.A114P,A123D*^*/+* mice, which leads to microglial and astrocyte activation.

Table S4. Changes in microglial-specific RNAs between clogger and WT mice compared with all genes.

Table S5. Log_2_ expression changes of microglial RNAs in clogger versus WT mice.

### Ant1^P.A114P,A123D^ expression worsens neurodegenerative phenotype in a mouse model of PD

We next used mouse genetics to test whether modeling mild mitochondrial protein import stress with Ant1^P.A114P,A123D^ can worsen cytosolic protein aggregation and disease in an established mouse model of neurodegeneration. We crossed the clogger mice with hemizygous transgenic mice expressing the human α-syn with the A53T mutation, which is an autosomal dominant cause of familial PD and a highly aggregation-prone protein (Lee et al, 2002b). Transgenic α-syn (A53T) mice develop overt neurological dysfunction at 12–16 mo of age, which progresses to end-stage paralysis within 2–3 wk of symptom onset (Lee et al, 2002b). Thus, to detect any additive effect of Ant1^P.A114P,A123D^ expression, we characterized the mice at 8–9 mo of age (∼20 mice/sex/genotype).

**Figure 3.**
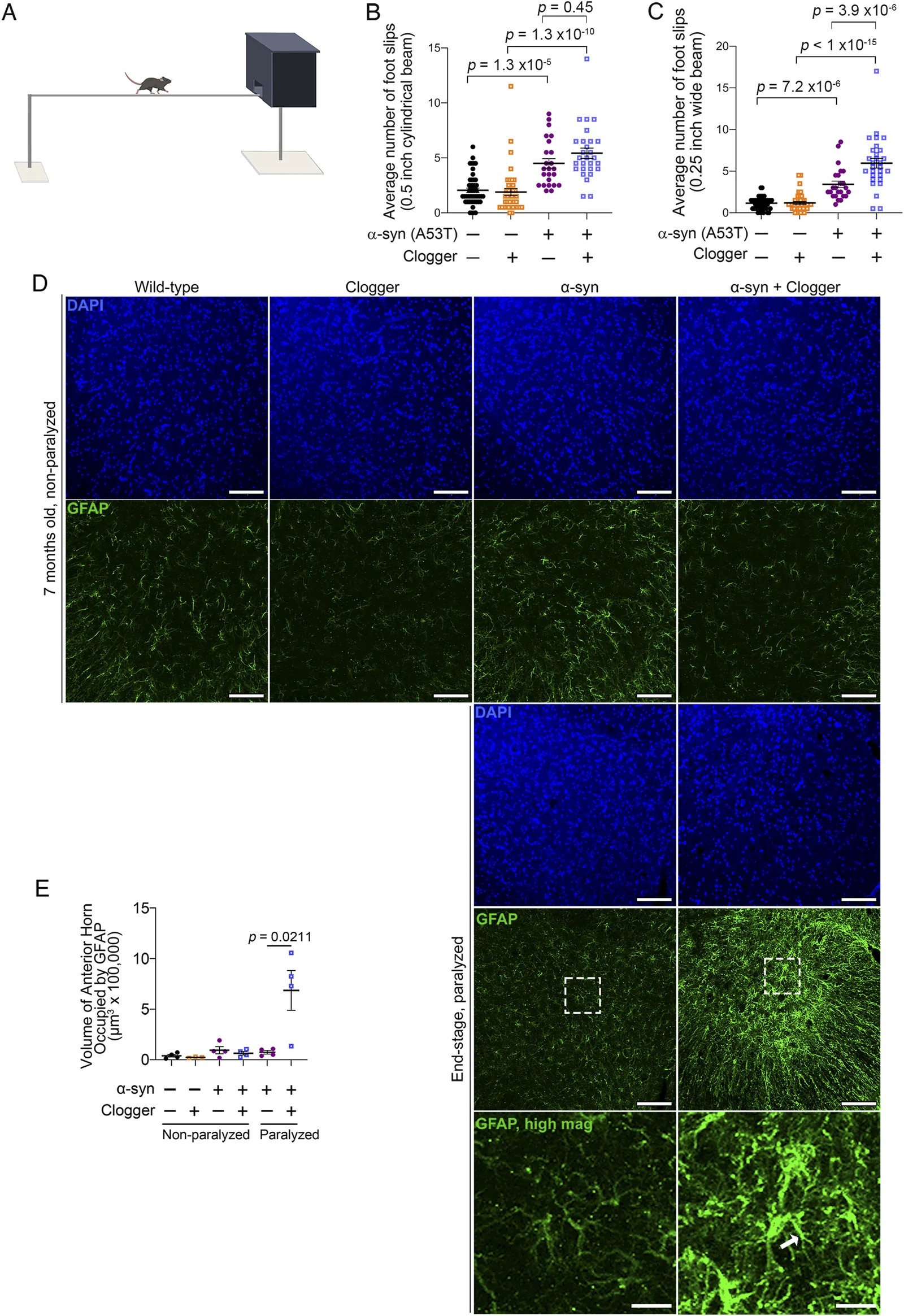
Mild protein import stress in the clogger mice impairs motor coordination and causes neuroinflammation only in the α-syn (A53T) transgenic background. **(A)** Schematic of the beam walking test. **(B)** Beam walking test suggested marginally worsened coordination on a 0.5-inch cylindrical beam in clogger + α-Syn A53T double mutant compared with α-Syn A53T single mutant mice, as judged by the number of times each mouse’s hindlimbs slipped off the beam. Data were first analyzed with a three-way ANOVA probing for effects of sex, α-syn, and clogger genotype. With no significant effect or interaction effect from sex, males and females were consolidated. Adjusted *P*-value that is shown is from a two-way ANOVA with Sidak’s multiple comparisons test. Horizontal bars indicate average values ± SEM (n = 11–28 mice/sex/genotype, 8–9 mo of age). **(C)** Beam walking test suggested severely impaired coordination on a 0.25-inch rectangular beam in clogger + α-Syn A53T double mutant compared with α-Syn A53T single mutant mice. Data were analyzed as in (B). There was a significant interaction between α-syn (A53T) and clogger genotypes (*P* = 7.5 × 10^−5^). Adjusted *P*-value that is shown is from a two-way ANOVA with Sidak’s multiple comparisons test. Horizontal bars indicate average values ± SEM (n = 11–28 mice/sex/genotype, 8–9 mo of age). **(D)** Representative images from immunofluorescence for the GFAP in the ventral horns of the lumbar spinal cord from control (WT, clogger) and end-stage α-syn mice with or without protein import clogging. The white arrows show the staining of GFAP in representative astrocytes. Scale bar = 100 microns but 20 microns for the high-magnification images. **(E)** Increased volume occupied by GFAP in double mutant mice. Aggregate data for GFAP signal were generated exclusively from the ventral horns of five to eight sections per mouse at anatomically distinct lumbar spinal cord levels from three to four biological replicates per genotype. End-stage mice were 11.5–14.76 mo of age. Nonparalyzed mice were 7 mo of age ±2 wk. *P*-values were derived from a *t* test. Horizontal bars in (E) indicate average values ± SEM. GFAP, glial fibrillary acidic protein.

Motor symptoms such as bradykinesia and postural instability are hallmarks of PD. We first assessed balance and coordination using the beam walking test. Mice were trained to walk across a thin beam, and the number of times their paws slipped off the beam was scored as a measure of motor coordination (Fig 3A). As expected, α-syn mice slipped more often on both a 0.5-inch-diameter cylindrical beam compared with WT mice (*P* = 1.3 × 10^−5^) and a 0.25-inch rectangular beam (*P* = 7.2 × 10^−6^) (Fig 3B and C). Ant1^P.A114P,A123D^ expression significantly increased the number of foot slips on the smaller beam (two-way ANOVA *Ant1* x α-syn genotype interaction effect, *P* = 0.0007, Sidak’s multiple comparisons test, *P* = 3.9 × 10^−6^), while showing a trend for further impairing performance on the larger beam (two-way ANOVA Ant1 x α-syn genotype interaction effect, *P* = 0.12, Sidak’s multiple comparisons test, *P* = 0.45). Importantly, Ant1^P.A114P,A123D^ expression had no effect in the WT background (Fig 3B and C). We note that sexes were consolidated for analysis as initial three-way ANOVA did not show a sex main effect (*P* = 0.53) or interaction effects (*Ant1* genotype x sex, *P* = 0.48; α-syn genotype x sex, *P* = 0.56). These data suggest that Ant1^P.A114P,A123D^ and α-syn (A53T) expression acts synergistically to impair motor function.

**Figure S8.**
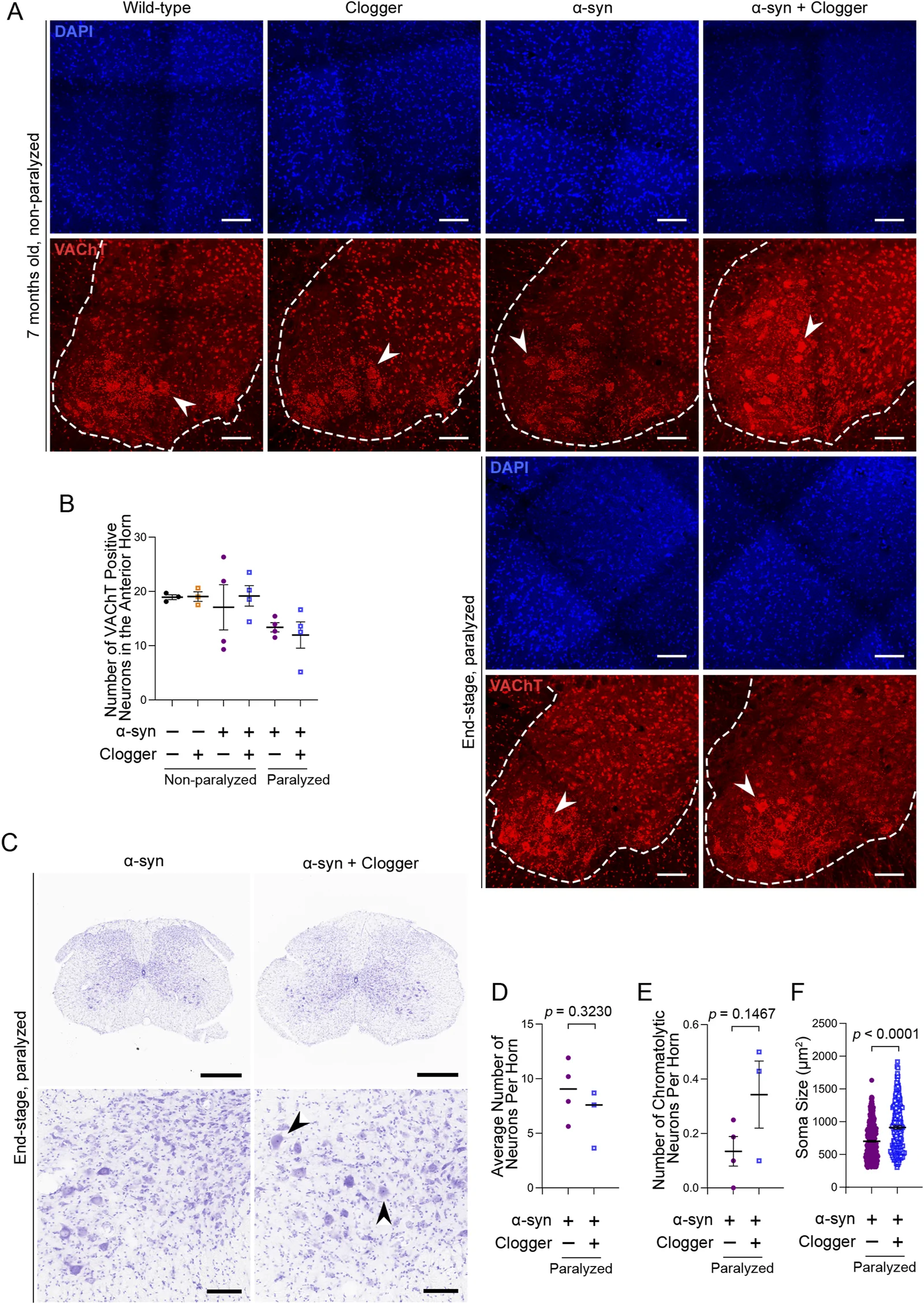
Histological analysis of motor neurons in the lumbar spinal cord. Related to Fig 3. **(A)** Representative images from control (WT, clogger) and end-stage α-syn mice with or without protein import clogging, probed for the VAChT. Scale bar = 100 mm. **(B)** Number of VAChT-positive motor neurons in the anterior horn. There were no significant changes between groups as per ANOVA testing with Holm–Sidak’s multiple comparisons test. **(C)** Representative images of Nissl-stained lumbar spinal cords from end-stage α-syn mice with or without protein import clogging. Top: whole section, scale bar = 500 mm. Bottom: magnified view corresponding to anterior horn motor neurons, scale bar = 100 mm. Arrowheads represent chromatolytic neurons. **(D, E, F)** Quantification of Nissl stain in lumbar spinal cord sections. **(D)** Average number of motor neurons counted per anterior horn. **(E)** Average number of atrophic (chromatolytic) motor neurons counted per anterior horn. **(F)** Comparison of soma sizes. Aggregate data for VAChT immunofluorescence and Nissl staining were generated from the ventral horns of two to eight sections per mouse at anatomically distinct lumbar spinal cord levels from three to four biological replicates per genotype. End-stage mice were 11.51–14.75 mo of age. Nonparalyzed mice were 7 mo of age ±2 wk. *P*-values were derived from a *t* test. Horizontal bars in (B) and (D, E, F) indicate average values ± SEM. VAChT, vesicular acetylcholine transporter.

We next assessed potential neuroanatomic etiologies of the observed phenotypes. We primarily focused on spinal cord motor neurons, because these neurons are among the earliest cells affected in α-syn A53T mice (Van Der Putten et al, 2000). In end-stage α-syn mice, we did not observe a statistically significant decrease in the number of vesicular acetylcholine transporter–positive motor neurons in the lumbar spinal cord (Fig S8A and B). Nissl staining of end-stage mice showed a trend toward reduced number of anterior horn neurons and increased number of chromatolytic neurons in the double mutant mice (Fig S8C–E). Consistent with increased chromatolysis, the soma size of the anterior horn neurons in the end-stage double mutant was significantly larger than that of the α-syn mice (Fig S8F; Bodian & Mellors, 1945). Importantly, we observed an increase in neuroinflammation in the end-stage double mutant mice compared with α-syn only, as judged by GFAP staining (Fig 3D and E). Taken together, these data support increased neuronal damage in the spinal cord of end-stage double mutant mice compared with α-syn single mutant animals.

**Figure S9.**
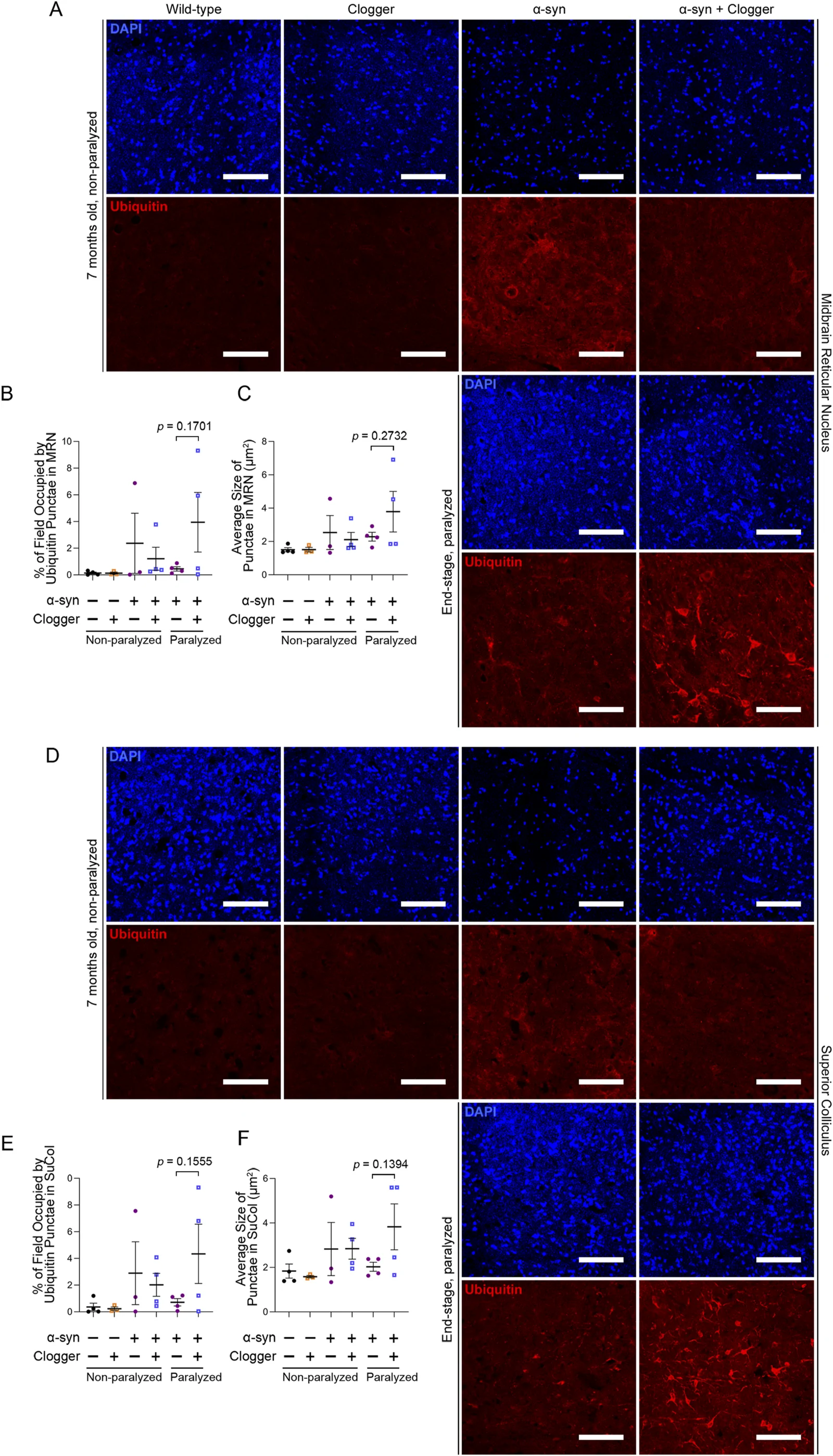
Ubiquitin inclusion bodies in midbrain regions. Related to Fig 3. **(A, D)** Representative images from control (WT, clogger) and end-stage α-syn mice with or without protein import clogging for the formation of ubiquitin-positive inclusion bodies in the midbrain reticular nucleus (A) and superior colliculus (D). Scale bar = 100 µm. **(B, C, E, F)** Quantification of ubiquitin percent occupancy and size within the midbrain reticular nucleus (B, C) and the superior colliculus (E, F). Aggregate data were generated exclusively from similar portions of the SNc of two to four sections per mouse at anatomically distinct levels of the midbrain from three to four biological replicates per genotype. End-stage mice were 12–14.76 mo of age. Nonparalyzed mice were 7 mo of age ±2 wk. *P*-values were derived from a *t* test. Horizontal bars in (B, C, E, and F) indicate average values ± SEM. SNc, substantia nigra pars compacta.

Ubiquitin-positive inclusion bodies are present in the midbrain regions in this particular mouse model overexpressing human α-syn A53T (Lee et al, 2002a). We therefore assessed for the formation of ubiquitin inclusion bodies within the midbrain regions, to see whether clogging further challenges cytosolic proteostasis and promotes inclusion formation in the α-syn background. Within the midbrain reticular nucleus and the superior colliculus (areas responsible for movement initiation in the reticulospinal, tectospinal, and oculomotor systems), we observed a mild but insignificant trend toward increased inclusion body formation in α-syn + Ant1^P.A114P,A123D^ mice when compared to single mutant α-syn mice (Fig S9A–F).

**Figure S10.**
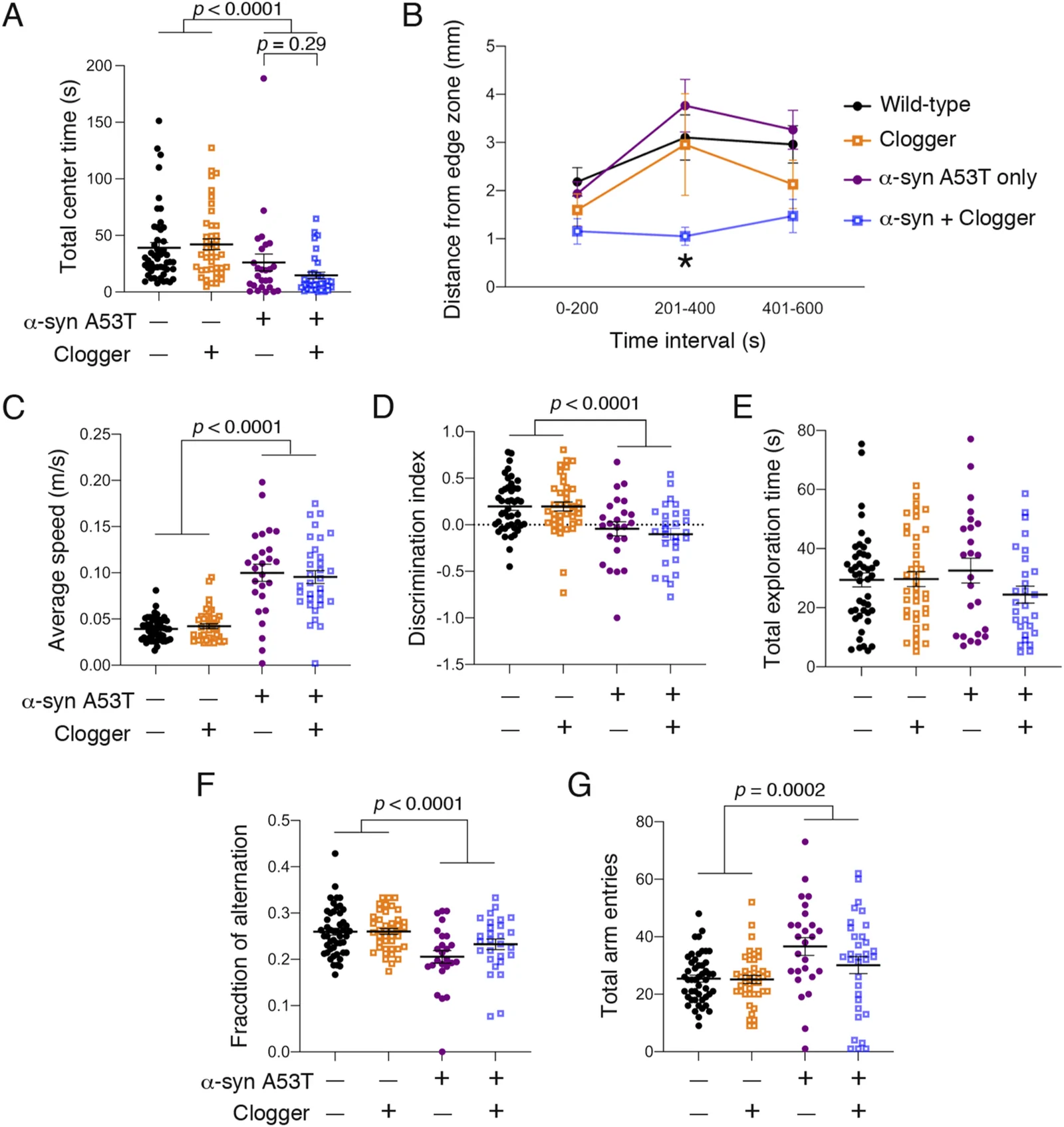
Assessment of anxiety-like behavior, spontaneous locomotion, and memory in WT, clogger, α-syn, and double mutant mice. Related to Fig 3. **(A)** α-Syn^A53T^ expression significantly increases anxiety-like behavior, as indicated by spending less time in the center zone in the open field test. Although clogger protein expression reduced the average time spent in the center zone compared with in α-syn transgenics, this was not statistically significant (n = 11–29/sex/genotype). Each data point is a biological replicate. **(B)** Open field test data were divided into 200-s epochs, and the average distance from the edge zone while in the center zone was plotted. This showed that double mutant mice tend to stay closest to the edge zone, particularly in the middle epoch. Data were represented as average values ± SEM and were analyzed using a two-way repeated-measures ANOVA with epoch as the within-subjects variable and α-syn and clogger genotypes as the between-subjects variables, with Geisser–Greenhouse correction for violation of sphericity. A significant interaction effect was detected (epoch x α-syn x clogger, *P* = 0.042). Dunnett’s multiple comparisons test detected a significant reduction in the average distance from the edge zone for double mutant mice in the middle epoch (*P* = 0.017, shown as *) (n = 11–29/sex/genotype). **(C)** α-Syn^A53T^ expression significantly increases the average speed of spontaneous locomotion in the open field test, which was not affected by the clogger protein. Each data point is a biological replicate (n = 11–29/sex/genotype). **(D)** α-Syn^A53T^ expression significantly impairs long-term object memory in the novel object recognition test, which was not affected by the clogger protein. Each data point is a biological replicate (n = 11–27/sex/genotype). For better visualization, the discrimination index was plotted, which is ((time with new object) – (time with old object))/(total object exploration time). **(E)** α-Syn^A53T^ expression nor clogger protein expression affected total object exploration time on the probe trial of the novel object recognition test. Each data point is a biological replicate (n = 11–27/sex/genotype). **(F)** α-Syn^A53T^ expression significantly impairs working spatial memory, as suggested by reduced Fraction of Alternation in the Y-maze. This effect was not significantly affected by clogger protein expression. Each data point is a biological replicate (n = 25–48/sex/genotype). **(G)** α-Syn^A53T^ expression significantly increases the total number of arm entries in the Y-maze spontaneous alternation test, which is not affected by clogger protein expression. Each data point is a biological replicate (n = 25–48/genotype). All data in this figure were generated from 8- to 9-mo-old mice. Data were first analyzed with three-way ANOVAs probing for effects of sex, α-syn^A53T^, and clogger genotype. With no significant effect or interaction effect from sex, males and females were consolidated, and two-way ANOVAs were performed to simplify post hoc analyses. For (A), (C), (D), (F), and (G), two-way ANOVA showed a significant main effect of α-syn^A53T^ expression, as indicated. Horizontal bars indicate average values ± SEM.

Does Ant1^P.A114P,A123D^ expression aggravate additional phenotypes of α-syn mice? Up to 40% of PD patients experience anxiety (Walsh & Bennett, 2001). We found that mutant α-syn expression significantly increased anxiety-like behavior in the open field test (*P* = 1.3 × 10^−4^) (Fig S10A). While in the center zone, double mutant mice remained closest to the edge zone on average, suggesting Ant1^P.A114P,A123D^ expression moderately increases thigmotaxis or anxiety-like behavior in α-syn, but not WT, mice (Fig S10B). Spontaneous locomotor activity was increased in α-syn mice (*P* < 10^−15^), as previously reported (Unger et al, 2006), which was not affected by Ant1^P.A114P,A123D^ (Fig S10C). In the novel object recognition test, α-syn expression caused a significant defect in long-term object memory (*P* = 2 × 10^−6^), with no effect from Ant1^P.A114P,A123D^ expression (Fig S10D). The total time spent exploring either object was largely unaffected by genotype (Fig S10E). In the Y-maze spontaneous alternation test, α-syn expression caused a significant defect in short-term spatial memory (*P* = 2.5 × 10^−5^), with no effect of Ant1^P.A114P,A123D^ expression (Fig S10F). The total number of arm entries in the Y-maze was increased by α-syn expression (*P* = 1.6 × 10^−4^), with no effect of Ant1^P.A114P,A123D^ expression (Fig S10G). It is likely that the memory defects in α-syn mice are already severe by 9 mo old, precluding detection of any potential additive effect from Ant1^P.A114P,A123D^ expression. We note that for each of the above analyses a three-way ANOVA was first performed to probe for an effect of sex (sex main effect for time in center zone on open field test, *P* = 0.27; for average speed, *P* = 0.67; for discrimination index in novel object recognition test, *P* = 0.95; for total exploration time in novel object recognition test, *P* = 0.79; for Fraction of Alternation in Y-maze, *P* = 0.57; for total arm entries in Y-maze, *P* = 0.57). No significant effect from sex was observed, so sexes were consolidated and a two-way ANOVA was performed.

**Figure S11.**
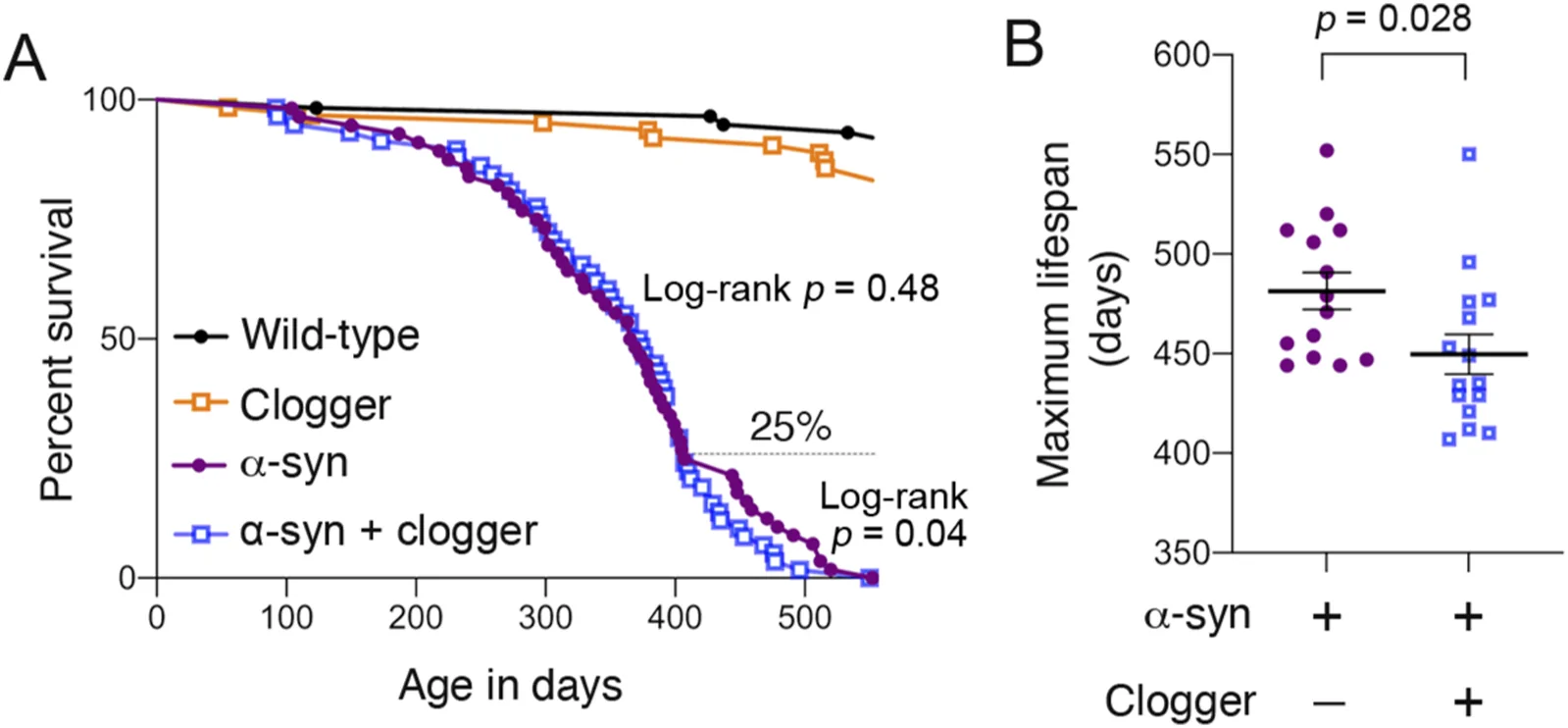
Lifespan analyses of WT, clogger, α-syn, and double mutant mice. Related to Fig 3. **(A)** Lifespan analysis of WT, clogger, α-syn, and double mutant mice (n = 56–63/genotype). The 25% longest-lived mice depicted in (E) are noted on the graph. **(B)** Maximum lifespan analysis of the 25% longest-lived mice suggests modestly reduced maximal lifespan in double mutant mice. Horizontal bars indicate average values ± SEM. Data were analyzed by an unpaired *t* test. Each data point is a biological replicate (n = 14–15/genotype).

Finally, we followed the mice through end-stage paralysis to determine whether Ant1^P.A114P,A123D^ expression reduced lifespan in α-syn mice (n = 56–63 mice/genotype). We found no change in median lifespan in double mutant mice compared with α-syn alone (Fig S11A). Intriguingly, we noticed that in the longest-lived mice (upper quartile), Ant1^P.A114P,A123D^ expression significantly reduced maximum lifespan (log-rank test, *P* = 0.04 [Fig S11A]; *t* test, *P* = 0.028 [Fig S11B]). The data show that Ant1^P.A114P,A123D^ expression may modestly shorten maximum lifespan in a mouse model of α-syn (A53T)–induced PD.

### Mitochondrial bioenergetics is unaffected in α-syn single and α-syn Ant1^P.A114P,A123D^ double mutant mice

**Figure 4.**
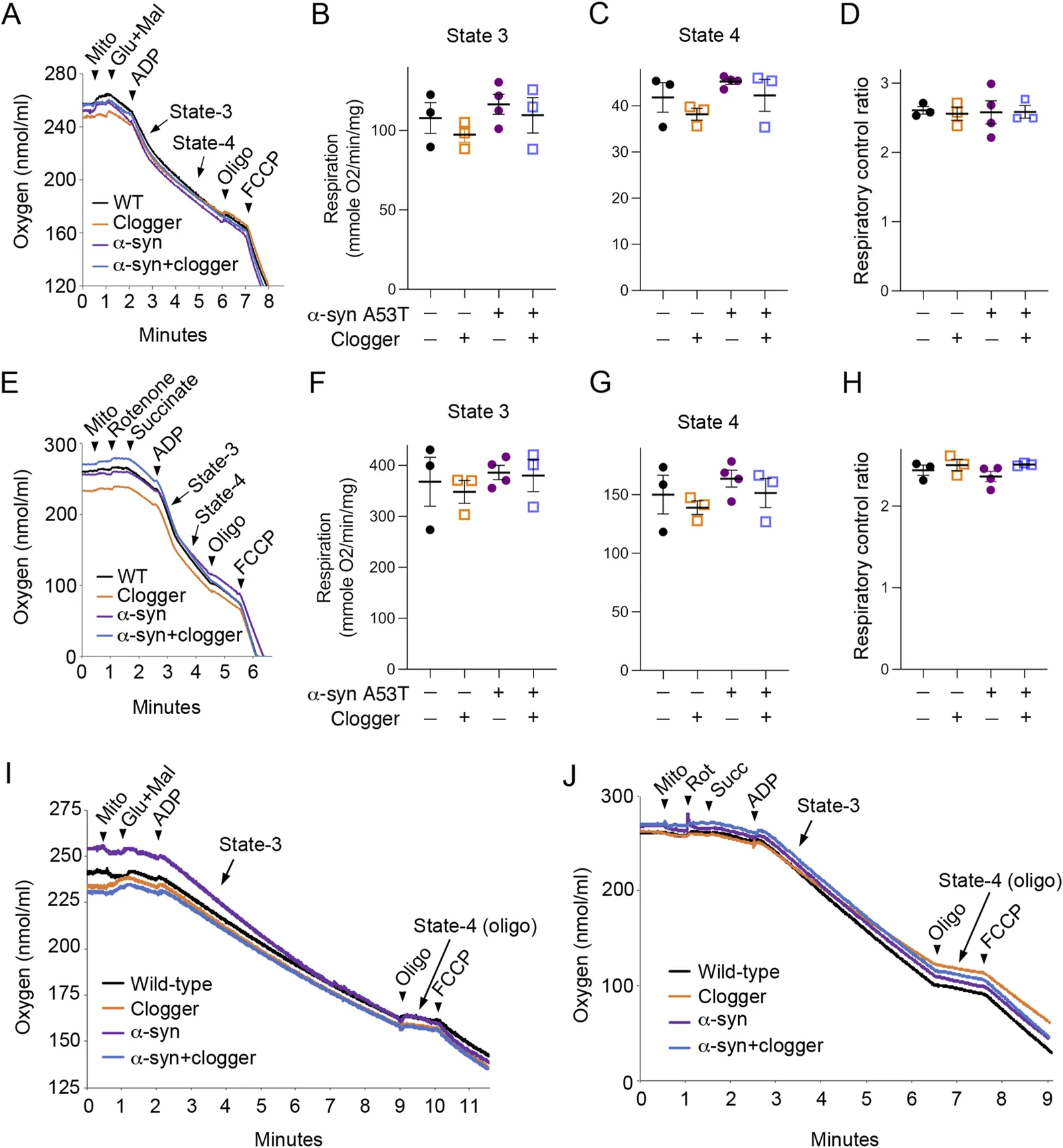
There is no detectable respiratory deficit in 9-mo-old α-syn or α-syn plus clogger double mutant mice. **(A, B, C, D)** Respirometry of purified brain mitochondria with complex I stimulated by glutamate (glu) and malate (mal). Two measurements were taken per mouse, the average of which is shown as a data point in (B, C, D). FCCP, trifluoromethoxy carbonylcyanide phenylhydrazone; Oligo, oligomycin; Glu, glutamate; Mal, malate. State 3 respiration is the maximal respiratory rate after addition of ADP. State 4 respiration is the respiratory rate after depletion of ADP. The respiratory control ratio is State 3 divided by State 4 respiratory rates. Decreased respiratory control ratio would be interpreted as increased mitochondrial damage. Two measurements were performed per mouse. Each dot represents the average value from each mouse (n = 3–4/mice/genotype, 9 mo of age ±2 wk). **(E, F, G, H)** Respirometry of purified brain mitochondria with complex II stimulated by succinate (glu) and complex I inhibited by rotenone. Each dot represents the average value from each mouse (n = 3–4 mice/genotype, 9 mo of age ±2 wk). Horizontal bars indicate average values ± SEM. **(I)** Respirometry from synaptosomal fractions with complex I stimulated as above. **(J)** Respirometry from synaptosomal fractions with complex II stimulated as above.

**Figure S12.**
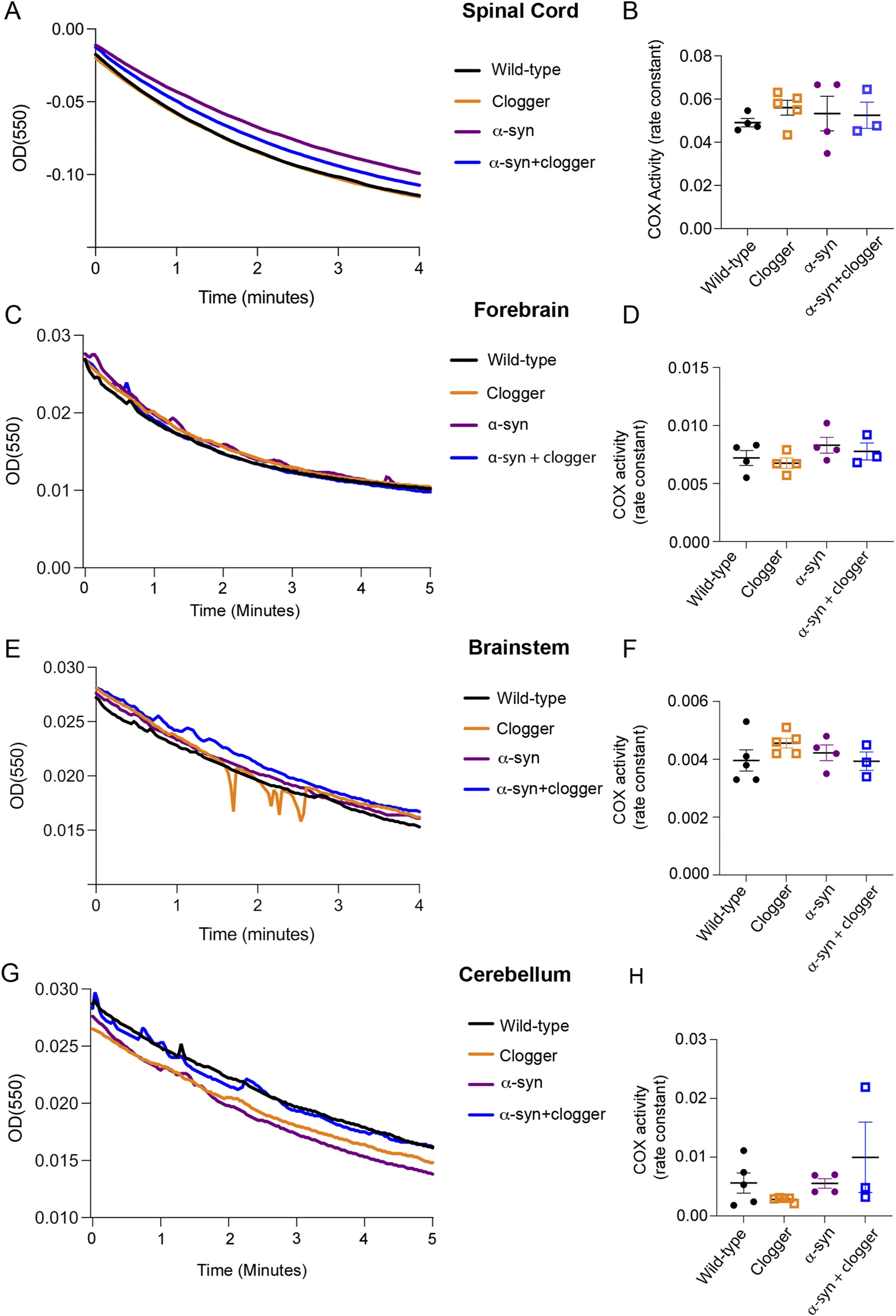
Spectrophotometric assay showing no significant difference in COX activity in the spinal cord (A, B) and forebrain (C, D), brainstem (E, F), and cerebellum (G, H) of 9-mo-old mice. Related to Fig 4. **(A, C, E, G)** Spectrophotometric traces from assessment of complex IV activity from crude mitochondrial fractions isolated from the indicated regions of the CNS. **(B, D, F, H)** Quantitation of biological replicates of the complex IV activity rate constants determined from (A), (B), (C), and (G), respectively. Each dot represents an independent biological replicate (n = 3–5/genotype). Horizontal bars indicate average values ± SEM. All data in this figure were generated from 9-mo-old mice ±2 wk. Source data are available for this figure.

There are substantial data to suggest that α-syn and other protein aggregates can impact mitochondrial bioenergetics, whereas other studies failed to generate consistent results (Kawamata & Manfredi, 2017). We tested the possibility that a synergistic defect in mitochondrial respiratory function may contribute to the enhanced double mutant phenotypes. We directly measured the respiratory capacity of isolated mitochondria. Furthermore, we focused our analysis on 9-mo-old asymptomatic mice, as this may avoid the onset of large neural tissue lesions in end-stage mice that may indirectly affect mitochondrial function and confound the interpretation of the data. Interestingly, we found that α-syn (A53T) expression did not reduce respiratory rates at the asymptomatic stage. More importantly, no respiratory deficiency was observed even in the α-syn + Ant1^P.A114P,A123D^ double mutant mice (Fig 4A–H). Because Percoll-purified mitochondria are mostly nonsynaptosomal, and α-syn primarily localizes to presynaptic sites, we also tested respiration from the synaptosomal brain fractions. These fractions are impure, containing microsomal membranes and myelin. Respirometry from synaptosomal fractions suggested that WT, α-syn (A53T), and double mutant mice all have similar respiratory rates (Fig 4I and J). To assess the possibility that regional differences in mitochondrial function were masked in the whole-brain bioenergetic experiments, we assessed cytochrome *c* oxidase (COX) activity (Fig S12) and respiratory chain protein levels (Fig S1A–H) from crude mitochondrial fractions isolated from various brain regions and the spinal cord. The data did not detect changes in COX activity or respiratory chain protein levels of either single or double mice at 9 mo of age. This contrasts with previous work showing that COX activity was reduced in the spinal cord of end-stage, paralyzed α-syn mice (Martin et al, 2006). We note that our bioenergetic experiments were performed at 9 mo of age, the age at which we observe motor defects but before paralysis onset. Taken together, it is unlikely that bioenergetic defects underlie the synergistic effect between Ant1^P.A114P,A123D^ and α-syn expression that aggravates motor deficits and neurodegenerative phenotype.

### Ant1^P.A114P,A123D^ expression increases the size of phosphorylated α-syn aggregates

**Figure S13.**
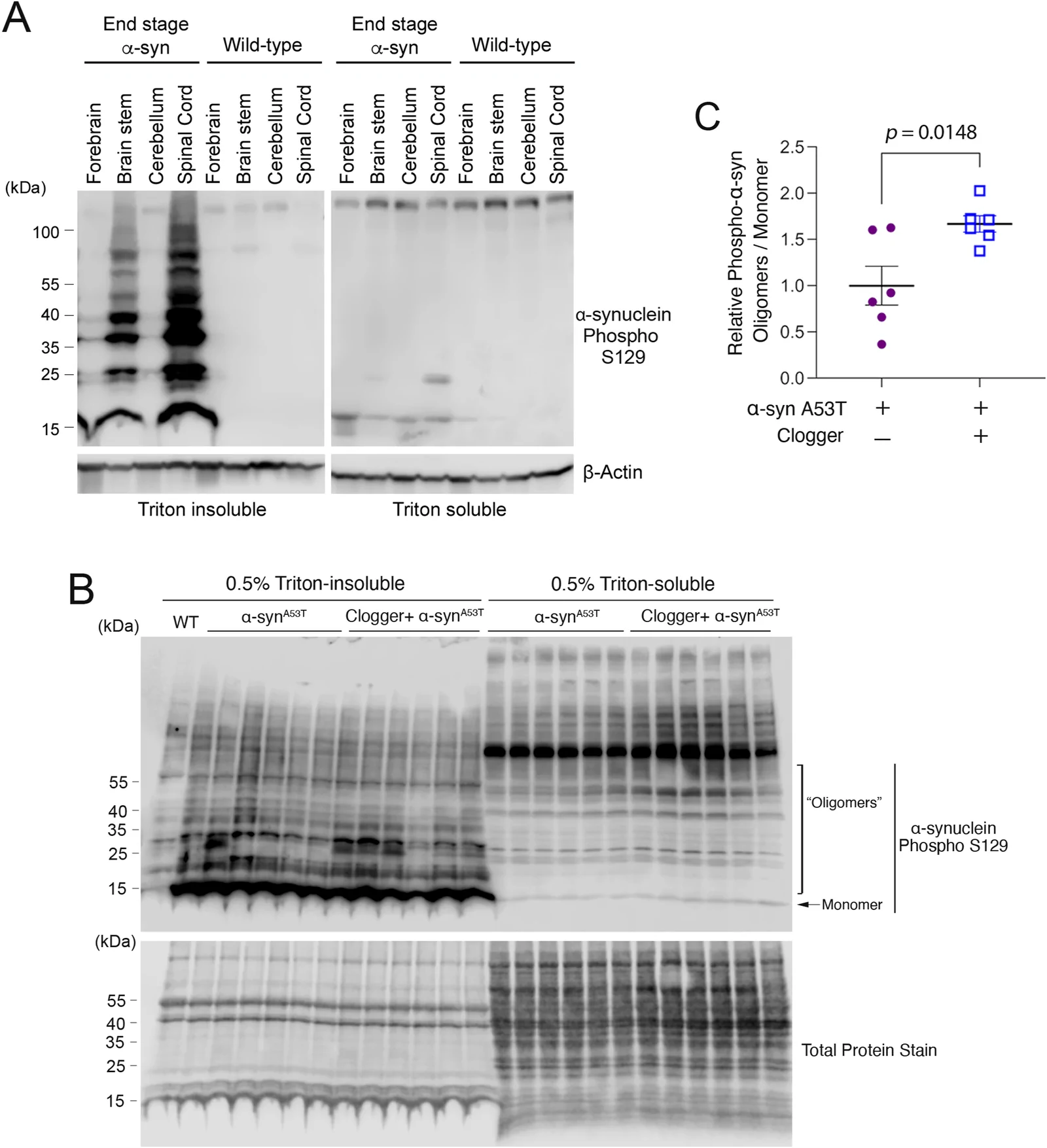
Phosphorylated α-syn is preferentially insoluble in the spinal cord of end-stage transgenic α-syn (A53T) mice, and may oligomerize preferentially in double mutant mice. Related to Fig 5. **(A)** Immunoblot analysis of CNS tissues solubilized with 0.5% Triton to assess the tissue distribution of aggregated α-syn. Antibody is specific to P-α-syn. **(B)** Immunoblot analysis of Triton-soluble and Triton-insoluble fractions using an antibody specific to P-α-syn. Each lane represents an individual mouse. **(C)** Quantification of (B) showing the oligomer/monomer ratio of P-α-syn in Triton-insoluble fractions. Source data are available for this figure.

We hypothesized that mitochondrial protein import clogging would cause mPOS and increase proteostatic burden in the cytosol, which may in turn affect α-syn aggregation. To narrow our focus to a particular region of the CNS, we performed detergent solubility studies to assess where α-syn (A53T) is preferentially detergent-insoluble, which we use as a proxy for aggregation. We probed the Triton-insoluble fractions from the forebrain, midbrain, cerebellum, and spinal cords of end-stage α-syn mice for α-syn phosphorylated at serine 129 (P-α-syn). P-α-syn accumulates in pathological aggregates and is a more specific marker for protein aggregation and disease activity compared with nonphosphorylated α-syn (Sato et al, 2013). We found that insoluble P-α-syn preferentially accumulates in the spinal cord (Fig S13A), consistent with previous studies (Lee et al, 2002b). We therefore focused our biochemical studies of protein aggregation on the spinal cord.

**Figure 5.**
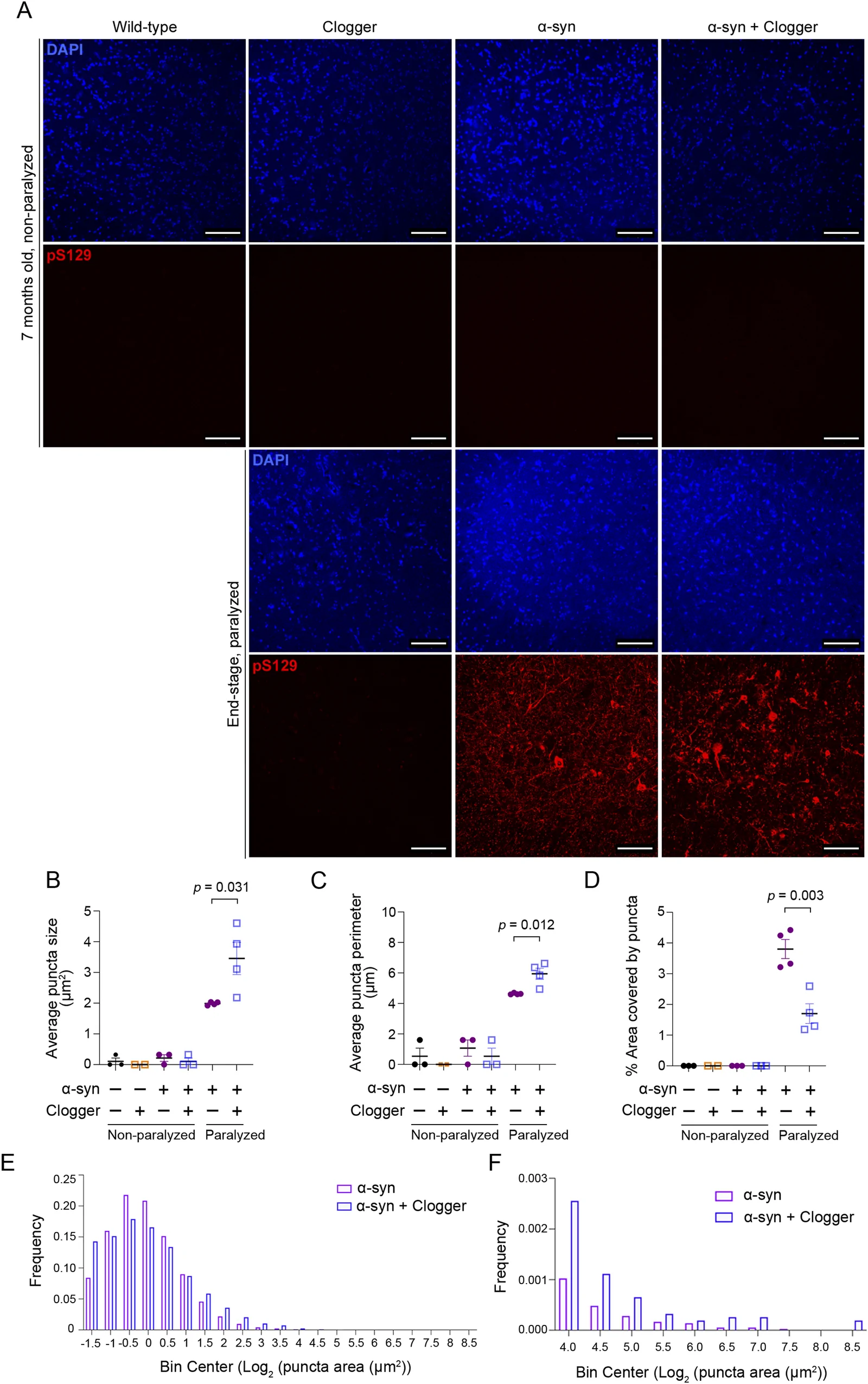
Increased size of α-syn phospho-S129 puncta in double mutant mice. **(A)** Representative images from immunofluorescence for P-α-syn in the ventral horns of the lumbar spinal cord from control (WT, clogger) and end-stage α-syn mice with or without protein import clogging. Scale bar = 100 microns. **(B)** Increased average P-α-syn punctum size in double mutant mice. **(C)** Increased average P-α-syn punctum perimeter in double mutant mice. **(D)** Reduced % area covered by P-α-syn puncta in double mutant mice. **(E)** Frequency distribution of P-α-syn punctum size. **(F)** Frequency distribution as in (E), but focused on larger puncta that are not visible in (E). Aggregate data were generated exclusively from the ventral horns of three sections per mouse at anatomically distinct lumbar spinal cord levels from two to four biological replicates per genotype. End-stage mice were 12–14.75 mo of age. Nonparalyzed mice were 7 mo of age ±2 wk. *P*-values were derived from a *t* test. Horizontal bars in (B–D) indicate average values ± SEM.

First, immunoblot analysis showed that P-α-syn has a slightly increased oligomer/monomer ratio in end-stage double mutant compared with α-syn–only mice (Fig S13B and C). Note that the difference between α-syn and α-syn + clogger mice is less apparent on the gel because of relatively higher levels of the monomers in the α-syn single mutant mice. To quantitatively assess the size and number of α-syn aggregates, we performed immunofluorescence on the lumbar spinal cord. At 7 mo of age, neither α-syn nor double mutant mice showed significant pathology when probing for P-α-syn (Fig 5A). In end-stage mice, we found many puncta staining positive for P-α-syn (Fig 5A). We found that the average size of P-α-syn puncta was increased in the ventral horns of double mutant mice compared with α-syn only (Fig 5B, E, and F). Consistent with increased punctum size, the average perimeter of puncta was also increased in double mutants (Fig 5C). We also observed a decrease in the percentage of area occupied by P-α-syn in the double mutants (Fig 5D), suggesting altered distribution of P-α-syn in the ventral motor horn of the double versus single mutant mice. These data support an overall increased propensity of α-syn aggregation in the cell body of double mutant relative to α-syn single mutant mice.

### Ant1^P.A114P,A123D^ expression increases the aggregation of mitochondrial proteins in the α-syn background

**Figure S14.**
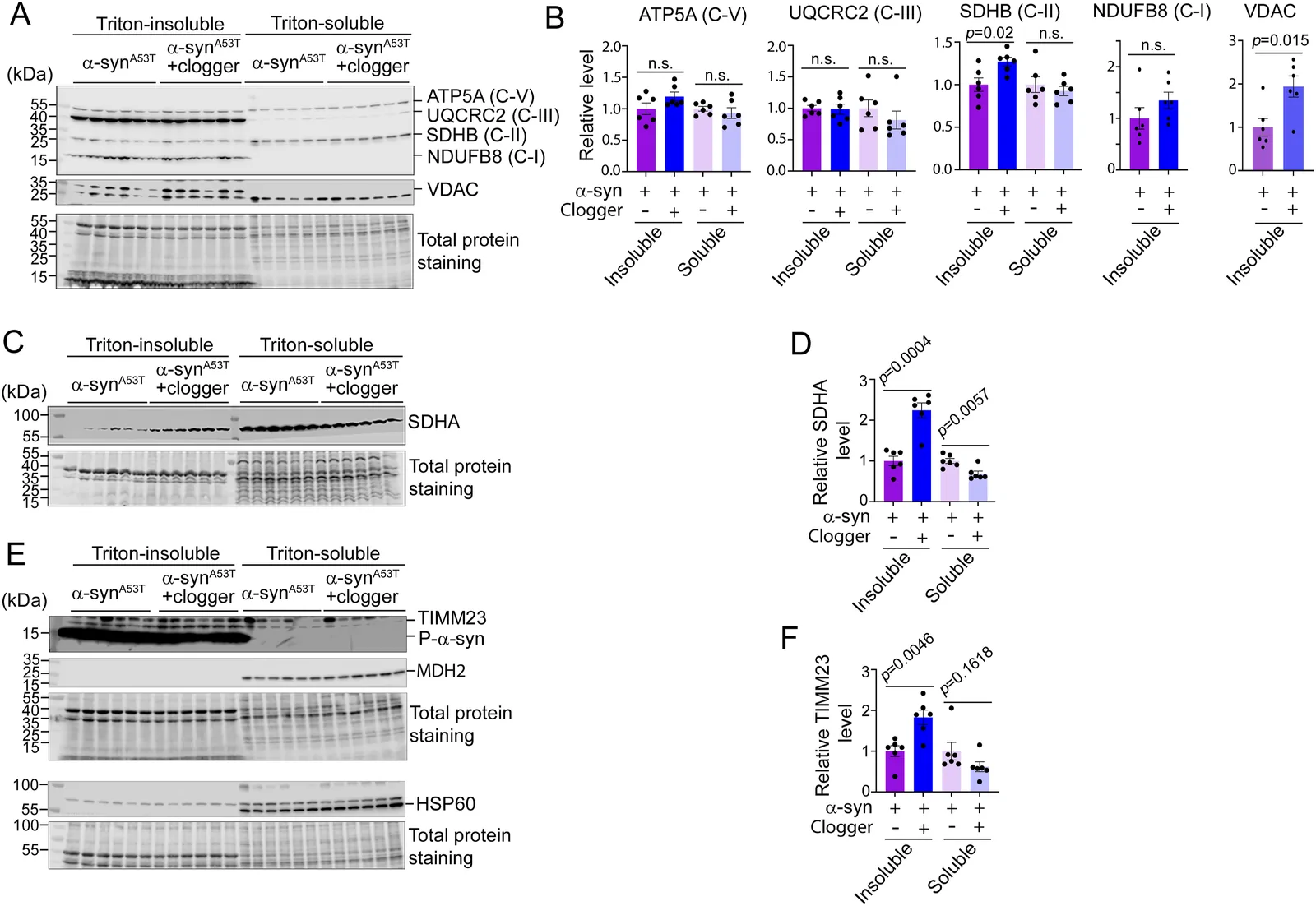
Distribution of a selected panel of mitochondrial proteins in Triton-soluble and Triton-insoluble fractions of the spinal cord from end-stage α-syn and α-syn + clogger mice. Related to Fig 6. **(A)** Western blotting analysis using a cocktail of antibodies against the indicated mitochondrial proteins: C-I, C-II, C-III, and C-V, respiratory complexes I, II, III, and V, respectively, and an antibody against VDAC. **(B)** Relative levels of proteins in soluble and insoluble fractions quantified from (A). **(C)** Western blotting analysis using an antibody against SDHA. **(D)** Quantification of (C). **(E)** Western blotting analysis using antibodies against TIMM23, HSP60, MDH2, and P-α-syn. **(F)** Quantification of (E). *P*-values were derived from a *t* test. n.s., not significant. Each lane/data point represents an individual mouse. Source data are available for this figure.

If cytosolic proteostatic stress from unimported mitochondrial preproteins is increasing α-syn aggregation, then we would expect a reciprocal increase of aggregation of mitochondrial proteins in the presence of α-syn. We used detergent solubility studies to test this hypothesis in end-stage mouse spinal cords, reasoning that the pool of detergent-insoluble mitochondrial proteins should increase in double mutants compared with α-syn alone. Indeed, we found that mitochondrial proteins such as SDHA, SDHB, VDAC, and TIMM23 are significantly enriched in the Triton-insoluble spinal cord fractions from α-syn + clogger relative to the α-syn mice (Fig S14A–F). The presence of proteins such as SDHA in the soluble fraction is accordingly reduced in the α-syn plus clogger relative to the α-syn mice. For some mitochondrial proteins (e.g., ATP5A and NDUFB8), there was a mild increase in their levels in the insoluble fractions that was not statistically insignificant (Fig S14A and B). On the other hand, several mitochondrial matrix proteins (MDH2 and HSP60) are undetectable in the Triton-insoluble fractions in α-syn and α-syn plus clogger mice (Fig S14E). Based on these observations, we conclude that aggregation of unimported mitochondrial proteins is likely dependent on their individual biochemical properties with membrane proteins more susceptible to aggregation.

**Figure 6.**
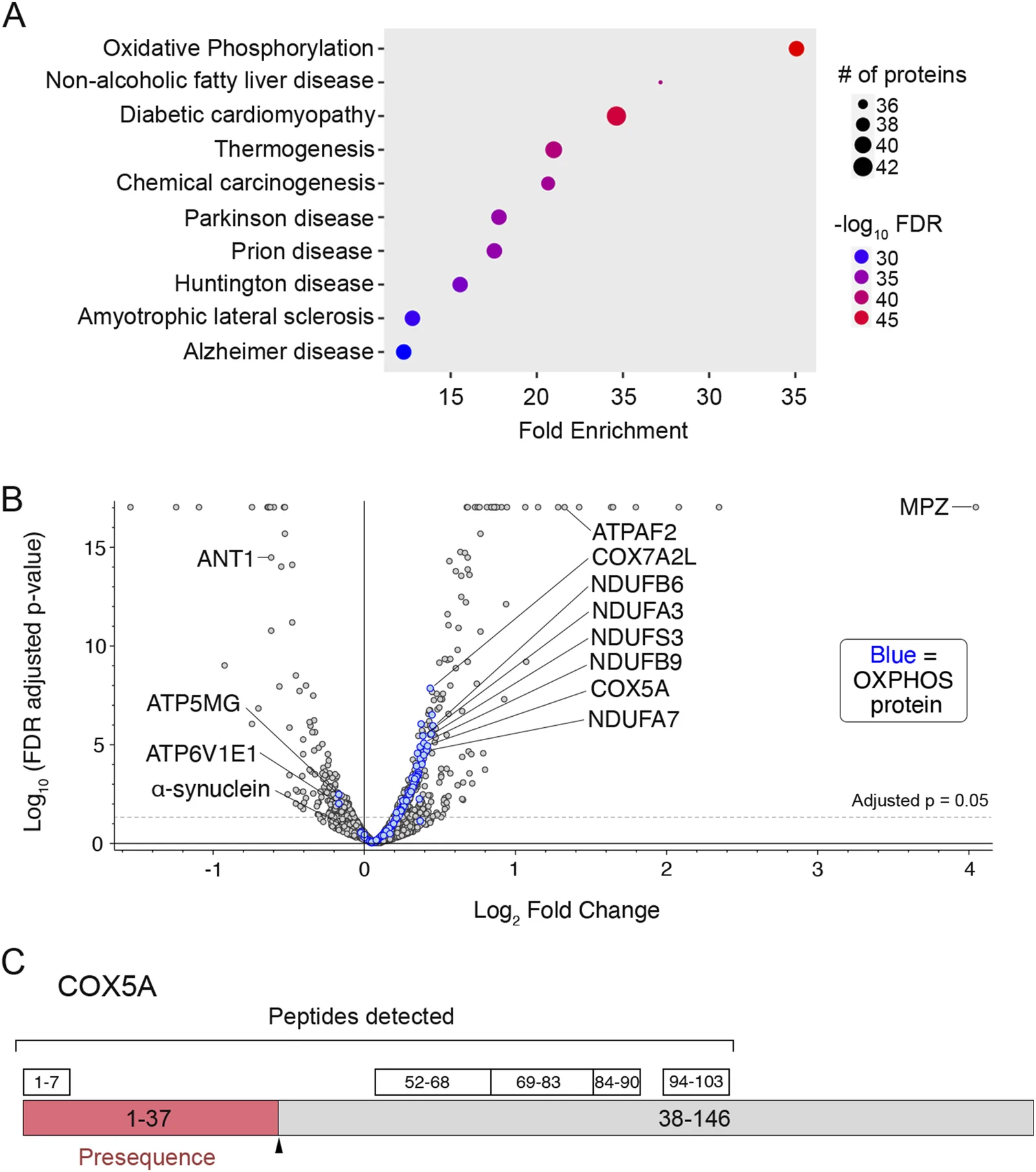
Mild protein import clogging increases aggregation of mitochondrial proteins in the α-syn background in vivo. **(A)** KEGG pathway analysis of the proteins significantly increased (FDR-adjusted *P* < 0.05) in the insoluble fraction of double mutant end-stage mouse spinal cord compared with α-syn alone (9–13.4 mo of age; n = 5 biological replicates per genotype), as determined by TMT-based quantitative proteomics. FDR, false discovery rate. **(B)** Volcano plot of TMT proteomics experiment of Triton-insoluble proteins with OXPHOS proteins labeled as blue dots (9–13.4 mo of age, n = 5 biological replicates per genotype). **(C)** Schematic of the COX5A protein and the five peptides quantified in the TMT proteomics experiment, shown above the protein (38% coverage). In red is the COX5A presequence (residues 1–37) that is normally cleaved and degraded if the protein is efficiently targeted into mitochondria.

To gain a global, unbiased view of protein aggregation, we performed tandem mass tag (TMT) quantitative proteomics on the detergent-insoluble fractions from end-stage double mutant versus α-syn mouse spinal cords (9–13.3 mo of age, n = 5 biological replicates per genotype). We found 185 proteins significantly increased in the double mutants (FDR-adjusted *P* < 0.05, Table S6). Of these, 55 were ascribed to the “mitochondrion” Cell Component, which was significantly overrepresented (GO:0005739, FDR < 10^−15^). KEGG pathway analysis showed “oxidative phosphorylation” as the most enriched group of proteins that are increased in the insoluble fractions from double mutant spinal cords (Fig 6A and B, FDR < 10^−42^). This was not driven by increased total α-syn levels (Fig 6B). Interestingly, multiple neurodegenerative disease KEGG pathways were also enriched, most notably PD (Fig 6A). Finally, we detected a unique peptide within mitochondrial targeting presequence of COX5A, a protein significantly increased in the double mutant aggregates (Fig 6C). This peptide is expected to be cleaved and degraded if COX5A efficiently enters mitochondria. Our observation suggests the presence of unimported mitochondrial preproteins within the protein aggregates, although it remains unclear whether they physically co-aggregated with α-syn.

Table S6. TMT mass spectrometry analysis of the spinal cord Triton-insoluble proteome from *Ant1*^*P.A114P,A123D*^/+ α-syn double mutant compared with α-syn single mutant mice.

## Discussion

Two important hallmarks shared among prevalent neurodegenerative diseases such as PD, Lewy body dementia, Alzheimer’s disease, and ALS are mitochondrial dysfunction and cytosolic protein misfolding/aggregation. Whether and how these hallmarks interact in disease pathogenesis is an open question. Studies in yeast and cultured human cells over the last decade have shown that a diverse range of mitochondrial insults can affect protein import and cause the toxic accumulation and aggregation of mitochondrial preproteins in the cytosol, which causes mPOS (Wang & Chen, 2015; Wrobel et al, 2015; Coyne & Chen, 2026). Previous studies have shown that mitochondrial protein import stress and mPOS accelerate the cytosolic aggregation of neuropathogenic proteins such as huntingtin, α-syn, and Aβ in yeast and *Caenorhabditis elegans* (Wang & Chen, 2015; Nowicka et al, 2021). It is yet to be tested whether mPOS contributes to or modify the course of disease pathology in mammals by affecting cytosolic proteostasis independent of bioenergetic defects of mitochondria. The critical barrier to addressing this question has been modeling protein import defects in vivo without confounding oxidative phosphorylation deficiency and/or lethal shutdown of mitochondrial biogenesis. In this study, we overcome this roadblock by establishing a mouse model of mitochondrial protein import stress that expresses Ant1^P.A114P,A123D^. These mice lack detectable bioenergetic defects but exhibit low-grade proteostatic stress and inflammatory response in the CNS. To assess the role of mild mPOS in neurodegenerative disease, we expressed Ant1^P.A114P,A123D^ in an established mouse model of neurodegeneration caused by expression of human α-syn with a pathogenic A53T mutation. The mutant α-syn is a highly aggregation-prone protein that causes an autosomal dominant cause of familial PD (Lee et al, 2002b). We tested whether Ant1^P.A114P,A123D^ expression alters the proteostatic dynamics and pathogenicity of α-syn independent of mitochondrial bioenergetics.

The data presented provide evidence that Ant1^P.A114P,A123D^ expression is sufficient to accentuate motor coordination defects and α-syn aggregation independent of bioenergetics. Ant1^P.A114P,A123D^ expression likely induces protein import stress to affect α-syn aggregation by challenging the global proteostatic network in the cytosol, which secondarily stimulates α-syn misfolding and aggregation/oligomerization. These biochemical changes correlate with increased neuroinflammation. Importantly, bioenergetic defects cannot explain the phenotype augmentation by Ant1^P.A114P,A123D^ in α-syn mice. We did not observe a reduction in mitochondrial respiration, COX activity, or the levels of OXPHOS components in the CNS tissues of double mutant mice. This may seem contrary to the observation that OXPHOS proteins appear to be aggregation-prone in the double mutants. It is known that mild depletion of specific proteins/complexes does not always cause significant deficiency in the overall OXPHOS capacity (Brand & Nicholls, 2011), likely because of high reserve capacities of individual respiratory components. An additional consideration is whether altered Ant1 function underlies phenotypes in double mutant mice, given that A123D mutation in Ant1^P.A114P,A123D^ renders the protein catalytically inactive (Palmieri et al, 2005). However, Ant1^P.A114P,A123D^ and its yeast counterpart exert gain-of-function toxicity in cells and there is no evidence for limited ADP/ATP transport in the respiration experiments in the CNS tissues. Taken together, although we cannot completely exclude a subtle effect from undetectable bioenergetic deficiency, the data suggest that Ant1^P.A114P,A123D^ expression can accentuate neural damage in α-syn A53T mice independent of bioenergetics, suggesting that mild mitochondrial protein import stress and consequent mPOS may be pathogenic in these models. Our findings therefore add a new layer to the complex relationship between mitochondrial dysfunction and neuropathogenic protein aggregates, which warrants testing in further PD mouse models and other models of neurodegeneration such as Alzheimer’s disease and ALS in which mitochondrial abnormalities have been extensively reported. It is important to note that we determined mitochondrial respiration at the asymptomatic stage of the α-syn A53T mice. This is critical to avoid the onset of excessive lesions to the neural tissues, which may secondarily affect mitochondrial function. Our data do not support bioenergetic defects as an early driver of α-syn–induced pathogenesis.

The largely nonsymptomatic Ant1^P.A114P,A123D^/+ mice provided a critical experimental tool for determining the bioenergetic-independent effect of mitochondrial protein import stress on the CNS. Several lines of evidence support Ant1^P.A114P,A123D^-induced neural damage. Although the frequency is low, a fraction of the clogger mice do develop a neurodegenerative phenotype (Coyne et al, 2023). Furthermore, bulk RNA-seq combined with deconvolution analysis provided a sensitive method to reveal proteostatic responses and the microglial and astrocyte activation in CNS tissues of aged Ant1^P.A114P,A123D^/+ mice. One caveat of the model and limitation of this study is that the mutant Ant1 is present at extremely low levels in the CNS to varying degrees depending on cell type (Coyne et al, 2023). This precluded the use of orthogonal techniques such as co-immunoprecipitation to document its increased retention to the protein import channels, and the analysis of the cytosolic proteome for direct detection of mitochondrial precursor accumulation in the cytosol. Therefore, direct evidence of protein import clogging in the CNS of clogger mice is lacking. However, the clogging activity of the mutant *Ant1* has been biochemically demonstrated in yeast, cultured human cells, and mouse skeletal muscle (Coyne et al, 2023). Mitochondrial protein import clogging would be reasonably expected to occur in the CNS tissues as well.

We chose to test mPOS in a model of PD because it has long been appreciated that mitochondrial dysfunction can cause cytosolic α-syn aggregation, but the molecular mechanisms have remained unknown. Moreover, the roles of bioenergetic deficiency and/or nonbioenergetic mechanisms are unclear. In line with a role for bioenergetic deficiency, toxins that inhibit mitochondrial complex I, such as MPTP and rotenone, cause α-syn aggregation and PD in humans, human cells, and animal models (Davis et al, 1979; Burns et al, 1983; Langston et al, 1983; Betarbet et al, 2000; Lee et al, 2002a; Chaves et al, 2010). Mutations in mtDNA in dopaminergic neurons, which would certainly cause bioenergetic deficiency, can induce the formation of cytosolic inclusions that contain mitochondrial proteins (Ekstrand et al, 2007; Sterky et al, 2011). mtDNA mutations have also been shown to cause degeneration of dopaminergic neurons and motor deficit when synergized with the loss of parkin, an E3 ubiquitin ligase mutated in an early onset form of PD (Pickrell et al, 2015). These findings implicate mitochondrial bioenergetics in pathogenic α-syn aggregation. It is important to note that in addition to impaired ATP synthesis, damage to the OXPHOS pathway itself may lead to reduced protein import and mPOS by affecting mitochondrial processes such as membrane potential maintenance and protein translocase assembly. Inhibition of complex I with MPTP has been shown to reduce mitochondrial protein import in cells and in vitro (Franco-Iborra et al, 2018). Reduced protein import aggravates seeding of α-syn aggregates in cell models (Lautenschlager et al, 2020). Moreover, mitochondrial proteins are found in α-syn aggregates in PD patient brains (Hashimoto et al, 1999; Gandhi et al, 2006; Leverenz et al, 2007; Power et al, 2017). Overall, in addition to bioenergetic deficiency, we show proof-of-concept that mPOS can be an additional, nonbioenergetic pathogenic consequence of mitochondrial dysfunction in neural damage. This novel neuropathological mechanism could be particularly relevant under disease conditions with mild mitochondrial damage and moderate or no bioenergetic deficiency.

Our finding that Ant1^P.A114P,A123D^ expression is sufficient to alter the course of α-syn–induced neural damage could have implications for better understanding of PD. As mentioned above, mitochondrial stress has been widely reported in PD patient samples. It is expected that genetic and environmental PD inducers (e.g., defective mitochondrial protein quality control and exposure to MPTP or rotenone) would reduce proton pumping across the IMM, which results in reduced membrane potential and protein import (Coyne & Chen, 2018). On top of this, α-syn itself may impair mitochondrial protein import by directly binding to the TOM20 import receptor (Di Maio et al, 2016), and/or by indirectly decreasing mitochondrial protein import through an effect on other mitochondrial processes such as membrane potential maintenance (Reeve et al, 2015), electron transport chain integrity, mitochondrial dynamics/transport (Sohrabi et al, 2023), and protein quality control (Zhang et al, 2024). As expression and accumulation of α-syn increase in PD-affected neurons with normal aging (Chu & Kordower, 2007), it is possible that an age-dependent increase in the α-syn level gradually reduces mitochondrial protein import, thereby causing mPOS to aggravate its own aggregation in the cytosol. A similar process may happen in certain forms of familial PD. Notably, loss of PINK1, which is a recessive cause of PD, has also been convincingly shown to reduce mitochondrial protein import (Gispert et al, 2009).

We found that Ant1^P.A114P,A123D^ expression increases the size of P-α-syn–positive puncta in the end-stage α-syn + clogger double mutant compared with α-syn–only mice. We also found that Ant1^P.A114P,A123D^ expression drives a pool of mitochondrial preproteins to become detergent-insoluble in symptomatic α-syn mice. These observations support the effect of mPOS in the cytosol on the distribution of P-α-syn. However, additional studies are required to determine how this affects the misfolding and aggregation dynamics of α-syn. It is also important to note that the increased size of P-α-syn–positive puncta was observed at the end stage of the mutant mice. It remains unclear whether Ant1^P.A114P,A123D^ expression accelerates protein aggregation during disease progression or instead exacerbates pathology after onset of neurodegeneration. Indeed, large aggregates of α-syn do not necessarily correlate with high toxicities (Lashuel et al, 2013).

In summary, we used our unique clogger mouse model to assess the neurological effects specific to mitochondrial protein import stress and subsequent mPOS. We found that Ant1^P.A114P,A123D^ expression can be mitigated to some extent in healthy mice but alters protein aggregation, neuroinflammation, and motor deficits in a mouse model of PD without a detectable effect on mitochondrial bioenergetics. Thus, mitochondrial protein import efficiency and mPOS may be disease-modifying factors in neurodegeneration independent of OXPHOS.

## Materials and Methods

### Experimental models

*Slc25a4* ^p.A114P,A123D^/+ mice, or “clogger” mice, were generated as previously described (Coyne et al, 2023). After >10 backcrosses with the C57BL/6NTac females (catalog no.: B6-F; Taconic), male *Slc25a4* ^p.A114P,A123D^/+ mice were crossed with female C57BL/6 mice with transgenic expression of the human *SNCA* gene (coding for the α-syn protein) harboring the pathogenic A53T mutation (#006823; Jackson Lab) (Lee et al, 2002b). *SNCA* expression is driven by the mouse prion promoter in these mice. After the first cross between clogger and *SNCA* transgenic mice, double mutant mice were backcrossed against female C57BL/6NTac mice. Where α-syn mice were tested, all WT and clogger control mice were littermates.

All procedures were approved by the Animal Care and Use Committee at State University of New York Upstate Medical University and were in accordance with guidelines established by the National Institutes of Health. Animals in the α-syn background were examined daily for signs of illness for determination of “end stage” for lifespan, histological, and biochemical analyses. An animal was considered “end stage” once it displayed paralysis and was so severely moribund that it was determined unlikely to survive more than an additional 48 h, as judged by an experienced technician in our Department of Laboratory Animal Resources. A mouse was considered severely moribund if it also exhibited the following clinical signs: inability to eat, drink, or eliminate; severe dehydration; labored breathing; severe lethargy. Other parameters used that were used in lifespan determination, but not specific to “end-stage” α-syn mice, were edema, sizable abdominal enlargement or ascites, significant skin lesions exposing muscle, progressive dermatitis, or a severely ulcerated or bleeding tumor.

### Mitochondrial isolation and purification

For mitochondrial respiratory studies, mice were euthanized by decapitation without CO_2_ asphyxiation or anesthetic. Neural tissues were rapidly dissected (<60 s) and placed in 1 ml of ice-cold isolation medium (IM) (225 mM mannitol, 75 mM sucrose, 5 mM Hepes-KOH, pH 7.4, 1 mM EGTA, 0.1% BSA). Quick dissection of the spinal cord was enabled via hydraulic extrusion with ice-cold PBS (Richner et al, 2017). Tissue was minced on ice while submerged in IM as soon as possible. For crude mitochondrial fraction isolation (i.e., for cerebellum, spinal cord, and brain stem), well-minced tissue was homogenized with four strokes by hand in a 2-ml glass Dounce homogenizer (pestle B, clearance 0.0005–0.0025 inches), followed by differential centrifugation at 4°C. First, the homogenate was centrifuged at 2,000*g* for 5 min. The supernatant was then centrifuged at ∼21,000*g* for 20 min. The pellet was resuspended in 0.5 ml IM and centrifuged again at ∼21,000*g* for 20 min. Again, the pellet was resuspended in 0.5 ml IM and centrifuged again at ∼21,000*g* for 20 min. The final pellet was resuspended in 0.2 ml IM.

Mitochondria were purified from the forebrain, which in our studies includes all neural tissue anterior to the junction between the cerebral cortex and the cerebellum. This was done using discontinuous Percoll gradient centrifugation, essentially as described (Kristian, 2010). Minced forebrains were homogenized with three strokes with the pestle at 13,000*g* in ice-cold 7 ml IM. The homogenate was then centrifuged for 5 min at 2,000*g*. The resulting supernatant was centrifuged at 13,000*g* for 12 min. The pellet was then resuspended in 2 ml IM containing 12% Percoll and used to build a discontinuous gradient using the following Percoll concentrations in isotonic IM: 5 ml 40%, 5 ml 23%, and ∼2.5 ml 12% (mito). Gradients were ultracentrifuged in a swinging-bucket rotor (SW 41) at ∼43,000*g* for 20 min with slow acceleration and deceleration. Purified nonsynaptosomal mitochondria were retrieved from the 23/40% interface, and synaptosomes were collected from the 23/12% interface. Both fractions were then diluted with ∼25 ml IM without Percoll, and centrifuged at 13,000*g* for 15 min. The mitochondrial pellet was resuspended in 1 ml IM and centrifuged at 13,000*g* for 12 min. Mitochondria were then washed two more times in 1 ml ice-cold IM and finally resuspended in 80 μl for nonsynaptosomal pure mitochondria, and 200 μl for synaptosomes. Protein concentration was then determined by the Bradford assay.

### Mitochondrial oxygen consumption measurements

Oxygen consumption assays were performed using the Oxygraph system from Hansatech, which has a small water-jacketed (37°C), magnetically stirred chamber sitting atop a Clark electrode. Each reaction occurred in 0.5 ml respiratory buffer (125 mM KCl, 4 mM K_2_HPO_4_, 3 mM MgCl_2_, 1 mM EGTA, 20 mM Hepes-KOH, pH 7.2). 150 μg or 300 μg of nonsynaptosomal and synaptosomal mitochondria, respectively, were added first. Then, for complex I–based respiration, reagents were added in the following sequence at the indicated final concentrations: glutamate (5 mM) + malate (2.5 mM); ADP (200 μM); oligomycin (3 μg/ml); FCCP (0.2 μM); and for complex II: rotenone (5 μM); succinate (10 mM); ADP (300 μM); oligomycin (3 μg/ml); FCCP (0.2 μM).

### Respiratory complex activity assay

The COX activity assay was performed essentially as described previously (Spinazzi et al, 2012). Oxidized cytochrome *c* (C2506; Sigma-Aldrich) was reduced with sodium dithionate and dialyzed overnight in potassium phosphate buffer, followed by spectrophotometric confirmation that cytochrome *c* was reduced (OD 550/560 ratio > 6). For the activity assay, snap-frozen crude spinal cord mitochondria were thawed on ice and 1-ml reaction tube was prepared with 20 mM potassium phosphate, pH 7.0, 0.45 mM DDM, and 15 μM reduced cytochrome *c*. At time zero, 0.02 mg mitochondria (spinal cord) or 0.2 mg mitochondria (brainstem, forebrain, cerebellum) were added to the reaction and OD550 was monitored every 2 s for 5 min. The slope over the first 12 s of the assay was used to determine the rate constants.

### Detergent solubility studies

Triton lysis buffer (0.5% Triton X-100, 150 mM NaCl, 50 mM Hepes-KOH, pH 7.4, 1 mM EDTA with protease and phosphatase inhibitors [Roche]) was added to dissected and snap-frozen tissues at a ratio of 6 μl/mg of tissues. Tissue was minced and homogenized in a 2-ml Dounce homogenizer, then incubated on ice for 30 min. Triton-insoluble fractions were collected by centrifugation at 21,000*g* for 20 min, then washed twice by vortexing in Triton lysis buffer. The soluble supernatant was centrifuged an additional two times at 21,000*g* for 30 min each. Triton-insoluble fractions were solubilized with 5% SDS and 8 M urea, mixed with pipetting, and sonicated for ∼10 s. Samples were then incubated at 42^o^C for 30 min before centrifugation at 16,000*g* for 20 min at room temperature. The supernatant was the final “Triton-insoluble fraction” and was either directly loaded onto an SDS–PAGE gel for Western blot analysis or processed for quantitative mass spectrometry as described below.

### Sample processing for quantitative mass spectrometry

Five biological replicates of α-syn and double mutant samples were prepared for multiplexed quantitative mass spectrometry as above. Samples were buffer-exchanged on a 3-kD molecular weight cutoff filter (Amicon 3k Ultracel) using four additions of 10 mM triethylammonium bicarbonate, pH 8.0 (Thermo Fisher Scientific). 100 μg was taken for digestion using an EasyPep Mini MS sample prep kit (A40006; Thermo Fisher Scientific). To each buffer-exchanged sample, 65 μl of lysis buffer was added followed by 50 μl of reduction solution and 50 μl of alkylating solution. Samples were incubated at 95°C for 10 min, then cooled to room temperature. To each sample 5 μg of trypsin/Lys-C protease was added, and the reaction was incubated at 37°C overnight. Half of each digest was used for subsequent labeling. TMT reagents were reconstituted with 40 μl acetonitrile (ACN) and the contents of each label added to a digested sample. After 60 min, 50 μl of quenching solution was added, consisting of 20% formic acid and 5% hydroxylamine (vol/vol) in water. The labeled digests were cleaned up by a solid-phase extraction device contained in the EasyPep kit and dried by speed-vac. The individually labeled samples were dissolved in 50 μl of 30% ACN and 0.2% trifluoroacetic acid (vol/vol) in water, and 15 μl of each was used to create a pooled sample consisting of 150 μg.

### Fractionation

After an LC-MS experiment to check digestion and labeling quality of the pooled samples, 100 μg of the pooled sample was fractionated using Pierce High pH Reversed-Phase Peptide Fractionation Kit (part # 84868), per the manufacturer’s instructions for TMT-labeled peptides. In brief, samples were dissolved in 300 μl of 0.1% trifluoroacetic acid in water and applied to the conditioned resin. Samples were washed first with water and then with 300 μl of 5% ACN, 0.1% triethylamine in water. The second wash was collected for analysis. Peptides were step-eluted from the resin using 300 μl of solvent consisting of 5–50% ACN with 0.1% triethylamine in eight steps. All collected fractions were dried in a speed-vac.

### LC-MS/MS

Dried fractions were reconstituted in 25 μl of load solvent consisting of 3% ACN and 0.5% formic acid in water, and a 5 μl aliquot was diluted 1:3 with the same solvent. Of the 15 μl, 2 μl was injected onto a pulled tip nano-LC column (FS360-75-10-N; New Objective) with 100 μm inner diameter packed to 32 cm with 1.9 μm, 100 Å, C18AQ particles (Magic 2, PremierLCMS). The column was maintained at 50°C with a column oven (Sonation GmbH, PRSO-V2). The peptides were separated using a 135-min gradient consisting of 3–12.5% ACN over 60 min, 12.5–28% over 60 min, 28–85% ACN over 7 min, a 3-min hold, and 5-min re-equilibration at 3% ACN. The column was connected in-line with an Orbitrap Lumos (Thermo Fisher Scientific) via a nanoelectrospray source operating at 2.3 kV. The mass spectrometer was operated in data-dependent top speed mode with a cycle time of 3 s. MS^1^ scans were collected from 375 to 1,500 m/z at 120,000 resolution and a maximum injection time of 50 ms. HCD fragmentation at 40% collision energy was used followed by MS^2^ scans in the Orbitrap at 50,000 resolution with a 105-ms maximum injection time.

### Database searching and reporter ion-based quantification

The MS data were searched using SequestHT in Proteome Discoverer (version 2.4, Thermo Fisher Scientific) against the *Mus musculus* proteome from UniProt, containing 50,961 sequences and a list of common laboratory contaminant proteins. Enzyme specificity was set for fully tryptic with up to two missed cleavages. Precursor and product ion mass tolerances were 10 ppm and 0.02 Da, respectively. Cysteine carbamidomethylation, TMT 10-plex at any N terminus, and TMT 10-plex at lysine were set as fixed modifications. Methionine oxidation was set as a variable modification. The output was filtered using the Percolator algorithm with strict FDR set to 0.01. Quantification parameters included the allowance of unique and razor peptides, reporter abundance based on intensity, lot-specific isotopic purity correction factors, normalization based on total peptide amount, protein ratio based on protein abundance, and background-based hypothesis testing (*t* test). The final protein list was analyzed for enrichment using STRING database version 11.5 (Szklarczyk et al, 2023) and ShinyGO 0.77 (Ge et al, 2020).

### Immunoblot analysis

Purified brain mitochondria were solubilized in Laemmli buffer and processed for Western blotting using standard procedures and Total OXPHOS Antibody Cocktail (Abcam; #ab110411). Briefly, the solubilized protein was run on a 4–20% SDS–PAGE gel (Bio-Rad). After electrophoretic transfer to a PVDF membrane, Revert Total Protein Stain (LI-COR Biosciences) was applied to membrane per the manufacturer’s instructions. Membranes were blocked in Tris-buffered saline + 0.1% Tween-20 containing 5% milk. For the primary antibodies, the mouse monoclonal MitoProfile Total OXPHOS Human WB antibody cocktail was diluted at 1:500 (#ab110411; Abcam). Other antibodies were diluted as recommended by manufacturers. Blots were visualized with Pierce ECL Western Blotting Substrate.

### Mouse perfusion and CNS cryosectioning

Mice were perfused via the heart as described previously (PMID: 35129175). Briefly, animals were anesthetized with inhaled isoflurane before sequential cardiac perfusion with PBS (pH 7.4) followed by fresh 4% PFA dissolved in PBS (pH 7.4). The brain was removed and divided into right and left hemispheres via the longitudinal fissure. The spine was removed and divided into cervical and lumbar regions using the cervical and lumbar enlargements as landmarks. All tissues were cryoprotected in increasing concentrations of sucrose and embedded in Tissue-Tek OCT compound (#4583; Sakura Finetek). Tissue sections were obtained using a Leica CM1950 cryostat. Lumbar cryosections were obtained at 30 mm and stored in a cryoprotectant solution (20% glycerol, 30% ethylene glycol, 50% PBS, pH 7.4) at −20°C. Individual brain hemispheres were sectioned coronally beginning at the olfactory bulb. 30-mm striatal sections were collected into cryoprotectant and stored at −20°C. 14-mm sections at the level of the substantia nigra pars compacta were collected onto Superfrost Plus slides (#12-550-15; Thermo Fisher Scientific) in an alternating series of four slides, air-dried, and stored airtight at −70°C.

### Nissl staining and quantification

For Nissl staining, lumbar spines were thawed, washed in PBS, and mounted onto Superfrost Plus slides and air-dried. Slides were “defatted” overnight in a 1:1 mixture of absolute ethanol and chloroform. The next day, slides were hydrated through sequential immersions in absolute ethanol, 95% ethanol, and distilled water. A Coplin jar containing cresyl violet solution (#C5042; Sigma-Aldrich) was warmed to 42°C, and slides were incubated in this solution for 5 min. Slides were rinsed with distilled water before being dehydrated and cleared through sequential immersions in 95% ethanol, absolute ethanol, and xylene. Slides were mounted with Leica Micromount xylene-based mounting medium (#3801731; Leica Biosystems) and dried overnight. Whole slide scans were obtained at 20× using the Aperio AT2 slide scanner (Leica Biosystems). For image analysis, raw (“.svs”) files from the slide scanner were imported into QuPath (PMID 29203879). Motor neurons were counted in the anterior horn of the spinal cords in a blinded manner using criteria modified from Lee et al (PMID:16399671). Motor neurons were identified based on a large size of the soma (diameter greater than ∼20 mm), a pale staining round nucleus, and globular Nissl stain within the cytosol. Atrophic (chromatolytic) neurons were defined by a large size of the soma (diameter greater than ∼20 mm), hypercondensation of nuclear material, and a pale-pink homogeneous cytosol.

### Immunofluorescence of slide-mounted sections

For anti-α-syn phospho-S129 probing in the lumbar spine, sections were thawed, washed in PBS, mounted onto Superfrost Plus slides, and air-dried. Dried slides were washed in pH 7.4 PBS + 0.05% Tween-20 (PBST) and incubated for 1 h in blocking buffer (10% normal goat serum + 0.3% Triton X-100 dissolved in PBST) at room temperature. Blocking buffer was discarded, and slides were washed and incubated with primary antibody: rabbit anti-α-syn (phospho-S129) (#ab51253; Abcam, 1:10,000) diluted in blocking buffer overnight. The primary antibody was discarded, and slides were washed and incubated with secondary antibody: Alexa Fluor 633–conjugated goat anti-rabbit IgG (H+L) (#A-21070; Invitrogen, 1:500) diluted in blocking buffer for 2 h. The secondary antibody was discarded, and slides were washed and mounted with ProLong Gold Antifade Mountant with DAPI (#P36391; Invitrogen).

### Immunofluorescence of free-floating sections

Free-floating sections from the lumbar spine and striatum were thawed and washed in PBST before incubation for 1 h in blocking buffer (10% normal goat serum + 0.3% Triton X-100 dissolved in PBST) at room temperature. Blocking buffer was discarded, and sections were washed and incubated with primary antibody overnight. Lumbar sections were incubated in rabbit anti–vesicular acetylcholine transporter (#PA5-85782; Invitrogen, 1:1,000), mouse anti-GFAP (#3670; Cell Signaling Technology, 1:2,000), or rabbit anti-ubiquitin (#701339; Invitrogen, 1:4,000). Striatal sections were incubated with chicken anti-tyrosine hydroxylase (#PA5-143583; Invitrogen, 1:400) or rabbit anti-ubiquitin (#701339; Invitrogen, 1:1,000). Primary antibody was discarded, and sections were washed and incubated with the appropriate secondary antibody: Alexa Fluor 633–conjugated goat anti-rabbit IgG (H+L) (#A-21070; Invitrogen, 1:500), DyLight 550–conjugated goat anti-mouse IgG (H+L) (#84540; Invitrogen, 1:500), or Alexa Fluor 488–conjugated goat anti-chicken IgY (H+L) (#A11039; Invitrogen, 1:500) diluted in blocking buffer for 2 h. The secondary antibody was discarded, and sections were washed before collection onto Superfrost Plus slides and mounting with ProLong Gold Antifade Mountant with DAPI (#P36391; Invitrogen).

### CNS dissection

Neural tissues were dissected and snap-frozen as quickly as possible, and the spinal cord was ejected from the spinal canal via hydraulic extrusion with ice-cold DEPC-treated PBS. Accurate identification of the striatum was less straightforward than the cerebellum and spinal cord. First, cerebellum, brainstem, and olfactory bulb were removed from the forebrain. The brain was then cut sagittally along the interhemispheric fissure. Approaching from the medial aspect of the brain, the inner brain material (including the thalamus, septum, and underlying striatum) was removed by cutting just below the corpus callosum, thus separating these tissues from the hippocampus and associated cerebral cortex. The easily identifiable thalamus and hypothalamus were removed, and the remaining tissue from this “inner brain” material was labeled striatum. Tissue was frozen in liquid nitrogen.

### RNA purification and sequencing

Tissue was disputed in QIAzol lysis reagent, using the Qiagen TissueRuptor. RNA was then extracted using Qiagen miRNeasy Mini Kit. RNA quality and quantity were assessed with the RNA 6000 Nano kit on the Agilent 2100 Bioanalyzer. For striatum, sequencing libraries were prepared using the Illumina TruSeq Stranded mRNA Library Prep kit, using 1 μg total RNA as input. For cerebellum and spinal cord, sequencing libraries were prepared using Illumina Stranded mRNA Library Prep kit Ligation, using 500 ng total RNA as input. The two library prep kits are equivalent. Library size was assessed with DNA 1000 Kit on the Agilent 2100 Bioanalyzer. Libraries were quantified using Quant-IT High Sensitivity dsDNA Assay (Invitrogen) on Qubit 3.0 Fluorometer. Libraries were sequenced on the NextSeq 500 instrument, with paired-end 2x75 bp reads.

### RNA-seq analysis

RNA-seq analysis was performed from the spinal cord at 30 mo of age with five biological replicates per genotype, the cerebellum at 30 mo of age with five biological replicates per genotype, and the striatum at 24 mo of age with four biological replicates per genotype. Initial analysis was performed on Partek Flow Genomic Analysis Software. All reads were aligned to the *M. musculus* genome (mm10) using STAR-2.7.3a and quantified using Partek E/M. Reads were normalized with transcripts per million and quantile normalization including all samples from the spinal cord, cerebellum, and striatum. We probed for differentially expressed genes using ANOVA, analyzing data from all tissues together. Within-tissue analyses shown as heatmaps, volcano plots, and gene lists were generated in Partek from this analysis. Similarly, pathway analysis shown for the striatum data was performed using Partek Pathway Analysis software. For volcano plots, the *Slc25a4* transcript was omitted to facilitate visualization.

RNA-seq deconvolution was performed in a Jupyter Notebook using Python (v3.10+) with Pandas (v2.0+) and NumPy for data structures and matrix operations, the SciPy optimize.nnls module for deconvolution and cell-type inference, and Seaborn and Matplotlib for generation of categorical plots, heatmaps, and trend lines. Bulk RNA-seq data (read counts/transcripts per million) were loaded from tab-delimited text files. For visualization (heatmaps) and certain statistical distributions, data were log2-transformed using log2 (x+1). Cell type–specific marker genes were derived from a curated brain and spinal cord marker list created from the PanglaoDB database (panglaodb.se), cross-referenced to the Allen Brain Cell Atlas (celltypes.brain-map.org). The manually curated signature file focused on nine distinct cell populations: astrocytes, microglia, neural stem cells, oligodendrocyte precursor cells, oligodendrocytes, Schwann cells, pan-neuronal cells, excitatory neurons, and inhibitory neurons. Only markers present in the bulk RNA-seq dataset were used for the deconvolution signature matrix (i.e., imputation of missing marker gene expression data was not performed). Cellular proportions were estimated using the marker genes using the non-negative least squares deconvolution method in the optimize.nnls SciPy module. During this process, a binary signature matrix (A) was constructed where Ai,j = 1 if gene i is a marker for cell type j, and 0 otherwise. For each sample bulk expression vector (b), we solved for the proportion vector (x) such that |Ax−b|^2 is minimized, subject to x ≥ 0. Resulting coefficients were normalized to sum to 1 to represent relative cell-type proportions. To evaluate differences in estimated cell-type proportions between genotypes (knock-in versus WT), two-sided Welch’s *t* tests (unequal variance) were used to compare proportions within specific tissues and age groups. A *P*-value <0.05 was considered statistically significant for these exploratory analyses. Longitudinal trends across 9, 24, and 30 mo were visualized using line plots with mean and SEM indicated. Faceted whisker box plots were created for the marker genes in selected cell types.

### Gene set enrichment analysis

Functional enrichment analysis was performed using the GSEApy library (v1.3.0) to identify biological pathways that were significantly altered by comparing clogger and WT mice in the 24-mo and 30-mo spinal cord. A gene set enrichment analysis preranked analysis was used for this, with genes sorted by the difference in means of quantile-normalized log_2_-transformed expression values. Enrichment was tested against the MSigDB Hallmark 2020, GO Biological Process 2023, and KEGG 2019 Mouse gene set collections, with significance determined using 100 permutations, and pathways considered significant at a nominal *P*-value <0.05. Visualizations were generated using bubble plots scaled by leading-edge gene counts and colored by normalized enrichment scores to illustrate the functional trajectory of aging-associated transcriptional changes.

### Real-time quantitative reverse transcription PCR

Total RNA was isolated from mouse spinal cord. RNA concentration and purity were determined using a NanoDrop spectrophotometer (Thermo Fisher Scientific), and samples were diluted to a final concentration of 1 ng/μl. Gene expression was quantified by one-step RT–qPCR using Luna Universal One-Step RT–qPCR Kit (New England Biolabs) according to the manufacturer’s protocol. *Gapdh* was used as the reference gene for normalization, and relative gene expression was calculated using the comparative Ct (2^−ΔΔCt) method. The following primers were used for the amplification of the specific genes: 5′-TGA​TAG​CTG​CTT​GGG​CAC​CGA-3′ and 5′-TGC​ACA​GTG​CTG​CTC​CCA​ACA-3′ for *Hspa1b*, 5′-GTC​GCA​TGC​TTG​CCA​AAG​AG-3′ and 5′-GGT​GGT​AAC​ATC​AGG​GG-3′ for *Igf2*, 5′-GGT​CTA​CAG​CCC​TAA​GTG​CG-3′ and 5′-GCA​GGG​GCC​CAT​CTC​ACT​AT-3′ for *Igfbp6*, and 5′-ACC​CAG​AAG​ACT​GTG​GAT​GG-3′ and 5′-CAC​ATT​GGG​GGT​AGG​AAC​AC-3′ for *Gapdh*.

### Behavioral assays

Behavioral assays were performed in a particular order to minimize the likelihood that one test affects mouse behavior on subsequent days. The order went as follows: elevated plus maze (EPM), Y-maze spontaneous alternation test, open field activity test, novel object recognition test, Morris water maze, followed by rotarod testing. Male and female mice were tested in all assays. Each data point shown in behavioral assays represents an independent mouse. EPM, Y-maze spontaneous alternation, and open field activity testing were done on two independent cohorts. For every test, mice were habituated to the testing room for 30 min before each test, and odors and residues were removed after each test with 70% ethanol. Mouse activity and scoring in each test was automatically measured using ANY-maze behavioral tracking software, except for rotarod and beam walking tests, which were scored manually with the experimenter blinded to genotype. EPM, Y-maze, open field activity, and novel object recognition were performed as previously described (Afshari et al, 2017).

### Elevated plus maze

EPM is used to assess anxiety-like behavior. We used a standard mouse EPM apparatus from San Diego Instruments, which consists of two closed arms and two open arms perpendicular to one another, forming a “plus” shape. Mice were placed in the center of the apparatus, facing an open arm, and allowed to explore freely for 5 min under ambient light. The time spent in the open arms is reported to reflect anxiety-like behavior, such that the less time in the open arms, the more anxiety-like the behavior is.

### Y-maze spontaneous alternation test

We used the Y-maze spontaneous alternation test to assess spatial working memory. A custom-built apparatus was used, which consisted of three walled arms 16 inches long that are angled 120^o^ from one another. Mice were placed in the center and allowed to freely explore for 5 min in dim light. This test is based on the rodent’s tendency to explore new environments. Exploring the three arms consecutively, without reentry into an arm, is called a triad. This implies that the mouse “remembers” which arm it was most recently in, despite all arms appearing identical. To control for difference in total number of arm entries, we report Fraction of Alternation, which is (total number of triads)/(total number of arm entries – 2). Mice with less than five total arm entries were excluded from Fraction of Alternation analysis.

### Open field activity test

Open field activity testing was performed to assess spontaneous locomotor activity and anxiety-like behavior. We used a standard apparatus from San Diego Instruments, which consists of 50 × 50 cm open field surrounded by nontransparent walls. Mice were allowed to explore freely for 10 min in ambient light. The total time spent away from the predesignated “center zone” is reported to reflect anxiety-like behavior.

### Novel object recognition test

Novel object recognition testing was performed to assess long-term object recognition memory and was performed in the open field apparatus. Briefly, two identical objects (cubes) were placed in the chamber and the mice were allowed to explore the objects for 5 min per day in dim lighting for two training days. On the third day, a novel object (cylindrical piece of wood) replaced one of the cubes, and mice were allowed to explore the chamber for 10 min. If the mice remember the objects from the training days, then they tend to spend more time exploring the novel object. Reported is the time spent interacting with the novel object on the final day of testing. Mice with less than 5 s of total object exploration time on the final testing day were excluded from discrimination index analysis.

### Morris water maze

Morris water maze was performed to assess long-term spatial memory and learning, and was performed in an inflatable hot tub with the water temperature at 26°C in dim lighting, essentially as previously described (Vorhees & Williams, 2006). Briefly, mice were placed in the circular pool for four trials per day and allowed 1 min to find a platform that they can stand on. Platform was made of transparent plastic and placed just beneath the water surface as to make it invisible to the rodents. For pretraining day 1, mice were placed on the platform for 1 min, followed by placement of the mouse proximally to the platform so it learns it can escape the water. On pretraining day 2, mice were placed in a pseudorandomized quadrant of the pool and allowed 1 min to escape to the platform, which was made visible on this day with a small flag. Then, for seven training days, the platform was below the surface with no flag, and the mice were given four trials per day to learn where the platform was using spatial cues from around the room. On the probe day, the day after training day 7, the platform was removed, and the amount of time the mice spent swimming in the quadrant of the pool that previously had the platform, as well as the number of times the mice entered the platform zone, was recorded. The better long-term spatial memory the rodent has, the more time it spends in the target quadrant, and the more time it will enter the platform zone.

### Puzzle box test

To assess executive function, we performed the puzzle box test as previously described (Ben Abdallah et al, 2011; O’Connor et al, 2014). Briefly, mice were placed in a custom-built 60 × 28 cm apparatus that is well-lit with a small fan blowing into it to create a stressful environment with multisensory stimuli. The mice are given a passage (5 by 5 cm doorway) to small and dark target location filled with home bedding to make the mice comfortable. The idea is to test the mouse’s ability to “problem-solve” by removing obstacles between the stressful environment and the target location. In condition 0, there is no obstacle. Condition 1 had a U-shaped channel within the doorway. Condition 2 had the same U-shaped channel within the doorway, but with a mound of cage bedding placed in it to block the doorway. Condition 3 had a crumpled piece of paper placed in the doorway. Finally, condition 4 had a cube placed in the doorway, with a raised edge on top of it such that the mice had to pull it out of the doorway and could not push it through. We manually recorded the time it took to reach the target location and plotted that as “escape latency.” A maximum of 5 min was allowed for each trial.

### Beam walking test

The beam walking test was used as a measure of balance and coordination. Although typically the primary readout for this assay is time to traverse the beam, analysis of these data was compromised by the substantially increased locomotor activity of transgenic α-syn^A53T^ mice. Thus, we prioritized the number of times each mouse’s hindlimbs slipped off the beam as the primary readout for balance and coordination.

We custom-built a beam walking apparatus, consisting of a 1-m-long wooden beam suspended ∼16 inches above the floor. The start end of the beam was suspended in the air without an escape route, and the other end has the target box for the mice to escape into. The target box is an enclosed black cube of about 9 inches on each side. Before each test, home bedding from the tested mouse is added to the escape box for enticement. Aversive stimuli (a bright LED light and a small fan) were placed at the start end of the beam such that the escape box was the only route to protection. Mice were trained on an easy beam (0.5-inch rectangle) over 2 d. In training, if a mouse stopped moving while on the beam, it was gently prodded by the experimenter. On training day 1, the mouse was placed directly in the target box for 1 min, then placed on the beam two inches from the target box and allowed to escape to the target box, and finally placed at the far end of the beam for full traverse of the beam. On training day 2, each mouse was given three more trials on the easy beam. On test day, each mouse was tested twice on 0.5-inch circular and 0.25-inch rectangular beams. The number of hindlimb slips per beam was manually scored by an experimenter who was blinded to genotype. Plotted in Fig 3 is the average number of slips of the two trials.

### Optomotor response test

Optomotor acuity and contrast sensitivity of mice were determined by observing their optomotor behavior responses to a rotating sine-wave grating stimulus using the OptoMotry system (Prusky et al, 2004) as described previously (Umino et al, 2006, 2008). Briefly, mice were placed on a pedestal at a center of the OptoMotry chamber enclosed by four computer monitors. The computer randomly displayed sinusoidal pattern gratings rotating in a clockwise or counterclockwise direction. The observer was blind to the direction of rotation and chose the direction of pattern rotation based on the animal’s behavior. Auditory feedback indicated to the observer whether the selected direction was correct or incorrect. Using a staircase paradigm, the computer program controlled the spatial frequency and contrast of the stimulus. The threshold was set at 70% correct responses. Contrast sensitivity was defined as the reciprocal of the threshold contrast value and was measured at 1.5 Hz and a spatial frequency of 0.128 cycles/degree. Acuity was measured at a speed of rotation of 12 degrees/s and 100% contrast. All measurements were performed at the unattenuated maximal luminance of the OptoMotry system (∼70 cd/m^2^, producing ∼1,500 R*/rod/s [Bushnell et al, 2016]).

### Quantification and statistical analysis

Statistical analyses were performed using GraphPad Prism. For behavioral tests, we always probed for a statistically significant effect of genotype, sex, and genotype × sex interactions. If data are presented without separating sex, this indicates there were no significant main effect of sex and no significant genotype × sex interaction. For details on statistical testing of specific data, please see Figure Legends.

## Supplementary Material

Reviewer comments

## Data Availability

The RNA-seq and proteomic datasets generated as part of this study are presented as supplemental tables. The data have also been deposited to NCBI Gene Expression Omnibus/Sequence Read Archive with the accession number GSE236975. The mass spectrometry proteomics data have been deposited to the ProteomeXchange Consortium via the PRIDE [1] partner repository with the dataset identifier PXD043657 (DOI: 10.6019/PXD043657). There was no code generated as part of this study. The code sets in the Jupyter Notebook used in RNA-seq deconvolution are available upon request to corresponding author F Middleton.
