# Supplementary material for: Mitochondrial protein import stress augments α-synuclein aggregation and neural damage
Source: Life Sci Alliance. 2026 Nov 10;10(1):e202603770. doi: 10.26508/lsa.202603770 (PMC13752406; doi:10.26508/lsa.202603770)
Supplement: Supplementary file 1 [file LSA-2026-03770_SdataFS12.pdf]

Fig S12A, Spinal cord.

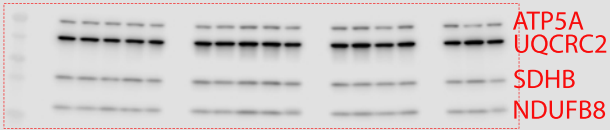

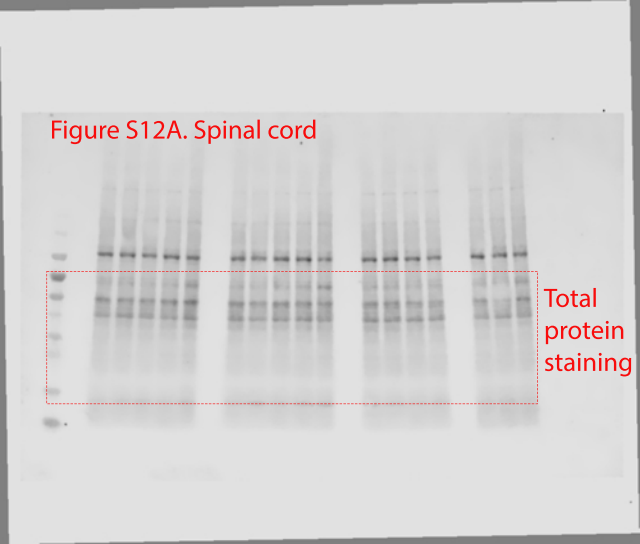
Figure S12A. Spinal cord
Total protein staining

Figure S12C, Forebrain

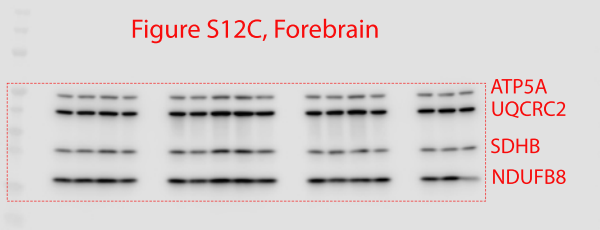

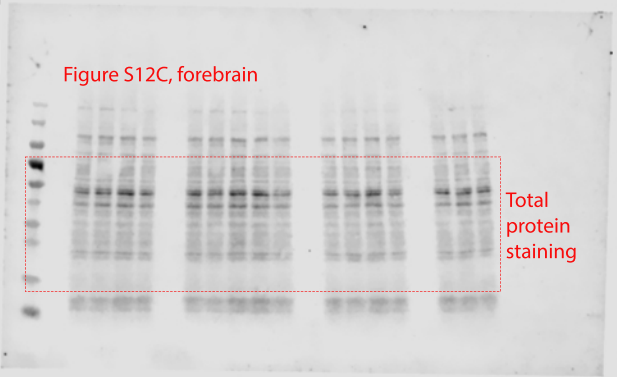

Figure S12C, forebrain
Total
protein
staining

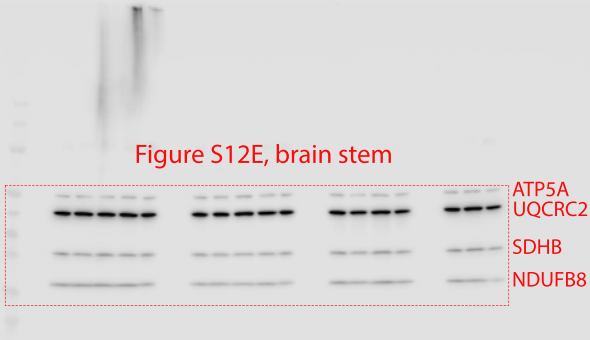
Figure S12E, brain stem
ATP5A
UQCRC2
SDHB
NDUFB8

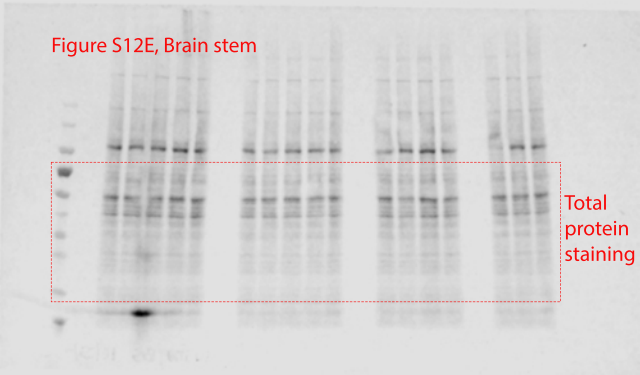
Figure S12E, Brain stem
Total protein staining

Figure S12G, Cerebellum

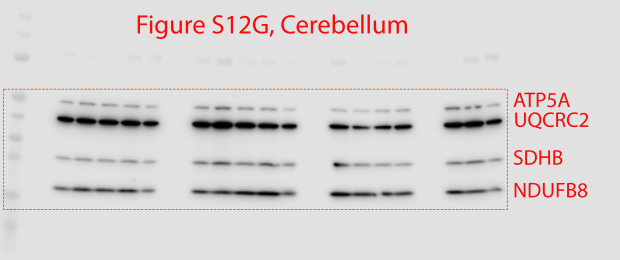

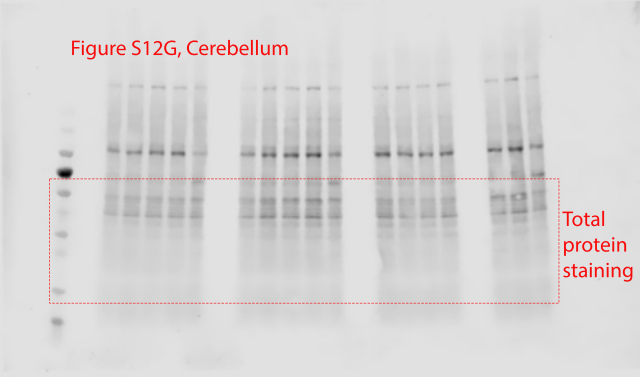
Figure S12G, Cerebellum
Total
protein
staining
