# Supplementary material for: Mitochondrial protein import stress augments α-synuclein aggregation and neural damage
Source: Life Sci Alliance. 2026 Nov 10;10(1):e202603770. doi: 10.26508/lsa.202603770 (PMC13752406; doi:10.26508/lsa.202603770)

LI-COR®

Image ID: 0008410_01
Acquire Time: Oct 19, 2021 12:20:04 PM

Acquisition Information

#	Image ID	Acquire Time	Channels	Integration Times	Analysis	Image Name	Comment
1	0008410_01	Oct 19, 2021 12:20:04 PM	Chemi 700	02:00 00:30	Manual	10-14-21_TPS_10-19-21<2>_FEMTO#2	

Image Display Values

Channel	Color	Minimum	Maximum	K
Chemi	Gray Scale (Black on White)	0.000346	0.00301	0
700	Gray Scale (Black on White)	0.00396	0.0118	0

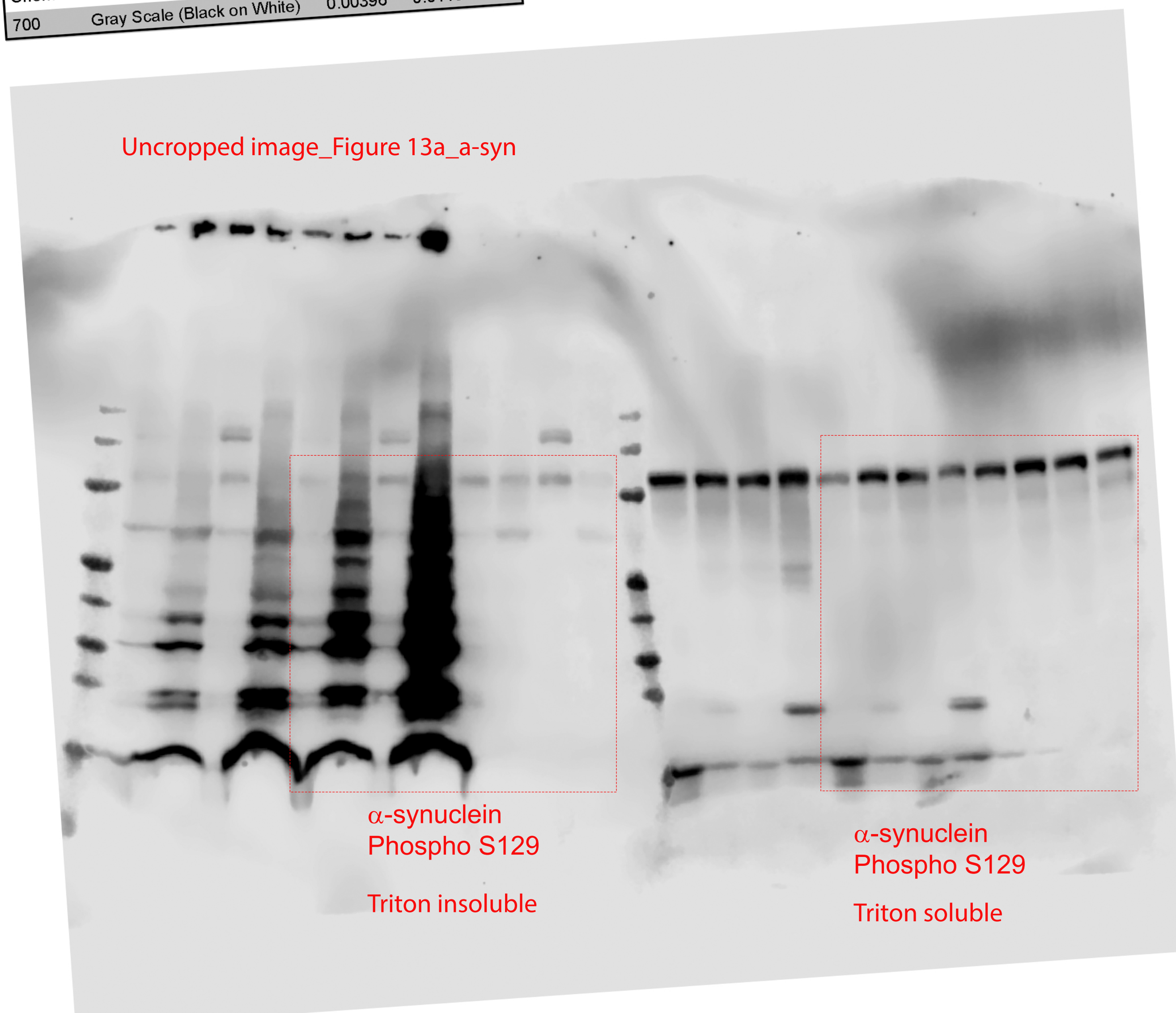

LI-COR®

Image ID: 0008454_01

Acquire Time: Oct 26, 2021 11:24:27 AM

Acquisition Information

#	Image ID	Acquire Time	Channels	Integration Times	Analysis	Image Name	Comment
1	0008454_01	Oct 26, 2021 11:24:27 AM	Chemi 700	02:00 00:30	Manual	10-14-21_B-actin_10-26-21<1>_ECL	

Image Display Values

Channel	Color	Minimum	Maximum	K
Chemi	Gray Scale (Black on White)	0.000176	0.0956	0
700	Gray Scale (Black on White)	0.00546	0.0381	0

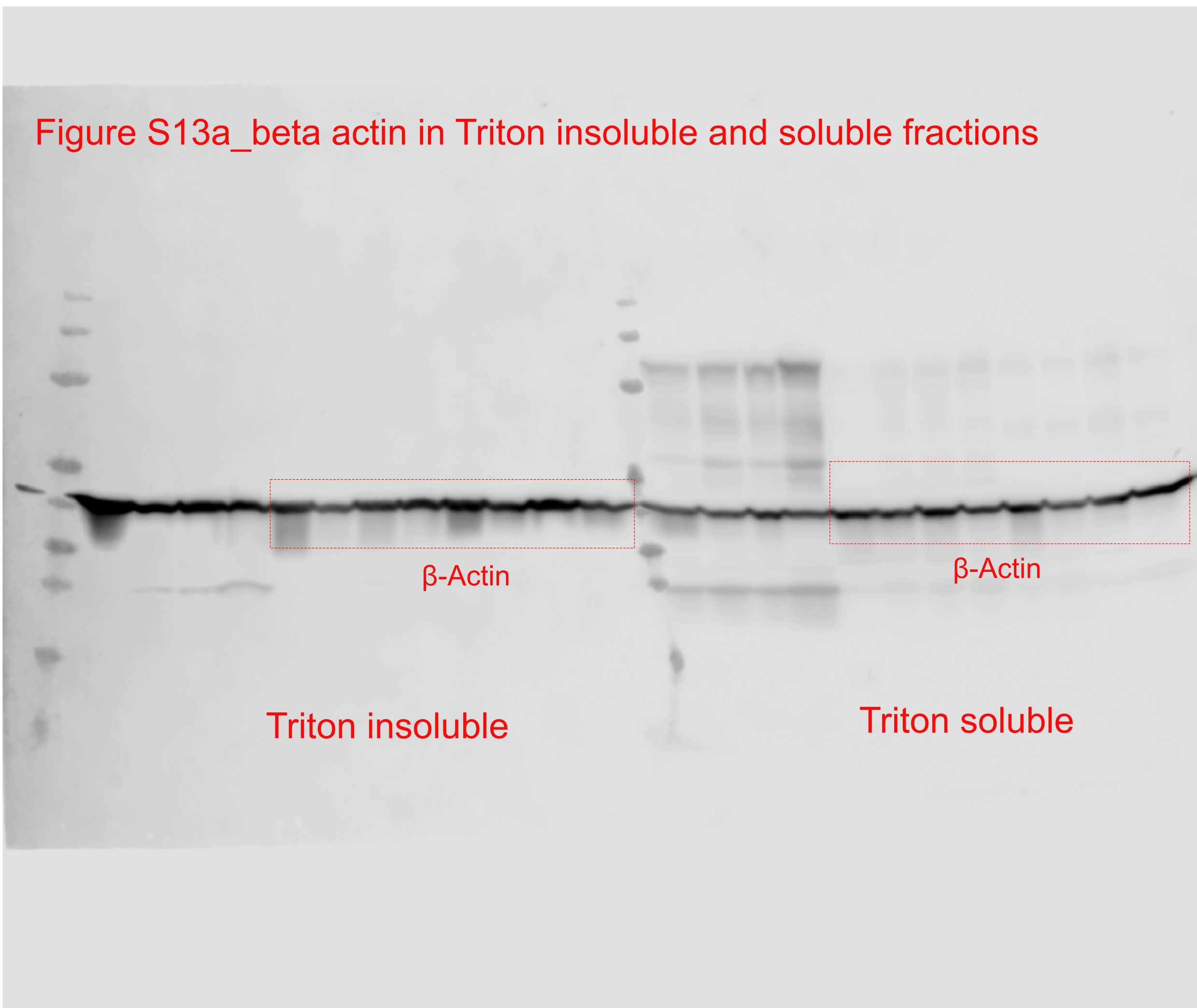

Supplement: Supplementary file 2 [file LSA-2026-03770_SdataFS13.pdf]
