# Supplementary material for: Mitochondrial protein import stress augments α-synuclein aggregation and neural damage
Source: Life Sci Alliance. 2026 Nov 10;10(1):e202603770. doi: 10.26508/lsa.202603770 (PMC13752406; doi:10.26508/lsa.202603770)

Figure S14A, OXPHOS cocktail

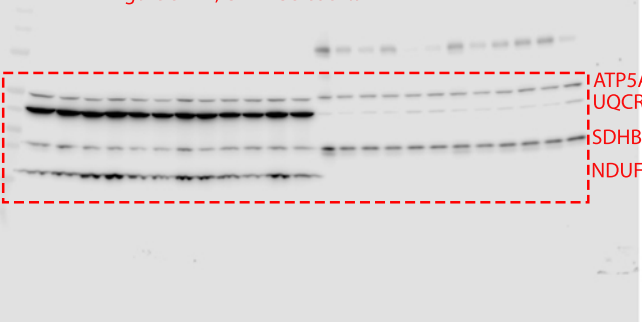

Figure S14A, VDAC

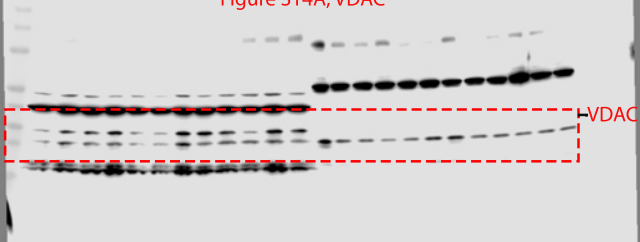

Figure S14A, Total protein staining

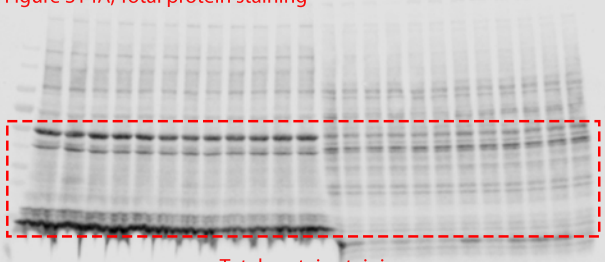

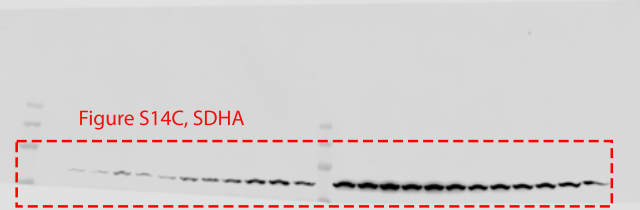
Figure S14C, SDHA

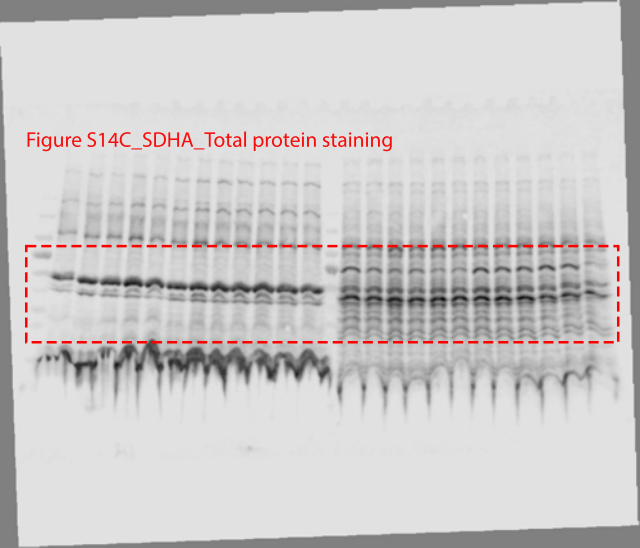
Figure S14C_SDHA_Total protein staining

Figure S14E, TIMM23

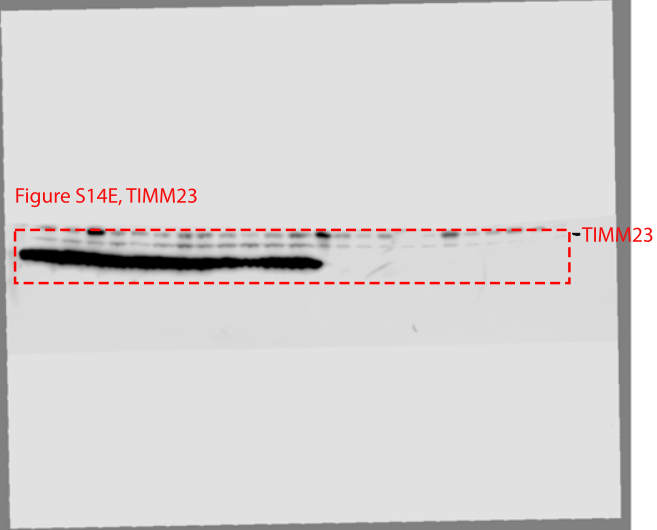

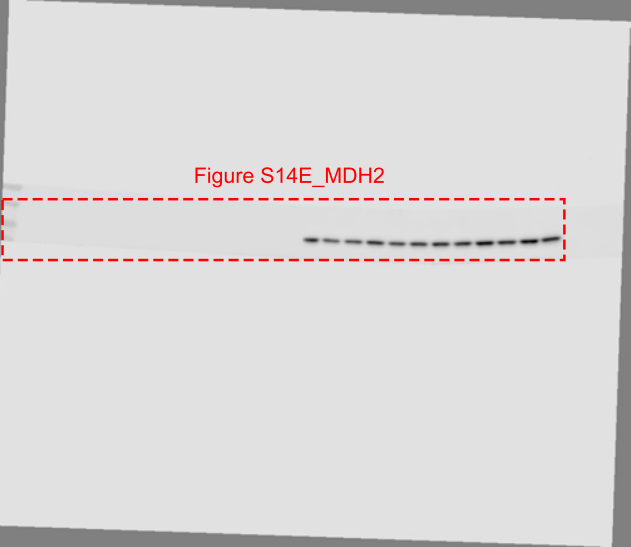
Figure S14E_MDH2

Figure S14E_TIMM23_MDH2_TPS

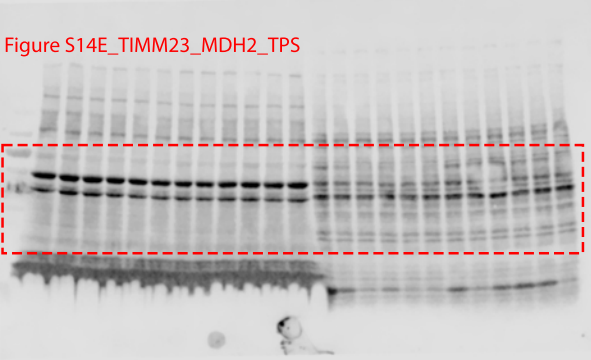

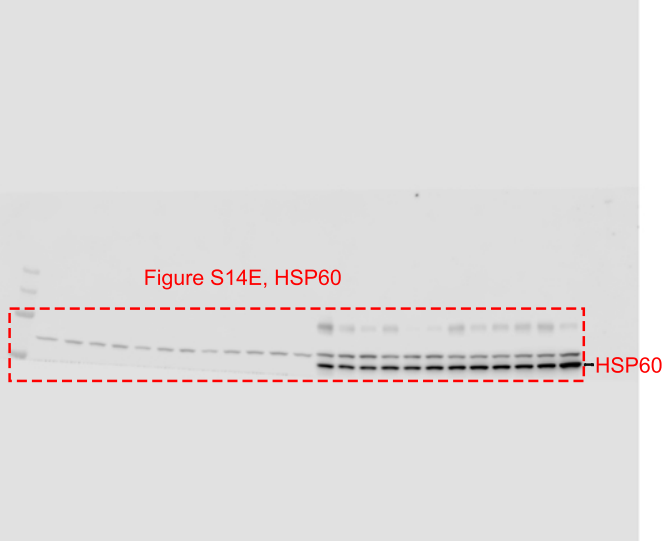
Figure S14E, HSP60
HSP60

Figure S14E_HSP60P_TPS

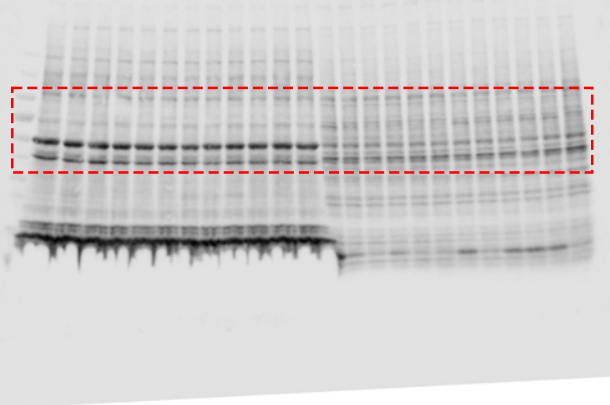

Supplement: Supplementary file 3 [file LSA-2026-03770_SdataFS14.pdf]
