# Supplementary material for: Mitochondrial protein import stress augments α-synuclein aggregation and neural damage
Source: Life Sci Alliance. 2026 Nov 10;10(1):e202603770. doi: 10.26508/lsa.202603770 (PMC13752406; doi:10.26508/lsa.202603770)
Supplement: Supplementary file 4 [file LSA-2026-03770_review_history.pdf]

Mitochondrial protein import stress augments alpha-synuclein aggregation and neural damage

Liam Coyne, Arnav Rana, Xiaowen Wang, Joseph Mauro, Sanaea Bhagwagar, Yumiko Umino, Eduardo Solessio, Frank Middleton, and Xin Jie Chen
DOI: https://doi.org/10.26508/lsa.202603770

Corresponding author(s): Xin Jie Chen, SUNY Upstate Medical University and Frank Middleton, SUNY Upstate Medical University

Review Timeline:

Submission Date:	2026-05-13
Editorial Decision:	2026-06-17
Revision Received:	2026-08-17
Editorial Decision:	2026-10-13
Revision Received:	2026-10-20
Accepted:	2026-10-23

Scientific Editor: Sarita Hebbar

Transaction Report:

(Note: With the exception of the correction of typographical or spelling errors that could be a source of ambiguity, letters and reports are not edited. The original formatting of letters and referee reports may not be reflected in this compilation.)

June 16, 2026

Re: Life Science Alliance manuscript #LSA-2026-03770

Prof. Xin Jie Chen
SUNY Upstate Medical University
Biochemistry and Molecular Biology
750 East Adams Street
Syracuse, New York 13210

Dear Dr. Chen,

Thank you for submitting your manuscript entitled "Mitochondrial protein import stress augments -synuclein aggregation and neural damage" to Life Science Alliance. Your manuscript was evaluated by three expert reviewers whose comments are included below. As you will note, all the reviewers have found the work of potential value with Reviewers 1 and 2 commenting positively on the study. That said, we agree with Reviewers 2 and 3 on the technical and conceptual concerns around the claims in this work. We invite you to submit a revised manuscript addressing these concerns:

-We agree with Reviewers 2 and 3 that you must extend your transcriptomics data set with further analyses and validation. We leave the choice of analyses and validation to you (Reviewer 2, points 2, 3 and Reviewer 3, point 3).
-We bring your attention to the point of Reviewer 3, point 4, and agree that you must provide complete information on sample sizes, replicate information and address their concern on accurate reporting and statistical analyses.
-We concur with Reviewer 2 that a revised manuscript must include a quantification of respiratory complex proteins (point 1) and provide representative images of astrocyte morphology (point 4). We leave it to your choice to provide additional staining on inflammation. This reviewer also makes an interesting case for co-aggregation of mitochondrial precursor proteins and a-synuclein in the detergent insoluble faction. We don't expect you to provide additional evidence (point 5) but you must classify this possibility as speculative whist discussing these results.
Finally we agree that a revised version must reflect the concerns of Reviewer 3 (points 1 and 2) in moderating and accurately stating specific claims (related to Figures S4A-B, Figure S8). You must reframe the discussion to indicate gaps in the supporting evidence (aggregation versus increased size of α-synuclein-positive puncta AND teasing the role of mutant ANT1 versus mitochondrial import defects in described phenotypes).

I would be happy to discuss the revision in more detail via email or phone/videoconferencing. Please let me know which option you prefer, if any.

To upload the revised version of your manuscript, please log in to your account: https://lsa.msubmit.net/cgi-bin/main.plex

You will be guided to complete the submission of your revised manuscript and to fill in all necessary information. Please get in touch in case you do not know or remember your login name.

The typical timeframe for revisions is three months. Please note that papers are generally considered through only one revision cycle, so strong support from the referees on the revised version is needed for acceptance.

While you are revising your manuscript, please also attend to the below editorial points to help expedite the publication of your manuscript. Please direct any editorial questions to the journal office. When submitting the revision, please include a letter addressing the reviewers' comments point by point.

Thank you for this interesting contribution to Life Science Alliance. We hope that the comments below will prove constructive as your work progresses, and we are looking forward to receiving your revised manuscript.

Sincerely,

Sarita Hebbar, PhD
Scientific Editor
Life Science Alliance
http://www.lsajournal.org

A. THESE ITEMS ARE REQUIRED FOR REVISIONS

-- A letter addressing the reviewers' comments point by point.

-- An editable version of the final text (.DOC or .DOCX) is needed for copyediting (no PDFs).

-- High-resolution figure, supplementary figure and video files uploaded as individual files: See our detailed guidelines for preparing your production-ready images, https://www.life-science-alliance.org/authors

-- Summary blurb (enter in submission system): A short text summarizing in a single sentence the study (max. 200 characters including spaces). This text is used in conjunction with the titles of papers, hence should be informative and complementary to the title and running title. It should describe the context and significance of the findings for a general readership; it should be written in the present tense and refer to the work in the third person. Author names should not be mentioned.

-- By submitting a revision, you attest that you are aware of our payment policies found here: https://www.life-science-alliance.org/copyright-license-fee

B. MANUSCRIPT ORGANIZATION AND FORMATTING:

Full guidelines are available on our Instructions for Authors page, https://www.life-science-alliance.org/authors

We encourage our authors to provide original source data, particularly uncropped/-processed electrophoretic blots and spreadsheets for the main figures of the manuscript. If you would like to add source data, we would welcome one PDF/Excel-file per figure for this information. These files will be linked as supplementary "Source Data" files.

IMPORTANT: It is Life Science Alliance policy that if requested, original data images must be made available. Failure to provide original images upon request will result in unavoidable delays in publication. Please ensure that you have access to all original microscopy and blot data images before submitting your revision.

Reviewer #1 (Comments to the Authors (Required)):

Mitochondrial dysfunction can be associated with defects in muscle cells and neurons and contribute to neurodegeneration. Particularly in Parkinson's disease (PD) the link between mitochondria and neurodegeneration is obvious, but the underlying mechanisms are not entirely clear. There is good evidence that mitochondrial dysfunction has a negative influence on proteostasis, potentially contributing to the aggregation of alpha-synuclein in dopaminergic neurons. In this study, the authors used mouse models for mitochondrial dysfunction and PD. They expressed a mutated version of the ATP/ADP translocator ANT (Slc25a4 pA114P,A123D) and showed that this did not significantly affect mitochondrial respiration. The mice showed an increased fitness and mental performance (on basis of tests for cognitive function). Next, the authors combined these mutations with a PD model expressing a mutated version of alpha-synuclein. Again, mitochondria were unaffected in these mice in respect to respiration. Moreover, the toxicity of the alpha-synuclein was obvious (Fig. S4B) but only slightly aggravated by the additional expression of the ANT mutant. However, the authors described an increased appearance of aggregates in cells of the double mutant which contain mitochondrial proteins, suggesting that the combination of the alpha-synuclein mutant and mitochondrial dysfunction challenges (cytosolic) proteostasis in this model. The study is clear and data rich.
I already reviewed this study for another journal, and the authors had satisfactorily addressed all points that I had raised on the initial version. I therefore support the publication of this interesting study in its present form.

Reviewer #2 (Comments to the Authors (Required)):

The manuscript by Coyne et al. aims to disentangle the role of mitochondrial import stress from other mitochondria-related pathogenic mechanisms, such as bioenergetic defects, in the context of Parkinson's disease (PD). In previous work, the authors developed an elegant genetic strategy to induce mild clogging of the mitochondrial import machinery in mice. This was achieved by introducing point mutations in the mitochondrial protein Slc25a4, which leads to partial clogging of the TOM import complex by the mutant protein. In the present study, crossing this "clogger mouse" line to the a-synuclein A53T model of PD allowed the authors to investigate the contribution of impaired mitochondrial precursor import to synucleinopathy phenotypes. The authors analyzed multiple disease signatures and found synergistic effects of mitochondrial import clogging and a-synuclein mutation in some of the disease phenotypes. In particular, they observed aggravation of aggregate burden, neuroinflammation and motor defects, in the absence of bioenergetic impairments. The study thereby builds a bridge between protein aggregation and mitochondrial defects, two key hallmarks of neurodegeneration that are well-established on their own, but the connection between them is poorly understood.
The experiments are thoroughly done, and the paper well written. While the study is overall interesting and novel, several aspects need improvement.

Major comments:
1. The protein levels of respiratory complex proteins shown in Fig. 1I should be quantitatively analyzed.
2. In the spinal cord transcriptomic data (Fig. 2A-B), in addition to highlighting single upregulated genes, the authors should perform pathway analysis for a more holistic picture of transcriptional dysregulation in the clogger mice.
3. The transcriptomic data needs validation on the protein level. For example, the authors suggest a coordinated stress response via the IGF2/IGFBP6 axis. The upregulation should be confirmed by immunostaining and/or Western blot.
4. The quantification of neuroinflammation via GFAP staining is currently insufficient to support the claim of increased inflammation. Please provide representative images with higher magnification insets to show astrocyte morphology. Furthermore, given the involvement of microglia in synucleinopathies, it is necessary to include Iba1 staining to characterize the inflammatory landscape of the spinal cord.
5. The authors demonstrate that mitochondrial precursor proteins and a-synuclein accumulate together in the detergent-insoluble fraction. This suggests a possible co-aggregation, which is a very exciting finding. To strengthen this point, additional evidence is necessary, either by co-immunoprecipitation or Proximity Ligation Assay (PLA) in the spinal cord tissue.

Minor points:
6. The authors interpret the lack of transcriptional changes in antioxidant genes as evidence of the absence of oxidative stress. This is an overstatement. I recommend either performing a direct biochemical assay for oxidative stress or rephrasing to acknowledge that transcriptome changes do not reflect function.
7. In Fig. S4, please outline the border of the grey matter in the spinal cord and point to some examples of motor neurons.
8. In the abstract, please clarify that mitochondrial import stress was induced by a genetic mutation in Slc25a4 / Ant1, otherwise it is not clear what "single or double mutant mice" refers to.
9. P. 13, line 263: "stromal size" should be "soma size".

Reviewer #3 (Comments to the Authors (Required)):

Protein aggregation and mitochondrial dysfunction are hallmarks of many age-related neurodegenerative disorders, including Parkinson's disease (PD). In this study, Coyne and colleagues investigate whether mild mitochondrial protein import stress contributes to PD. To do so, they repurpose a previously established mouse model expressing a mutant version of the mitochondrial ADP/ATP carrier SLC25A4 (ANT1), which partially clogs the mitochondrial import machinery. Prior work showed that this mutant causes accumulation of unimported mitochondrial proteins in the cytosol and mild mitochondrial dysfunction in skeletal muscle (PMID: 7129366).
Here, the authors examined the effects of this mutation in the central nervous system (CNS) and found evidence of a mild proteotoxic stress response without detectable impairment of mitochondrial respiration. They then crossed these mice with α-synuclein(A53T) transgenic anamals, a model of Parkinson's disease. Expression of the clogger mutant increased the size of phosphorylated α-synuclein punctae, enhanced neuroinflammation, and worsened motor coordination deficits, while mitochondrial bioenergetic function remained largely unchanged. Together, the authors propose a model in which mitochondrial protein import stress promotes α-synuclein pathology through disruption of cytosolic proteostasis, independent of overt mitochondrial bioenergetic defects.
While the hypothesis that mitochondrial protein import stress aggravates α-synuclein pathology is intriguing and potentially important, several central conclusions are not sufficiently supported by the presented data. In addition, substantial issues with figure presentation and statistical reporting limit the interpretability of the study. Therefore, substantial revision is required before the manuscript can be considered for publication.

Major comments
1. The central premise of the manuscript is that ANT1A114P,A123D induces mitochondrial protein import stress in the CNS without impairing mitochondrial bioenergetics. However, the authors do not directly assess mitochondrial protein import clogging in neural tissues. Consequently, the conclusion that mitochondrial protein import stress promotes α-synuclein pathology in the CNS relies largely on extrapolation from previous observations in skeletal muscle and other model systems. Whether such extrapolation is justified remains however unclear. The authors acknowledge that the mutant ANT1 protein is highly unstable and present at very low levels, limiting their ability to assess import clogging in the CNS. Indeed, the transcriptomic analyses provide only indirect support for a mitochondrial protein import stress response, and the observed transcriptional changes appear relatively modest. Furthermore, the authors do not examine the insoluble proteome in the CNS of clogger mice in the absence of α-synuclein expression, which could have provided independent evidence for disturbed proteostasis resulting from impaired protein import.
As a result, a substantial gap remains between the proposed mechanism and the presented data. At present, it is not possible to determine whether the observed phenotypes arise from mitochondrial protein import stress itself or from other consequences of expressing the mutant ANT1 protein. Additional evidence directly demonstrating impaired mitochondrial protein import in the CNS of clogger mice is therefore required to support the central conclusions of the study.

2. The authors provide convincing evidence that expression of ANT1A114P,A123D worsens beam-walking performance and increases neuroinflammatory markers in the α-synuclein(A53T) mouse model. However, the evidence that these phenotypes are

driven by enhanced α-synuclein aggregation is less compelling. Specifically, while the immunofluorescence analyses in Figure 5 demonstrate an increased size of phosphorylated α-synuclein-positive puncta in double mutant animals, this does not necessarily demonstrate increased α-synuclein aggregation. Notably, the biochemical evidence provided in Figure S8B-C is weak as differences are subtle, appear to rely heavily on quantification, and are difficult to appreciate directly from the immunoblots. Moreover, the observation that oligomeric species are predominantly detected in the Triton-soluble fraction is somewhat unexpected for aggregation-prone α-synuclein species and warrants further explanation.

In addition, many of the reported pathological differences are only observed in end-stage paralyzed animals and are largely absent in non-paralyzed mice. This raises the possibility that ANT1A114P,A123D does not substantially accelerate α-synuclein aggregation during disease progression, but rather exacerbates pathology after the onset of severe neurodegeneration. The current data therefore do not clearly distinguish between a direct effect on α-synuclein aggregation and a more general worsening of late-stage disease. Therefore, additional analyses are required to strengthen the proposed mechanism. For example, assessment of Lewy body-like pathology, ultrastructural analysis by electron microscopy, evaluation of additional brain regions, and further biochemical characterization of α-synuclein aggregates in non-end-stage mice would provide more direct evidence that ANT1A114P,A123D enhances α-synuclein aggregation in vivo.

Finally, several conclusions appear stronger than warranted by the presented data. For example, the statement that ANT1A114P,A123D increases the loss of VAChT-positive motor neurons relative to α-synuclein(A53T) mice is not supported by the data shown in Figure S4A-B, where the reported differences are not apparent from the presented data and do not appear to reach statistical significance.

3. The transcriptomic and proteomic analyses constitute an important component of the mechanistic framework proposed by the authors, yet several aspects of these datasets remain difficult to evaluate. Most importantly, sample sizes and biological replication are not always clearly stated, and in several figures the statistical analyses are either absent or insufficiently described (see also point 4). Given that many of the reported changes are relatively modest, it is difficult to assess the robustness and biological significance of the findings.

In addition, several key conclusions rely heavily on large-scale omics datasets without independent validation. For example, the proposed activation of proteostatic stress pathways and proteasomal responses would be substantially strengthened by orthogonal validation of selected targets using quantitative PCR, immunoblotting, or other targeted approaches. Likewise, validation of proteins identified in the insoluble proteome would help establish the biological relevance of the proteomics findings.

4. The statistical analysis and reporting require substantial clarification.

First, male and female mice were pooled for most analyses. While this approach is acceptable when sex does not significantly influence the measured outcome, the study appears underpowered to rigorously assess sex-dependent effects. In several experiments, the number of animals per sex appears low and is not always clearly stated. Consequently, the absence of a statistically significant sex effect should not necessarily be interpreted as evidence that sex differences are absent. Under these circumstances, pooling male and female animals may obscure biologically meaningful sex-dependent effects and should therefore be justified with appropriate statistical analyses. This concern is particularly relevant because the authors report that "clogger mice demonstrate sex-dependent increases in locomotor activity, but no changes in anxiety-like behavior or memory". The authors should provide the sex-specific data and statistical analyses.

Second, the choice of statistical tests appears questionable in several instances and requires further justification. For example, the rationale for using repeated-measures ANOVA in Figures 1A-H, 1J, and 1K is unclear. Likewise, Figures 3E and 5B-D appear more appropriately analyzed using ANOVA models rather than multiple Student t-tests, given the underlying experimental design. The manuscript would also benefit from a clearer description of multiple-comparison correction procedures, which are not consistently reported.

Third, statistical reporting throughout the manuscript is incomplete. Several figures report only selected comparisons, while others appear to lack statistical analyses altogether. Whenever appropriate, the authors should report the key genotype comparisons, including WT vs. clogger, WT vs. α-synuclein(A53T), WT vs. α-synuclein(A53T)/clogger, and α-synuclein(A53T) vs. α-synuclein(A53T)/clogger. In addition, the reported p-values for Figures 3B-C appear inconsistent between the main text, figure panels, and figure legends. These discrepancies need to be resolved and all relevant statistical comparisons should be displayed directly in the figures.

Finally, the number of biological replicates is not always apparent. In several figures, including Figure 2C and Figure 5E-F, it is unclear whether individual data points represent biological replicates or technical measurements. Likewise, the number of samples included in the transcriptomic and proteomic analyses should be stated explicitly in both the Methods and figure legends.

5. Numerous figure labels appear misaligned or displaced, in some cases preventing interpretation of the underlying data. This issue affects several main and supplementary figures and must be corrected before the manuscript can be adequately evaluated.

Minor Comments:

- The authors should label the up/downregulated transcripts/pathways in the heatmap in Figure 2A.
- How do the authors explain that α-synuclein mice have higher anxiety scores (Figure S5A) but behave normally in the edge/center zone analysis (Figure S5B). Along these lines, why is anxiety not affected by double mutant in Figure S5A compared to α-synuclein mice but in Figure S5B there is a difference. Why is this difference isolated to the middle distances?

• Table S4 is hard to interpret as UniProt identifiers instead of gene symbols are provided. It is also unclear what the ratio A/D means.
• All tables lack supporting information and the corresponding raw data.

Response to Reviewers Comments

We would like first to thank the reviewers for taking time to evaluate our work, and the editor for crafting out the revision plan of the manuscript. We are pleased to learn that reviewers found our study novel, thorough and potentially important for better understanding how mitochondrial stress could contribute to Parkinson's disease. The manuscript is extensively revised, with the new experiments and revisions highlighted in the manuscript text file. Below is the list of new data added to the revised manuscript, and a summary of point-to-point responses to reviewer's comments and suggestions.

New data:

- Figure 2C - GSEA analysis revealing leading edge genes activated in the spinal cord of clogger mice compared with the wild type at 24- and 30 months of ages.
- Figure S1 - Western blot analysis showing the integrity of the OXPHOS pathway, using a cocktail of antibodies against the indicated mitochondrial proteins.
- Figure S4A - Real time quantitative reverse transcription PCR validating the activation of *Hspa1b*, *Igf2* and *Igfbp6* (n=4-5, Student's *t* test).
- Figure S5. Gene Set Enrichment Analysis (GSEA) of spinal cord RNA-seq data generated from 24- and 30-month-old Clogger versus wild type mice.
- Figure S6. Deconvolution analysis of bulk RNA-seq data showing the proportions of neural cell types.
- Figure S7. Whisker box plot of microglial gene expression changes in clogger vs WT mice.
- Figure S9: Ubiquitin inclusion bodies in midbrain regions.
- Figure S14: Distribution of a selected panel of mitochondrial proteins in Triton-soluble and insoluble fractions of spinal cord from end-stage a-syn and a-syn + clogger mice.

Reviewer #1:

Mitochondrial dysfunction can be associated with defects in muscle cells and neurons and contribute to neurodegeneration. Particularly in Parkinson's disease (PD) the link between mitochondria and neurodegeneration is obvious, but the underlying mechanisms are not entirely clear. There is good evidence that mitochondrial dysfunction has a negative influence on proteostasis, potentially contributing to the aggregation of alpha-synuclein in dopaminergic neurons. In this study, the authors used mouse models for mitochondrial dysfunction and PD. They expressed a mutated version of the ATP/ADP translocator ANT (Slc25a4 pA114P,A123D) and showed that this did not significantly affect

mitochondrial respiration. The mice showed an increased fitness and mental performance (on basis of tests for cognitive function). Next, the authors combined these mutations with a PD model expressing a mutated version of alpha-synuclein. Again, mitochondria were unaffected in these mice in respect to respiration. Moreover, the toxicity of the alpha-synuclein was obvious (Fig. S4B) but only slightly aggravated by the additional expression of the ANT mutant. However, the authors described an increased appearance of aggregates in cells of the double mutant which contain mitochondrial proteins, suggesting that the combination of the alpha-synuclein mutant and mitochondrial dysfunction challenges (cytosolic) proteostasis in this model. The study is clear and data rich. I already reviewed this study for another journal, and the authors had satisfactorily addressed all points that I had raised on the initial version. I therefore support the publication of this interesting study in its present form.

<u>*Response*</u> **–** *Thank you for taking time to evaluate our work*.

Reviewer #2:

The manuscript by Coyne et al. aims to disentangle the role of mitochondrial import stress from other mitochondria-related pathogenic mechanisms, such as bioenergetic defects, in the context of Parkinson's disease (PD). In previous work, the authors developed an elegant genetic strategy to induce mild clogging of the mitochondrial import machinery in mice. This was achieved by introducing point mutations in the mitochondrial protein Slc25a4, which leads to partial clogging of the TOM import complex by the mutant protein. In the present study, crossing this "clogger mouse" line to the a-synuclein A53T model of PD allowed the authors to investigate the contribution of impaired mitochondrial precursor import to synucleinopathy phenotypes. The authors analyzed multiple disease signatures and found synergistic effects of mitochondrial import clogging and a-synuclein mutation in some of the disease phenotypes. In particular, they observed aggravation of aggregate burden, neuroinflammation and motor defects, in the absence of bioenergetic impairments. The study thereby builds a bridge between protein aggregation and mitochondrial defects, two key hallmarks of neurodegeneration that are well-established on their own, but the connection between them is poorly understood.

The experiments are thoroughly done, and the paper well written. While the study is overall interesting and novel, several aspects need improvement.

<u>Major comments</u>:

1. The protein levels of respiratory complex proteins shown in Fig. 1I should be quantitatively analyzed.

<u>Response</u> – We reanalyzed the samples and quantified the respiratory complex proteins in the spinal cord. The data are now presented in Fig. S1. To further implement reviewer's comments, we extended our analysis to other CNS tissues including forebrain, brain stem and cerebellum.

2. In the spinal cord transcriptomic data (Fig. 2A-B), in addition to highlighting single upregulated genes, the authors should perform pathway analysis for a more holistic picture of transcriptional dysregulation in the clogger mice.

<u>Response</u> – To implement reviewer's comment, we performed GSEA analysis of the spinal cord RNA-seq data. We found that pathways involved in microglia phagocytosis, macrophage activation, JAK-STAT signaling pathway and complement and coagulation cascades are activated in the spinal cord of 24- and 30 month old of Ant1$^{P.A114P,A123D}$/+ mice (Figure S5). This supports heightened inflammation. In contrast, genes encoding proteins of mitochondrial envelope and membrane organization are downregulated, likely as a response to import clogging which reduces protein loading. More importantly, many genes involved in response to unfolded proteins are activated in 30-month-old Ant1$^{P.A114P,A123D}$/+ mice. Among the leading edge genes from the GSEA analysis, we found that those involved complement activity and phagocytic function are activated (Figure 2C) are activated. These data provide strong support for disturbed proteostasis and increased inflammation.

3. The transcriptomic data needs validation on the protein level. For example, the authors suggest a coordinated stress response via the IGF2/IGFBP6 axis. The upregulation should be confirmed by immunostaining and/or Western blot.

<u>Response</u> – As IGF2 and IGFBP6 are secreted proteins, immunostaining and/or Western blot analysis may not be the best way to validate the upregulation of these proteins. To overcome this, we used quantitative reverse PCR to show that these two genes, together with Hspa1b, are indeed upregulated in the spinal cord of the clogger mice. This validated the RNA-seq data, as presented in Figure S4A.

4. The quantification of neuroinflammation via GFAP staining is currently insufficient to support the claim of increased inflammation. Please provide representative images with higher magnification insets to show astrocyte morphology. Furthermore, given the

involvement of microglia in synucleinopathies, it is necessary to include Iba1 staining to characterize the inflammatory landscape of the spinal cord.

<u>Response</u> – *Representative images of higher magnification insets are added to Figure 3D allowing the visualization of astrocyte morphology.*

To detect microglia activation, we used a more sensitive method than Iba1 staining, by deconvoluting the bulk RNA-seq data. Interestingly, we found that the proportions of both microglia and astrocytes are already increased in the aged spinal cord from the clogger single mutant mice (Figure S6). Furthermore, expression of 15/25 microglial genes is significantly increased in the spinal cord, and more moderately, in the striatum of the clogger mice (Figure S7; Supplemental Table 4-5). Among these genes are TREM2 and CX3CR1 that are known to be preferentially expressed in microglia in Alzheimer's disease (Figure 2C). These data provide strong support for microglia activation in clogger mice that is relevant to neurodegenerative diseases.

5. The authors demonstrate that mitochondrial precursor proteins and a-synuclein accumulate together in the detergent-insoluble fraction. This suggests a possible co-aggregation, which is a very exciting finding. To strengthen this point, additional evidence is necessary, either by co-immunoprecipitation or Proximity Ligation Assay (PLA) in the spinal cord tissue.

<u>Response</u> – *Proteostasis is maintained by an interconnected proteostatic network in the cytosol, which is challenged by both the unimported mitochondrial proteins and the mutant alpha-synuclein. Unimported mitochondrial proteins could therefore co-aggregate with alpha-syn, or increase alpha-syn aggregation by titrating out molecular chaperones and proteases. As the unimported mitochondrial proteins may have different aggregation properties, it may not be feasible to capture the interaction between the aggregate of a specific protein with aggregated alpha-synuclein by Co-IP or PLA. But this is certainly a constructive suggestion that can be explored in futures studies. Being on the cautious side, as suggested by the editor, we leave the question open in the revised version as to whether co-aggregation occurs. The word "co-aggregation" was also removed from the abstract. Thank you!*

<u>Minor points</u>:

6. The authors interpret the lack of transcriptional changes in antioxidant genes as evidence of the absence of oxidative stress. This is an overstatement. I recommend either

performing a direct biochemical assay for oxidative stress or rephrasing to acknowledge that transcriptome changes do not reflect function.

<u>Response</u> – *Good point. We deleted the comment from the text.*

7. In Fig. S4, please outline the border of the grey matter in the spinal cord and point to some examples of motor neurons.

<u>Response</u> – Done.

8. In the abstract, please clarify that mitochondrial import stress was induced by a genetic mutation in Slc25a4 / Ant1, otherwise it is not clear what "single or double mutant mice" refers to.

<u>Response</u> – *Fixed*.

9. P. 13, line 263: "stromal size" should be "soma size".

<u>Response</u> – *Fixed*

Reviewer #3:

Protein aggregation and mitochondrial dysfunction are hallmarks of many age-related neurodegenerative disorders, including Parkinson's disease (PD). In this study, Coyne and colleagues investigate whether mild mitochondrial protein import stress contributes to PD. To do so, they repurpose a previously established mouse model expressing a mutant version of the mitochondrial ADP/ATP carrier SLC25A4 (ANT1), which partially clogs the mitochondrial import machinery. Prior work showed that this mutant causes accumulation of unimported mitochondrial proteins in the cytosol and mild mitochondrial dysfunction in skeletal muscle (PMID: 7129366).

Here, the authors examined the effects of this mutation in the central nervous system (CNS) and found evidence of a mild proteotoxic stress response without detectable impairment of mitochondrial respiration. They then crossed these mice with α-synuclein(A53T) transgenic anamals, a model of Parkinson's disease. Expression of the clogger mutant increased the size of phosphorylated α-synuclein punctae, enhanced neuroinflammation, and worsened motor coordination deficits, while mitochondrial bioenergetic function remained largely unchanged. Together, the authors propose a model

in which mitochondrial protein import stress promotes α-synuclein pathology through disruption of cytosolic proteostasis, independent of overt mitochondrial bioenergetic defects.

While the hypothesis that mitochondrial protein import stress aggravates α-synuclein pathology is intriguing and potentially important, several central conclusions are not sufficiently supported by the presented data. In addition, substantial issues with figure presentation and statistical reporting limit the interpretability of the study. Therefore, substantial revision is required before the manuscript can be considered for publication.

<u>Response</u> – *Thank you for the thorough review and insightful comments*!

Major comments

1. The central premise of the manuscript is that ANT1A114P,A123D induces mitochondrial protein import stress in the CNS without impairing mitochondrial bioenergetics. However, the authors do not directly assess mitochondrial protein import clogging in neural tissues. Consequently, the conclusion that mitochondrial protein import stress promotes α-synuclein pathology in the CNS relies largely on extrapolation from previous observations in skeletal muscle and other model systems. Whether such extrapolation is justified remains however unclear. The authors acknowledge that the mutant ANT1 protein is highly unstable and present at very low levels, limiting their ability to assess import clogging in the CNS. Indeed, the transcriptomic analyses provide only indirect support for a mitochondrial protein import stress response, and the observed transcriptional changes appear relatively modest. Furthermore, the authors do not examine the insoluble proteome in the CNS of clogger mice in the absence of α-synuclein expression, which could have provided independent evidence for disturbed proteostasis resulting from impaired protein import. As a result, a substantial gap remains between the proposed mechanism and the presented data. At present, it is not possible to determine whether the observed phenotypes arise from mitochondrial protein import stress itself or from other consequences of expressing the mutant ANT1 protein. Additional evidence directly demonstrating impaired mitochondrial protein import in the CNS of clogger mice is therefore required to support the central conclusions of the study.

<u>Response</u> – *We agree with reviewer that the transcriptomic analyses provide only indirect support for mitochondrial protein import stress and mPOS in the CNS tissues, and that a direct assessment of protein import clogging and insoluble proteome in the CNS of clogger mice would strengthen our conclusion. In the publication PMID: 7129366, we attempted as much as we could to document mitochondrial protein import clogging in the CNS in*

support of the neurodegenerative phenotype. However, given the low pathogenic penetrance of ANT1(A114P,A123D), the relative low expression of ANT1 in the CNS relative to the skeletal muscle, and the extremely unstable nature of the mutant clogger protein, the clogging phenotype is expected to be mild. It is unlikely that current tools available to us, including analysis of the cytosolic proteome by TMT-MS and determination of insoluble proteome, would be sensitive enough to produce quantitatively significant data. In the current study, we made good use of the overall weak phenotypes associated with the clogger mice to genetically dissect the proteostatic effects from bioenergetic defects, and to generate synergistic phenotypes (motor deficit, neuroinflammation, mitochondrial protein aggregation and alterations to alpha-syn dynamics) with the alpha-syn mice at otherwise asymptomatic stage. A severe clogger would make it difficult to achieve our goals. We share with reviewer's view that the transcriptomic data provides indirect evidence for mitochondrial protein import stress in the CNS. Accordingly, as suggested by the editor, we reframed the discussion section by pointing out the limitations of our study in this aspect. We also provide relevant background on the effect of the mutations causing mitochondrial protein import stress in yeast, cultured huma cells and skeletal muscle in mice, to support our assumption. We hope that this helps avoid overstatement of our data. We appreciate the comments and thank you.

On the other hand, to strengthen the transcriptomic data in CNS tissues of the clogger mice, we used GSEA analysis and deconvolution of bulk RNA-seq data (see above in response to reviewer 1's comments). Pathway analysis revealed the activation of a large group of genes encoding cytosolic heat chock proteins, consistent with increased response to proteostatic stress. Deconvolution of bulk RNA-seq data provided support for microglia and astrocyte activation. These new data provided additional evidence for cellular stress in the CNS of the clogger mice.

To provide additional evidence for a synergistic interaction between unimported mitochondrial proteins and the mutant alpha-syn, we examined the aggregation propensity of a selected panel of mitochondrial proteins in the alpha-syn single and alpha-syn + clogger mice. We found that SDHA, SDHB, VDAC and TIMM23 are significantly enriched in the Triton-insoluble spinal cord fractions from a-syn + clogger relative to the a-syn mice (Figure S14A-F). The presence of proteins such as SDHA in the soluble fraction is accordingly reduced in the a-syn plus clogger relative to the a-syn mice. On the other hand, several mitochondrial matrix proteins (MDH2 and HSP60) are undetectable in the Triton-insoluble fractions in a-syn and a-syn plus clogger mice (Figure S14E). We added these new data to the revised manuscript, further supporting the aggregation of unimported mitochondrial proteins in alpha-syn background and that this is likely dependent on the biochemical properties of individual proteins.

2. The authors provide convincing evidence that expression of ANT1A114P,A123D worsens beam-walking performance and increases neuroinflammatory markers in the α-synuclein(A53T) mouse model. However, the evidence that these phenotypes are driven by enhanced α-synuclein aggregation is less compelling. Specifically, while the immunofluorescence analyses in Figure 5 demonstrate an increased size of phosphorylated α-synuclein-positive puncta in double mutant animals, this does not necessarily demonstrate increased α-synuclein aggregation. Notably, the biochemical evidence provided in Figure S8B-C is weak as differences are subtle, appear to rely heavily on quantification, and are difficult to appreciate directly from the immunoblots. Moreover, the observation that oligomeric species are predominantly detected in the Triton-soluble fraction is somewhat unexpected for aggregation-prone α-synuclein species and warrants further explanation.
In addition, many of the reported pathological differences are only observed in end-stage paralyzed animals and are largely absent in non-paralyzed mice. This raises the possibility that ANT1A114P,A123D does not substantially accelerate α-synuclein aggregation during disease progression, but rather exacerbates pathology after the onset of severe neurodegeneration. The current data therefore do not clearly distinguish between a direct effect on α-synuclein aggregation and a more general worsening of late-stage disease. Therefore, additional analyses are required to strengthen the proposed mechanism. For example, assessment of Lewy body-like pathology, ultrastructural analysis by electron microscopy, evaluation of additional brain regions, and further biochemical characterization of α-synuclein aggregates in non-end-stage mice would provide more direct evidence that ANT1A114P,A123D enhances α-synuclein aggregation in vivo.
Finally, several conclusions appear stronger than warranted by the presented data. For example, the statement that ANT1A114P,A123D increases the loss of VAChT-positive motor neurons relative to α-synuclein(A53T) mice is not supported by the data shown in Figure S4A-B, where the reported differences are not apparent from the presented data and do not appear to reach statistical significance.

<u>Response</u> – *We examined the ubiquitin positive inclusion bodies in the midbrain regions which has been reported in this particular mouse model overexpressing human a-syn A53T. Within the midbrain reticular nucleus and the superior colliculus (two regions responsible for the initiation of movement) we observed a mild but statistically insignificant increase in inclusion body formation in a-syn + Ant1$^{p.A114P,A123D}$ mice when compared with single mutant a-syn mice (Fig. S9), due to limited availability of samples collected. We appreciate the limitations associated with our study. To further implement reviewer's and editor's comments, we revised the manuscript to leave the questions open as to whether the*

increased size of P-a-syn directly drives the pathology and our current data do not distinguish between a direct effect on α-synuclein aggregation and a more general worsening of late-stage disease.

We agree with reviewer that increased α-synuclein aggregation is not that obvious directly from the immunoblots. We now made a note in the figure legend that it is the relative reduction of the monomers that causes an increase in the overall oligomer/monomer ratio in the double mutant mice.

Regarding the apparently predominant presence of oligomeric species in the Triton-soluble fraction, this is due to the heavy loading of the protein samples on the gel as reflected by total protein staining.

The statement ", which was marginally aggravated by clogger expression (Figure S8A-B)" is now changed to "which was not aggravated by clogger expression (Figure S8A-B)". Thank you for pointing this out.

3. The transcriptomic and proteomic analyses constitute an important component of the mechanistic framework proposed by the authors, yet several aspects of these datasets remain difficult to evaluate. Most importantly, sample sizes and biological replication are not always clearly stated, and in several figures the statistical analyses are either absent or insufficiently described (see also point 4). Given that many of the reported changes are relatively modest, it is difficult to assess the robustness and biological significance of the findings.
In addition, several key conclusions rely heavily on large-scale omics datasets without independent validation. For example, the proposed activation of proteostatic stress pathways and proteasomal responses would be substantially strengthened by orthogonal validation of selected targets using quantitative PCR, immunoblotting, or other targeted approaches. Likewise, validation of proteins identified in the insoluble proteome would help establish the biological relevance of the proteomics findings.

<u>Response</u> -

- *We apologize for the lack of clarity on the sample size and biological replication for the transcriptomic and proteomic analyses. These have been added to the text, figure legends, and methods sections.*
- *Using the quantitatively sensitive RNA-seq data, we detected a statistically significant upregulation of genes encoding proteasomal function in striatum from the clogger mice. As reviewer pointed out, these changes are moderate, reducing the feasibility of independent validation by other methods. We made a comment on*

this in the revised manuscript. We appreciate reviewer's comments on this and the weakness of the data on this particular point. On the other hand, we validated the activation of Hspa1b, Igf2 and Igfbp6 in spinal cord using quantitative reverse PCR (Figure S4A). The data provided support for the RNA-seq data.

4. The statistical analysis and reporting require substantial clarification.
First, male and female mice were pooled for most analyses. While this approach is acceptable when sex does not significantly influence the measured outcome, the study appears underpowered to rigorously assess sex-dependent effects. In several experiments, the number of animals per sex appears low and is not always clearly stated. Consequently, the absence of a statistically significant sex effect should not necessarily be interpreted as evidence that sex differences are absent. Under these circumstances, pooling male and female animals may obscure biologically meaningful sex-dependent effects and should therefore be justified with appropriate statistical analyses. This concern is particularly relevant because the authors report that "clogger mice demonstrate sex-dependent increases in locomotor activity, but no changes in anxiety-like behavior or memory". The authors should provide the sex-specific data and statistical analyses.
Second, the choice of statistical tests appears questionable in several instances and requires further justification. For example, the rationale for using repeated-measures ANOVA in Figures 1A-H, 1J, and 1K is unclear. Likewise, Figures 3E and 5B-D appear more appropriately analyzed using ANOVA models rather than multiple Student t-tests, given the underlying experimental design. The manuscript would also benefit from a clearer description of multiple-comparison correction procedures, which are not consistently reported.
Third, statistical reporting throughout the manuscript is incomplete. Several figures report only selected comparisons, while others appear to lack statistical analyses altogether. Whenever appropriate, the authors should report the key genotype comparisons, including WT vs. clogger, WT vs. α-synuclein(A53T), WT vs. α-synuclein(A53T)/clogger, and α-synuclein(A53T) vs. α-synuclein(A53T)/clogger. In addition, the reported p-values for Figures 3B-C appear inconsistent between the main text, figure panels, and figure legends. These discrepancies need to be resolved and all relevant statistical comparisons should be displayed directly in the figures.
Finally, the number of biological replicates is not always apparent. In several figures, including Figure 2C and Figure 5E-F, it is unclear whether individual data points represent biological replicates or technical measurements. Likewise, the number of samples included in the transcriptomic and proteomic analyses should be stated explicitly in both

the Methods and figure legends.

<u>Response</u> – *We thank the reviewer for these comments, and have addressed them one at a time below.*

Male and female pooling –We note that the number of animals per sex per genotype was already stated in the figure legend for every behavioral mouse test. We reviewed our previous analyses. Where appropriate, we have reformatted the presented data by sex due to small animal numbers (Figure S2A-B) unless already separated by sex in the Figures of the previous version (which is the case for Figure S2 C-E and S3A-E, the latter of which the reviewer specifically requested sex-specific data given sex-specific changes in locomotor activity). For all other figures with substantial *n* per sex per genotype, we included the specific *p*-values for main effect of sex from the preceding two-way (Figure S3G-H) or three-way ANOVAs (Figures 1I, 3B-C, S3G-H, and S10A-G). We note that there were also no significant interaction effects between sex and genotype in these analyses. Lastly, for the prior Figure 1K, we missed a significant effect of sex for this assay. Given this, and that any potentially improved executive function in clogger mice is not important for the main conclusions of the paper, we have excluded these experiments from the revised version of the manuscript. We appreciate the reviewer's attention to detail on this point.

Choice of statistical tests – repeated measures ANOVA was performed in Figures 1A-H as multiple measurements were taken per mitochondrial extract, and we wanted to confirm that there was no effect on order of measurement as mitochondrial extracts are known to "age" while on ice and lose respiratory capacity. This was confirmed, as noted in the figure legend. For Figure 1I, each mouse was ran on the rotarod for several trials, so a repeated measures ANOVA is most appropriate with trial number as the within-subjects variable. Regarding the multiple comparisons testing, the specific correction method used was added to the figure legend (Figure S10) where not already reported (Figures 3B-C and S3A). If there was no significant effect from either genotype, then subgroup analysis was not performed, hence not reported (e.g. Figure S3B-G). We very much apologize for the inconsistent *p* values in Figures 3B-C, this has been corrected. Lastly, we appreciate the point regarding multiple *t* tests for figures 3E and 5B-D. In our view, the only biologically relevant comparison is between the paralyzed mice (α-syn only vs α-syn + clogger), as the other non-paralyzed mice are shown for completeness as negative controls. As such, we have eliminated other *t* tests shown in Figure 3E. Along similar lines, however, we do think the simple *t* test performed in Figure S8B is inappropriate; we performed an ANOVA testing that did not show any significant changes (now noted in the manuscript). We opted for a

simple one-way ANOVA given a full three-way ANOVA is prevented by some groups being structurally empty (i.e. there are no paralyzed mice lacking the α-syn transgene).

Biological replicates – All data points in all figures represent biological replicates. This has been clarified in the figure legends.

Selected comparisons p values – for the sake of clarity, it is our preference to not clutter every figure with numerous *p* values, which we believe are unnecessary for interpretation of the data (e.g. WT vs Clogger when there is clearly not an effect).

5. Numerous figure labels appear misaligned or displaced, in some cases preventing interpretation of the underlying data. This issue affects several main and supplementary figures and must be corrected before the manuscript can be adequately evaluated.

<u>*Response*</u> – *The files were not fully rasterized before being edited in photoshop, creating aberrant images. We apologize for this.*

Minor Comments:

• The authors should label the up/downregulated transcripts/pathways in the heatmap in Figure 2A.

<u>*Response*</u> – fixed.

• How do the authors explain that α-synuclein mice have higher anxiety scores (Figure S5A) but behave normally in the edge/center zone analysis (Figure S5B). Along these lines, why is anxiety not affected by double mutant in Figure S5A compared to α-synuclein mice but in Figure S5B there is a difference. Why is this difference isolated to the middle distances?

<u>*Response*</u> – *These are interesting questions! Figure S5 is renamed to Figure S10 in the new version. Panel A shows reduced time in the center for both the a-syn mice and a-syn + Clogger mice relative to the WT or Clogger, while Panel B shows that the a-syn + Clogger mice may also exhibit thigmotaxis, or a high affinity for the edge of the chamber, while the α-syn mice do not. Since both of these genotype groups also show increased speed of movement relative to the WT and Clogger mice (Panel C), this could be interpreted as simply an intermediate phenotype in the a-syn mice. In other words, these mice may be anxious enough to avoid the exposed center, but not stressed enough to trigger the thigmotaxis seen in the double mutant mice. We hope this helps to explain the complex behavioral data associated with the α-syn mice.*

• “Table S4 is hard to interpret as UniProt identifiers instead of gene symbols are provided. It is also unclear what the ratio A/D means.

Response – *We are very sorry for this as a wrong table was uploaded. The right table with gene symbols is now submitted. (D)/(A) ratio denotes the ratio of proteins detected in the insoluble fractions of α-syn + clogger double (D) mutant versus α-syn (A) mice. This is now explained in the supporting information.*

• All tables lack supporting information and the corresponding raw data.

Response – *Supporting information are added to the tables. The large raw data for RNA-Seq analysis have been deposited to NCBI Gene Expression Omnibus/Sequence Read Archive with the accession numbers GSE236975 (reviewer access token: uzyzukgandydpid). The raw data for mass spectrometry proteomics have been deposited to the ProteomeXchange Consortium via the PRIDE [1] partner repository with the dataset identifier PXD043657 and 10.6019/PXD043657. Reviewer Account details are described in the Method section.*

SUMMARY – Again, we thank reviewers for taking time in evaluating our work and making constructive suggestions that help improve the manuscript.

October 13, 2026

RE: Life Science Alliance Manuscript #LSA-2026-03770R

Prof. Xin Jie Chen
SUNY Upstate Medical University
Biochemistry and Molecular Biology
750 East Adams Street
Syracuse, New York 13210

Dear Dr. Chen,

Thank you for submitting your revised manuscript entitled "Mitochondrial protein import stress augments alpha-synuclein aggregation and neural damage". We apologise for the delay in communicating a decision due to reviewer availability issues.

Your manuscript was evaluated by one of the original reviewers. This reviewer has acknowledged your revisions in addressing most of their concerns but has elaborated on some pending issues.

Based on their overall assessment, we would be happy to publish your paper in Life Science Alliance pending resolution of the following (reviewers') points and final revisions necessary to meet our formatting guidelines.
-This reviewer comments (points 1, 2) that you must soften the claim on direct effects of import stress in the ANT 1 mutant and in relation to CNS phenotypes whilst expanding the discussion to account for other possibilities. You may address these in a manner of your choosing.
-We agree that you must address their concern (point 4) on inflammatory gliosis and depletion of neural stem cells.
-Your revised manuscript must resolve the inconsistencies pointed by the reviewer (point 3- S4B and S4D, and point 5- in description for S14 with proteomics data). Further, Figure S14 must include the blot for VDAC and a clearer image for TIMM23. We acknowledge that you have already provided source data for blots in Figure S14.
-We concur with this reviewer's suggestion on including information on α-synuclein aggregation in the description in the main text.

Please submit a manuscript with changes highlighted and a point-by-point response to the reviewer's comments.

MANUSCRIPT ORGANIZATION AND FORMATTING:

To avoid unnecessary delays in the acceptance and publication of your paper, please read the following information carefully. Full guidelines are available on our Instructions for Authors page, https://www.life-science-alliance.org/authors

Submission of a paper that does not conform to Life Science Alliance guidelines will delay the acceptance of your manuscript.

-Please include a scale bar and size information for Figure 3D (high magnification).
-Please add the X and Bluesky handles of your host institute/organization, as well as your own and/or one of the authors, in our system.
-Please upload a clean manuscript file. The version that highlights changes, please upload as "Related Manuscript file".
-There is a call-out for Figure S6A. However, this figure has only one panel. Please correct.
-Please add callouts for Figures S1A-H; S9A-F and S10A-F to your main manuscript text.
-Please be sure that the authorship listing and order are correct.

We encourage our authors to provide original source data, particularly uncropped/-processed electrophoretic blots and spreadsheets for the main figures of the manuscript. If you would like to add source data, we would welcome one PDF/Excel-file per figure for this information. These files will be linked as supplementary "Source Data" files.

It is Life Science Alliance policy that if requested, original data images must be made available to the editors. Failure to provide original images upon request will result in unavoidable delays in publication. Please ensure that you have access to all original data images prior to final submission.

If you are planning a press release on your work, please inform us immediately to allow informing our production team and scheduling a release date.

We welcome submissions of potential cover images for the issue of LSA in which your work would appear. If you have high quality images associated with this work, please feel free to email these, with a caption, to the journal office.

LSA encourages authors to provide a 30-60 second video where the study is briefly explained. These videos will be appear embedded with the manuscript online at Life Science Alliance, and on social media to promote the published paper and authors (for examples, see https://docs.google.com/document/d/1-UWCfbE4pGcDdcgzcmiuJl2XMBJnxKYeqRvLLrLSo8s/edit?usp=sharing). Corresponding or first-authors are welcome to submit the video. Please submit only one video per manuscript. The video can be emailed to contact@life-science-alliance.org

FINAL FILES:

To upload the final version of your manuscript, please log in to your account: https://lsa.msubmit.net/cgi-bin/main.plex
You will be guided to complete the submission of your revised manuscript and to fill in all necessary information. Please get in touch in case you do not know or remember your login name.

The following items are required for acceptance.

-- An editable version of the final text (.DOC or .DOCX) is needed for copyediting (no PDFs).

-- High-resolution figure, supplementary figure and video files uploaded as individual files: See our detailed guidelines for preparing your production-ready images, https://www.life-science-alliance.org/authors

-- Summary blurb (enter in submission system): A short text summarizing in a single sentence the study (max. 200 characters including spaces). This text is used in conjunction with the titles of papers, hence should be informative and complementary to the title. It should describe the context and significance of the findings for a general readership; it should be written in the present tense and refer to the work in the third person. Author names should not be mentioned.

The license to publish form must be signed before your manuscript can be sent to production. A link to the license to publish form will be available to the corresponding author only. Please take a moment to check your funder requirements.

Reviews, decision letters, and point-by-point responses associated with peer-review at Life Science Alliance will be published, alongside the manuscript. If you do want to opt out of having the reviewer reports and your point-by-point responses displayed, please let us know immediately.

Thank you for your attention to these final processing requirements. Please revise and format the manuscript and upload materials as soon as you are able.

Thank you for this interesting contribution to the literature. We look forward to publishing your paper in Life Science Alliance.

Sincerely,

Sarita Hebbar, PhD
Scientific Editor
Life Science Alliance
http://www.lsajournal.org

Reviewer #3 (Comments to the Authors (Required)):

The authors have addressed most of my previous concerns. However, several points remain that should be addressed before publication:

1. Both the Abstract and Discussion attribute several phenotypes specifically to the clogging/import-stress phenotype caused by mutant ANT1. However, the study does not directly demonstrate mitochondrial protein-import clogging or mitochondrial import stress in the CNS. The wording should therefore distinguish between what is directly demonstrated and what is inferred mechanistically. For example, statements that "ANT1-induced import stress" or "clogging" causes a particular CNS phenotype should be replaced by the more conservative formulation that expression of mutant ANT1 is associated with or causes the observed phenotype, unless import stress has been demonstrated directly in the relevant tissue.

2. Related to the previous point, the Discussion should more explicitly consider the possibility that at least some of the observed phenotypes result from altered ANT1 function itself rather than specifically from a mitochondrial protein-import clogging phenotype. These possibilities are not mutually exclusive, but the current manuscript appears to favor the clogging mechanism without adequately discussing alternative consequences of mutant ANT1 expression. This limitation and alternative interpretation should be addressed.

3. When only six genes are differentially expressed in the striatum (and only five appear to be shown in Figure S4B), it is unclear how the pathway analysis in Figure S4D reveals an apparent upregulation of numerous proteasomal subunits. Are these individual changes statistically significant, or does the pathway-level signal arise from coordinated but individually non-significant changes? This should be clarified. Moreover, if this represents a biologically relevant response to the ANT1 phenotype, a similar proteasomal signature might be expected in the more strongly affected tissues, particularly the spinal cord and cerebellum. It is therefore surprising that the equivalent analysis is not shown for these tissues, given that the relevant datasets are already available.

4. The authors suggest the presence of inflammatory gliosis that leads to depletion of neural stem cells (NSCs). The presented data do not support this interpretation. At 30 months of age, the NSC population is increased rather than decreased in clogger mice, whereas microglial infiltration remains elevated (Figure S6). Furthermore, 30-month-old clogger mice display a more pronounced UPS signature (Figure S5). Together, these observations argue against a straightforward model in which inflammatory gliosis or the UPS phenotype causes NSC depletion. The authors should revise this interpretation and distinguish observations directly supported by the data from mechanistic speculation.

5. In Figure S14, the authors claim enrichment of SDHA, SDHB, TIMM23, and VDAC in the Triton-insoluble fraction. However, the VDAC panel is missing, and the TIMM23 blot appears to be cropped at the relevant molecular weight. Consequently, the VDAC result cannot be evaluated, and quantification of TIMM23 cannot be considered reliable if part of the relevant band is not shown. This point is particularly important because none of these proteins is significantly enriched in the insoluble proteome shown in Figure 7, despite being readily detected (Table S6). The authors should provide complete, uncropped blots and reconcile the biochemical fractionation results with the proteomics data.

6. The rebuttal letter states: "We agree with reviewer that increased α-synuclein aggregation is not that obvious directly from the immunoblots. We now made a note in the figure legend that it is the relative reduction of the monomers that causes an increase in the overall oligomer/monomer ratio in the double mutant mice." This is an important qualification of the interpretation and should be stated explicitly in the main text rather than only in the figure legend.

7. The rebuttal letter states: "Lastly, for the prior Figure 1K, we missed a significant effect of sex for this assay. Given this, and that any potentially improved executive function in clogger mice is not important for the main conclusions of the paper, we have excluded these experiments from the revised version of the manuscript." While these data may not be essential to the main conclusions, the observed sex effect could be informative for future studies. Rather than removing the experiment entirely, I recommend retaining the data, for example in the supplementary material, together with the appropriate sex-stratified analysis.

8. Figure 2C contains typographical errors in the word "months."

9. Line 282 reports a p-value of $p = 0.44$, whereas the corresponding figure reports $p = 0.45$. These repeated inconsistencies should be resolved.

Response to Reviewers Comments

1. Both the Abstract and Discussion attribute several phenotypes specifically to the clogging/import-stress phenotype caused by mutant ANT1. However, the study does not directly demonstrate mitochondrial protein-import clogging or mitochondrial import stress in the CNS. The wording should therefore distinguish between what is directly demonstrated and what is inferred mechanistically. For example, statements that "ANT1-induced import stress" or "clogging" causes a particular CNS phenotype should be replaced by the more conservative formulation that expression of mutant ANT1is associated with or causes the observed phenotype, unless import stress has been demonstrated directly in the relevant tissue.

Response – Agree. We replaced "ANT1-induced import stress" and "clogging" with “Ant1P.A114P,A123D expression” as much as we can in the text.

2. Related to the previous point, the Discussion should more explicitly consider the possibility that at least some of the observed phenotypes result from altered ANT1 function itself rather than specifically from a mitochondrial protein-import clogging phenotype. These possibilities are not mutually exclusive, but the current manuscript appears to favor the clogging mechanism without adequately discussing alternative consequences of mutant ANT1 expression. This limitation and alternative interpretation should be addressed.

Response – We extended the discussion to cover the point raised by reviewer.

3. When only six genes are differentially expressed in the striatum (and only five appear to be shown in Figure S4B), it is unclear how the pathway analysis in Figure S4D reveals an apparent upregulation of numerous proteasomal subunits. Are these individual changes statistically significant, or does the pathway-level signal arise from coordinated but individually non-significant changes? This should be clarified. Moreover, if this represents a biologically relevant response to the ANT1 phenotype, a similar proteasomal signature might be expected in the more strongly affected tissues, particularly the spinal cord and cerebellum. It is therefore surprising that the equivalent analysis is not shown for these tissues, given that the relevant datasets are already available.

Response – We made a note in the text. As transcriptional changes to proteostatic genes in spinal cord are much stronger and can be identified based on the analysis of individual genes, the pathway-level analysis was therefore not undertaken.

4. The authors suggest the presence of inflammatory gliosis that leads to depletion of neural stem cells (NSCs). The presented data do not support this interpretation. At 30 months of age, the NSC population is increased rather than decreased in clogger mice, whereas microglial infiltration remains elevated (Figure S6). Furthermore, 30-month-old clogger mice display a more pronounced UPS signature (Figure S5). Together, these observations argue against a straightforward model in which inflammatory gliosis or the UPS phenotype causes NSC depletion. The authors should revise this interpretation and distinguish observations directly supported by the data from mechanistic speculation.

Response – We deleted the sentence regarding NSC depletion at 24-month-old mice. Thanks.

5. In Figure S14, the authors claim enrichment of SDHA, SDHB, TIMM23, and VDAC in the Triton-insoluble fraction. However, the VDAC panel is missing, and the TIMM23 blot appears to be cropped at the relevant molecular weight. Consequently, the VDAC result cannot be evaluated, and quantification of TIMM23 cannot be considered reliable if part of the relevant band is not shown. This point is particularly important because none of these proteins is significantly enriched in the insoluble proteome shown in Figure 7, despite being readily detected (Table S6). The authors should provide complete, uncropped blots and reconcile the biochemical fractionation results with the proteomics data.

Response – We apologize for the accidental omission of the VDAC blot. It is now added back. The TIM23 band is weak but uncropped.

6. The rebuttal letter states: "We agree with reviewer that increased α-synuclein aggregation is not that obvious directly from the immunoblots. We now made a note in the figure legend that it is the relative reduction of the monomers that causes an increase in the overall oligomer/monomer ratio in the double mutant mice." This is an important qualification of the interpretation and should be stated explicitly in the main text rather than only in the figure legend.

Response – We moved the note to the manuscript text.

7. The rebuttal letter states: "Lastly, for the prior Figure 1K, we missed a significant effect of sex for this assay. Given this, and that any potentially improved executive function in clogger mice is not important for the main conclusions of the paper, we have excluded these experiments from the revised version of the manuscript." While these data may not be essential to the main conclusions, the observed sex effect could be informative for future studies. Rather than removing the experiment entirely, I recommend retaining the

data, for example in the supplementary material, together with the appropriate sex-stratified analysis.

Response – We added the data back (Fig. 1J) as suggested. We also made a note regarding sex stratified analysis.

8. Figure 2C contains typographical errors in the word "months."

Response – Fixed

9. Line 282 reports a p-value of p = 0.44, whereas the corresponding figure reports p = 0.45. These repeated inconsistencies should be resolved.

Response - Fixed.

Again, we thank reviewers for taking time to evaluate our work and making constructive suggestions that help improve the manuscript.

October 22, 2026

RE: Life Science Alliance Manuscript #LSA-2026-03770RR

Prof. Xin Jie Chen
SUNY Upstate Medical University
Biochemistry and Molecular Biology
750 East Adams Street
Syracuse, New York 13210

Dear Dr. Chen,

Thank you for submitting your Research Article entitled "Mitochondrial protein import stress augments alpha-synuclein aggregation and neural damage". It is a pleasure to let you know that your manuscript is now accepted for publication in Life Science Alliance. Congratulations on this interesting work.

The final published version of your manuscript will be deposited by us to PubMed Central upon publication.

Your manuscript will now progress through copyediting and proofing. It is journal policy that authors provide original data upon request.

Your article will publish open access upon publication under a CC-BY license.

Reviews, decision letters, and point-by-point responses associated with peer-review at Life Science Alliance will be published, alongside the manuscript. If you do want to opt out of having the reviewer reports and your point-by-point responses displayed, please let us know immediately.

IMPORTANT: If you will be unreachable at any time, please provide us with the email address of an alternate author. Failure to respond to routine queries may lead to unavoidable delays in publication.

Scheduling details will be available from our production department. You will receive proofs shortly before the publication date. Only essential corrections can be made at the proof stage so if there are any minor final changes you wish to make to the manuscript, please let the journal office know now.

DISTRIBUTION OF MATERIALS:
Authors are required to distribute freely any materials used in experiments published in Life Science Alliance. Authors are encouraged to deposit materials used in their studies to the appropriate repositories for distribution to researchers.

You can contact the journal office with any questions, contact@life-science-alliance.org

Again, congratulations on a very nice paper. I hope you found the review process to be constructive and are pleased with how the manuscript was handled editorially. We look forward to future exciting submissions from your lab.

Sincerely,

Sarita Hebbar, PhD
Scientific Editor
Life Science Alliance
http://www.lsajournal.org
