# Supplementary material for: Mitochondrial protein import stress augments α-synuclein aggregation and neural damage
Source: Life Sci Alliance. 2026 Nov 10;10(1):e202603770. doi: 10.26508/lsa.202603770 (PMC13752406; doi:10.26508/lsa.202603770)
Supplement: Supplementary file 5 [file LSA-2026-03770_TableS4.docx]

| **Supplemental Table 4. Changes in microglial-specific RNAs between 30-month-old clogger and wild type mice compared to all genes.** | | | | |
| --- | --- | --- | --- | --- |
| **Region** | **Mean Log_2_ Diff All Genes** | **Mean Log_2_ Diff Microglial Genes** | **p-value** | **Interpretation** |
| Spinal Cord | 0.00087 | 0.11992 | 0.00071 | Highly Significant |
| Cerebellum | 0.00202 | -0.0054 | 0.79436 | Not Significant |
| Striatum | -0.00096 | 0.04007 | 0.08437 | Trend |
