# Supplementary material for: Mitochondrial protein import stress augments α-synuclein aggregation and neural damage
Source: Life Sci Alliance. 2026 Nov 10;10(1):e202603770. doi: 10.26508/lsa.202603770 (PMC13752406; doi:10.26508/lsa.202603770)
Supplement: Supplementary file 6 [file LSA-2026-03770_TableS5.docx]

| **Supplemental Table 5. Log_2_ expression changes of microglial RNAs in 30-month-old *Clogger* vs WT mice.** | | | |
| --- | --- | --- | --- |
| **Symbol** | **Spinal Cord** | **Cerebellum** | **Striatum** |
| Aif1 | 0.0237 | -0.1252 | 0.0459 |
| C1qa | 0.2595 | 0.0733 | 0.1273 |
| C1qb | 0.2075 | 0.0341 | 0.1667 |
| C1qc | 0.1939 | 0.0525 | 0.1281 |
| Cd14 | 0.18 | -0.2865 | 0.034 |
| Csf1r | 0.0929 | -0.0638 | -0.0029 |
| Cx3cr1 | 0.1213 | -0.0204 | -0.0215 |
| Fcgr3 | 0.1761 | 0.0096 | 0.0561 |
| Gpr34 | 0.1775 | 0.0961 | -0.1218 |
| Mpeg1 | 0.1961 | -0.0058 | 0.0654 |
| P2ry13 | 0.0648 | 0.1007 | -0.0165 |
| Siglech | 0.0161 | 0.0001 | 0.0627 |
| Sparc | -0.1036 | -0.0988 | -0.1194 |
| Tmem119 | -0.0378 | -0.0007 | 0.0644 |
| Trem2 | 0.2308 | 0.1537 | 0.1325 |
